# The epidemiology of hepatitis C virus in Iran: Systematic review and meta-analyses

**DOI:** 10.1038/s41598-017-18296-9

**Published:** 2018-01-09

**Authors:** Sarwat Mahmud, Vajiheh Akbarzadeh, Laith J. Abu-Raddad

**Affiliations:** 1 Infectious Disease Epidemiology Group, Weill Cornell Medical College - Qatar, Cornell University, Qatar Foundation - Education City, Doha Qatar; 2 Department of Healthcare Policy and Research, Weill Cornell Medical College, Cornell University, New York, New York USA

## Abstract

The aim of this study was to characterize hepatitis C virus (HCV) epidemiology in Iran and estimate the pooled mean HCV antibody prevalence in different risk populations. We systematically reviewed and synthesized reports of HCV incidence and/or prevalence, as informed by the Cochrane Collaboration Handbook, and reported our findings following the PRISMA guidelines. DerSimonian-Laird random effects meta-analyses were implemented to estimate HCV prevalence in various risk populations. We identified five HCV incidence and 472 HCV prevalence measures. Our meta-analyses estimated HCV prevalence at 0.3% among the general population, 6.2% among intermediate risk populations, 32.1% among high risk populations, and 4.6% among special clinical populations. Our meta-analyses for subpopulations estimated HCV prevalence at 52.2% among people who inject drugs (PWID), 20.0% among populations at high risk of healthcare-related exposures, and 7.5% among populations with liver-related conditions. Genotype 1 was the most frequent circulating strain at 58.2%, followed by genotype 3 at 39.0%. HCV prevalence in the general population was lower than that found in other Middle East and North Africa countries and globally. However, HCV prevalence was high in PWID and populations at high risk of healthcare-related exposures. Ongoing transmission appears to be driven by drug injection and specific healthcare procedures.

## Introduction

Hepatitis C virus (HCV) related morbidity and mortality places a substantial burden on healthcare systems worldwide^1,2^. While viral hepatitis is the seventh leading cause of death globally, it is the fifth leading cause of death in the Middle East and North Arica (MENA), predominantly due to HCV^3^. High HCV antibody prevalence levels are found in few MENA countries^4,5^, mainly in Pakistan, at 4.8%^6–8^, and Egypt, at 14.7%^9,10^. Recent major breakthroughs in HCV treatment, in the form of Direct Acting Antivirals (DAA), have provided promising prospects for reducing HCV transmission and disease burden^11,12^. Elimination of HCV as a public health problem by 2030 has recently been set as a global target by the World Health Organization (WHO)^13,14^.

While HCV epidemiology in MENA countries, such as Egypt and Pakistan, has been studied in depth^6,7,9,10,15^, HCV epidemiology in Iran remains not well-characterized. Iran is estimated to have the highest population proportion of people who inject drugs (PWID) in MENA^16^, a key population at high risk of HCV infection. Iran shares a border with Afghanistan, the world’s largest opiates producer^17^, and therefore has become a major transit country for drug trafficking^18^. Nearly half of opium, heroine, and morphine seizures globally occur in Iran alone^18^. Increased availability and lower prices of injectable drugs have led to increased injecting drug use and dependency^19,20^. Understanding HCV epidemiology in Iran is critical for developing and targeting cost-effective and cost-saving prevention and treatment interventions against HCV.

The aim of this study was to characterize HCV epidemiology in Iran by (1) systematically reviewing and synthesizing records, published and unpublished, of HCV incidence and prevalence among the different population groups, (2) systematically reviewing and synthesizing evidence on HCV genotypes, and (3) estimating pooled mean HCV prevalence among the general population and other key risk populations by pooling available HCV prevalence measures. This study is conducted as part of the MENA HCV Epidemiology Synthesis Project, an on-going effort to characterize HCV epidemiology in MENA, providing empirical evidence to inform key public health research, policy, and programming priorities at the national and regional level^5,7,9,21–30^.

## Materials and Methods

This study follows the methodology used in the previous systematic reviews of the MENA HCV Epidemiology Synthesis Project^7,9,21–25,27^. The following subsections summarize this methodology while further details can be found in previous publications of this project^7,9,21–25,27^.

### Data sources and search strategy

We systematically reviewed all HCV incidence and prevalence data in Iran as informed by the Cochrane Collaboration Handbook^31^. We reported our results using the Preferred Reporting Items for Systematic reviews and Meta-analyses (PRISMA) guidelines (Table S1)^32^. Our main data sources included PubMed and Embase databases (up to June 27^th^, 2016), the Scientific Information Database (SID) of Iran (up to June 29^th^, 2016), the World Health Organization Index Medicus for the Eastern Mediterranean Region (IMEMR WHO) database (up to July 1^st^, 2016), and the abstract archive of the International Aids Society (IAS) conferences (up to July 1^st^, 2016). Additionally, the MENA HIV/AIDS Epidemiology Synthesis Project database was searched for further records in the form of country level reports and routine data^33,34^. A broad search criteria was used (Fig. S1) with no language restrictions. Articles were restricted to those published after 1989, the year in which HCV was first identified^35,36^.

### Study selection

**Figure 1:**
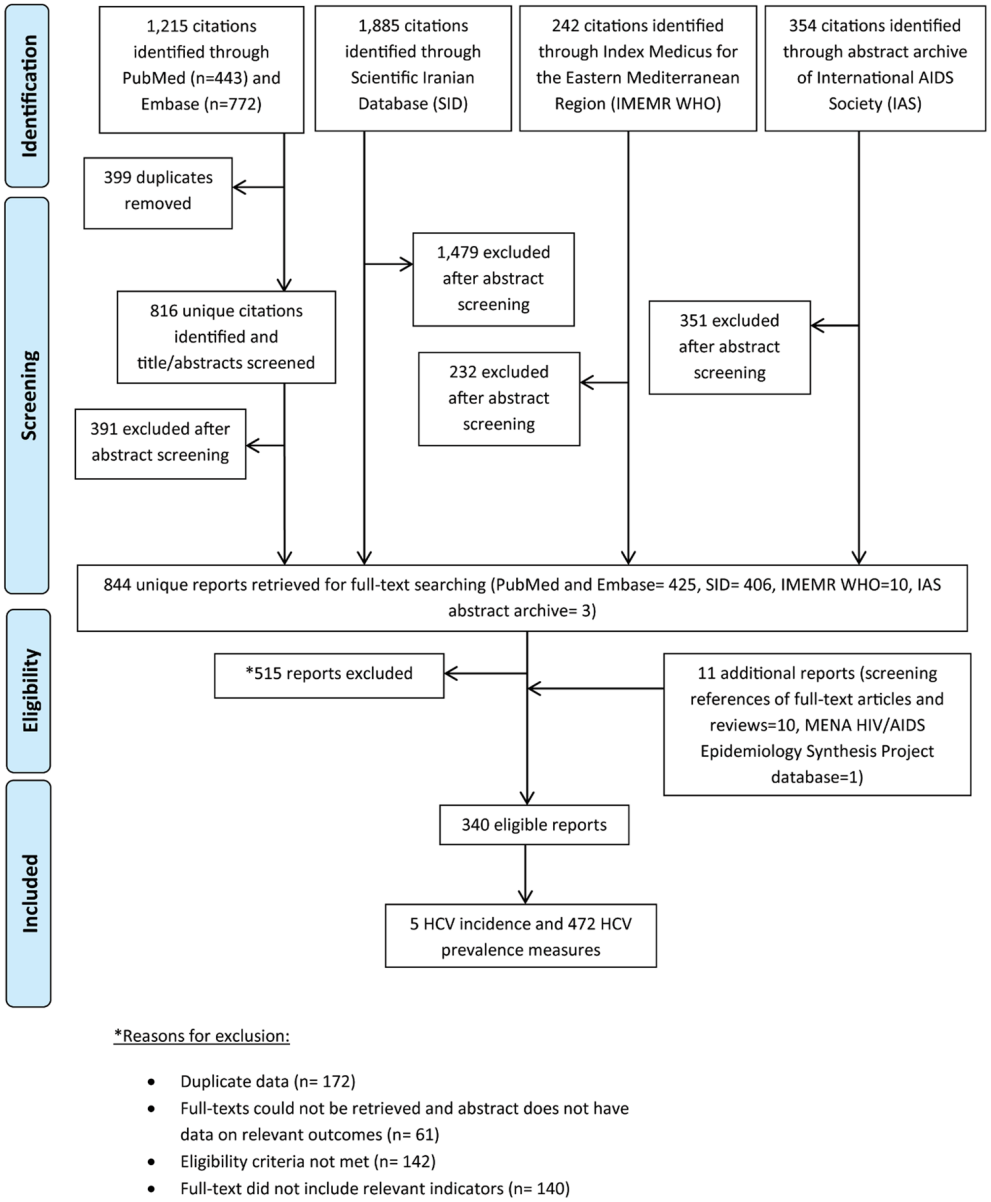
Flow chart of article selection for the systematic review of hepatitis C virus (HCV) incidence and prevalence in Iran, adapted from the PRISMA 2009 guidelines^32^.

All records identified through our search were imported into a reference manager, Endnote, where duplicate publications were identified and excluded (Fig. 1). Similar to our previous systematic reviews^7,9,21–25,27^, the remaining unique reports underwent two stages of screening, performed by SM and VA. The titles and abstracts were first screened, and those deemed relevant or potentially relevant underwent further screening, in which the full-texts were retrieved and assessed for eligibility, based on our inclusion and exclusion criteria. Eligible reports were included in this study, while the remaining ineligible reports were excluded for reasons indicated in Fig. 1. The references of all full-text articles and literature reviews were also screened for further potentially relevant reports.

### Inclusion and exclusion criteria

The inclusion and exclusion criteria used were developed based also on our previous systematic reviews^7,9,21–25,27^. Briefly, any document, of any language, reporting HCV antibody incidence and/or antibody prevalence in Iran, based on biological assays and on primary data, qualified for inclusion in this review. Our exclusion criteria included case reports, case series, editorials, letters to editors, commentaries, literature reviews, and studies reporting HCV prevalence based on self-reporting and/or on Iranian nationals outside of Iran. Studies performed before 1989, and studies referring to HCV as non-A non-B hepatitis, were also excluded. A secondary independent screening was also performed for articles reporting HCV genotype information, regardless of whether information on HCV incidence and/or prevalence was included.

In the subsequent sections, any document including outcome measures of interest will be referred to as a ‘report’, while details of a specific outcome measure will be referred to as a ‘study’. Accordingly, one report may contribute multiple studies, and multiple reports of the same study (outcome measure) were identified as duplicates and considered as one study.

### Data extraction and data synthesis

Data from relevant reports were extracted by SM and VA. To check for consistency in extractions, 37% of reports were double extracted. Nature of extracted data followed our previous HCV systematic reviews^7,9,21–25,27^. HCV prevalence measures were rounded to one decimal place, with the exception of those below 1%, which were rounded to two decimal places. Risk factors for HCV infection (at the individual level), which were found to be statistically significant through multivariable regression analyses, were extracted from all articles, when available.

Risk factors for HCV infection were extracted when identified as significant after controlling for confounders through multivariable regression analyses. Data on HCV ribonucleic acid (RNA) prevalence were extracted whenever available in reports including an HCV prevalence measure(s). HCV genotype studies identified through the independent secondary screening were extracted to a separate data file. Extracted data were stratified by study populations’ risk of acquiring HCV infection as follows:

1. General populations (that is populations at low risk): these consisted of blood donors, pregnant women, children, healthy adults, and army recruits, among other general population groups.
2. Populations at intermediate risk: these consisted of healthcare workers, household contacts of HCV infected patients, female sex workers, prisoners, homeless people, and drug users (only where the route of drug use *was not specified or excluded drug injection*), among others. Drug users were classified into the intermediate risk category as we could not assess, with the available information, the extent to which drug injection is common in any such specific population—it is possible that the majority of these drug users were not injecting drugs at the time of the study.
3. Populations at high risk: these consisted of HIV patients, PWID, and of populations at high risk of healthcare-related exposures, such as hemodialysis patients, hemophilia patients, thalassemia, patients, and patients with bleeding disorders.
4. Special clinical populations: these consisted of populations with liver-related conditions, such as chronic liver disease, acute viral hepatitis, hepatocellular carcinoma, and liver cirrhosis. This also consisted of other special clinical populations for which the level of HCV risk of exposure could not be ascertained a priori, such as lichen planus patients.

### Quantitative assessment

The quantitative analyses were conducted following an analysis plan similar to that in our previous HCV systematic reviews^7,9,21–25,27^. HCV prevalence data in reports comprising at least 50 participants were stratified by risk and summarized using reported prevalence measures. Meta-analyses of HCV prevalence measures were conducted by risk category for studies consisting of a minimum of 25 participants. Stratified measures were used in place of HCV prevalence for the total sample only if the sample size requirement was met for each stratum.

A pre-defined sequential order was followed when considering stratifications. Nationality was prioritized, followed by sex, year, region, and age. One stratification was included per study to avoid double-counting.

The variance of the prevalence measures was stabilized using the Freeman-Tukey type arcsine square-root transformation of the corresponding proportions^37^. Estimates for HCV prevalence were weighted using the inverse variance method and then pooled using a DerSimonian-Laird random effects model. This model accounts for sampling variation and expected heterogeneity in effect size across studies^38^. Heterogeneity was assessed using several measures. The forest plots were visually inspected and Cochran’s Q test was conducted, where a p-value < 0.10 was considered significant^38,39^. The I² and its confidence intervals were calculated^38^. The prediction intervals were also calculated to estimate the distribution of true effects around the estimated mean^38,40^.

Univariable and multivariable random-effects meta-regressions, based on established methodology^31^, were conducted to determine population-level associations with HCV prevalence and sources of between-study heterogeneity. Variables entered into the univariable model included risk population, sample size (<100 or ≥100), study site, sampling methodology (probability-based or nonprobability-based), publication year, and median year of data collection. Variables were included into the final multivariable model if the p-value was <0.10. Variables with a p-value < 0.05 in the final multivariable meta-regression were considered significant.

The majority of HCV prevalence measures in the general population were among blood donors, a population that mainly includes healthy adults. Therefore, we performed a sensitivity analysis to ascertain the impact of excluding blood donors on our pooled mean estimate for HCV prevalence among the general population (Fig. S5).

Descriptive analyses of HCV genotypes and subtypes were also performed. Individuals with mixed HCV genotypes contributed to the quantification of each identified genotype separately. Meta-analyses of genotype proportions were also performed to estimate the pooled mean proportions for each genotype. The diversity of HCV genotypes was assessed using the Shannon Diversity Index^41^.

Meta-analyses were performed on R version 3.1.2^42^, using the package *meta* ^43^. Meta-regressions were performed on STATA 13, using the *metan* command^44^.

### Qualitative analysis

Similar to our previous HCV systematic reviews^7,9,21–25,27^, the quality of each incidence or prevalence measure was determined by assessing sources of bias that may affect the reported measure. The Cochrane approach was used to infer the risk of bias (ROB)^31^, and the precision of the reported measures was also evaluated. Studies were categorized into low or high ROB based on three quality domains: type of HCV ascertainment (biological assay or otherwise), rigor of sampling methodology (probability-based or nonprobability-based), and response rate (≥80% of the target sample size was reached or otherwise).

Studies with missing information for any of the three domains were categorized as *unclear* ROB for that specific domain. Studies where HCV measures were obtained from individuals presenting voluntarily to facilities where routine blood screening is conducted, or retrieved from patients’ medical records, were considered as having *low* ROB on the response rate domain. HCV prevalence measures obtained from country-level routine reporting, with limited description of the methodology used to be able to conduct ROB assessment, were categorized as of *unknown* quality.

Studies where HCV measures were obtained from a sample size of at least 100 individuals were considered as having high precision. For an HCV prevalence of 1% and a sample size of 100, the 95% confidence interval (CI) is 0–5%; a reasonable CI for an HCV prevalence estimate.

## Results

### Search results

Figure 1 describes the selection process by which studies were included in this systematic review, adapted from the PRISMA flow diagram^32^. We identified a total of 3,696 citations: 443 from PubMed, 772 from Embase, 1,885 from SID, 242 from IMEMR WHO, and 354 from the abstract archive of the IAS. After exclusion of duplicates and screening of titles and abstracts, 844 unique reports remained, for which the full-texts were retrieved for full-text screening. After full-text screening, 515 reports were excluded for reasons specified in Fig. 1. An additional 10 records were identified through screening references of full-text articles and reviews. One country-level report was retrieved and included from the MENA HIV/AIDS Epidemiology Synthesis Project database^33,34^. In total, 340 eligible reports were included in this systematic review. This yielded five HCV incidence measures and 472 HCV prevalence measures.

All 3,696 citations underwent an independent secondary screening for HCV genotype studies (Fig. S2). After title and abstract screening and exclusion of duplicates, the full-texts of 144 reports were screened. In total, 44 reports were found eligible for inclusion in this secondary systematic review, yielding 66 HCV genotype measures.

### HCV incidence overview

We identified five incidence measures through our search (Table 1), three of which were conducted in Tehran. The highest sero-conversion risks were observed in thalassemia patients and hemodialysis patients, of 6.8% and 4.3%, respectively^45,46^. In special clinical populations, HCV incidence was measured in renal transplant patients and impaired glucose tolerance patients. The HCV sero-conversion risks were 2.1% and 0.71%, respectively^47,48^. In female drug users on methadone treatment (where the route of drug use was not specified) the sero-conversion risk was 2.5%^49^. No studies reported incidence rate, nor provided sufficient information for incidence rate to be calculated.

**Table 1:** Studies reporting hepatitis C virus (HCV) incidence in Iran.

Author, year (citation)	Year of data collection	Study site	Population’s classification based on risk of HCV exposure	Population	Sample size at recruitment	Lost to follow-up	HCV sero-conversion risk (relative to total sample size)	Duration of follow-up
Pourmand, 2007^47^	2002–04	Hospital	Special clinical population	Renal transplant patients	141	0	2.1%	24 months
Jabbari, 2008^46^	2005–06	Hospital	High risk population	Hemodialysis patients	70	0	4.3%	18 months
Azarkeivan, 2012^45^	1996–09	Blood transfusion center	High risk population	Thalassemia patients	307	0	6.8%	168 months
Dolan, 2012^49^	2007–08	Rehab center	Intermediate risk population	Female drug users on methadone treatment	78	38	2.5%	7 months
Bahar, 2007^48^	1998–01	Hospital	Special clinical population	Impaired glucose tolerance patients	560	0	0.71%	36 months

### HCV prevalence overview

#### General population

A total of 122 HCV prevalence measures were identified in the general population (Table 2), ranging from 0.0% to 3.1%, with a median of 0.3%. Most measures were obtained from blood donors (n = 72) where HCV prevalence ranged from 0.0% to 3.1%, with a median of 0.3%. In pregnant women (n = 6), HCV prevalence ranged from 0.0% to 0.8%, with a median of 0.3%. In other general populations (n = 44), HCV prevalence ranged from 0.0% to 2.4%, with a median of 0.5%.

**Table 2:** Studies reporting hepatitis C virus (HCV) prevalence among the general population (populations at low risk) in Iran.

Author, year (citation)	Year(s) of data collection	City or country of survey	Study site	Study design	Study sampling procedure	Population	Sample size	HCV prevalence (%)
Afzali, 2003^108^	1996	Kashan	Blood transfusion center	CS	NS	Blood donors	6,669	0.37
Afzali, 2003^108^	1997	Kashan	Blood transfusion center	CS	NS	Blood donors	6,750	0.64
Afzali, 2003^108^	1998	Kashan	Blood transfusion center	CS	NS	Blood donors	6,922	0.59
Afzali, 2003^108^	1999	Kashan	Blood transfusion center	CS	NS	Blood donors	6,986	1.6
Afzali, 2003^108^	2001	Kashan	Blood transfusion center	CS	NS	Blood donors	7,721	1.7
Afzali, 2003^108^	2000	Kashan	Blood transfusion center	CS	NS	Blood donors	8,683	1.5
Aghanjanipoor, 2006^109^	2002	Babol	Blood transfusion center	CS	Conv	Blood donors	16,576	0.48
Alavi, 2012^110^	NS	Tehran	NS	CC	Conv	Healthy children	90	0.00
Alavian, 2002^111^	1996–1998	Tehran	Blood transfusion center	CC	Conv	Blood donors	319,375	0.09
Alavian, 2015^112^	2012	Isfahan	Clinical: hospital & health care centers	CC	Conv	Healthy adults	275	0
Amini, 2005^113^	NS	Tehran	Blood transfusion center	CS	Conv	Blood donors	100	0.00
Ansar, 2002^114^	1997–1998	Rasht	Blood transfusion center	CS	SRS	Blood donors	5,976	0.03
Ansari-Moghaddam, 2012^115^	2008–2009	Zahedan	Primary health care centers (community)	CS	Cluster sampling	Residents (male)	1,207	0.66
Ansari-Moghaddam, 2012^115^	2008–2009	Zahedan	Primary health care centers (community)	CS	Cluster sampling	Residents (female)	1,380	0.36
Ardebili, 2012^116^	2007–2011	Kavar	Community	CS	Conv	General population	6,095	0.24
Arfaee, 2002^117^	NS	Tehran	Clinical: hospital & health care centers	CS	Conv	Veterans	307	0.97
Assarehzadegan, 2008^118^	2005	Khuzestan	Blood bank	CS	Conv	Blood donors	400	0.00
Babak, 2008^119^	2006	Kermanshah	Community	CS	Cluster sampling	Residents	1,721	0.87
Barhaghtalab, 2008^120^	2002–2007	Fasa	Blood transfusion center	CS	Conv	Blood donors	25,491	0.55
Bozorgi, 2012^121^	2009	Ghazvin	Blood bank	CS	Conv	Blood donors	20,591	0.17
Chamani, 2007^122^	2004	Tehran	Fertility clinic/IVF	NS	NS	Infertile individuals (female)	533	0.40
Chamani, 2007^122^	2004	Tehran	Fertility clinic/IVF	CS	NS	Infertile individuals (male)	716	0.00
Delavari, 2004^123^	2003	Kerman	Blood transfusion center	CS	NS	Blood donors (female)	2,921	0.10
Delavari, 2004^123^	2003	Kerman	Blood transfusion center	CS	NS	Blood donors (male)	12,331	0.46
Doosti, 2009^124^	2003–2004	Shahre-Kord	Regional blood transfusion center	CS	Conv	Blood donors	11,200	0.59
Emam, 2006^125^	2001–2003	Jahrom	Blood bank	CS	Conv	Blood donors	3,000	0.30
EMRO, 2011^126^	2011	National	National	CS	Conv	Blood donors	1,986,992	0.06
Esfandiarpour, 2005^127^	2002–2003	Kerman	NS	CC	NS	General population	149	1.3
Esmaeili, 2004^128^	2004	Babol	Clinical: hospital & health care centers	CC	NS	Children not receiving blood	100	0.00
Esmaeili, 2004^128^	2004	Babol	Clinical: hospital & health care centers	CC	NS	Children receiving blood	100	2.0
Esmaeili, 2009^129^	2006–2007	Bushehr	NS	CS	Conv	Blood donors	20,294	0.21
Farajzadeh, 2005^130^	2001–2002	Kerman	Blood transfusion center	CC	Conv	Blood donors	96	3.1
Farshadpour, 2010^131^	2007–2007	Ahvaz	Regional blood transfusion center	CS	Conv	Blood donors	2,376	2.3
Farshadpour, 2016^132^	2004–2014	Bushehr	Blood transfusion center	CS	Conv	Blood donors	293,454	0.10
Gachkar, 2015^133^	2004	Tabriz	Blood transfusion center	CS	Conv	Blood donors (male)	399	0.00
Gerayli, 2015^134^	2012	Mashhad	Medical Laboratory	CC	Conv	Healthy adults	134	0
Ghaderi, 2007^135^	2004–2006	Birjand	NS	CC	Conv	Blood donors	150	0.67
Ghadir, 2006^136^	NS	Golestan	Community	NS	NS	General population (male)	736	0.18
Ghadir, 2006^136^	NS	Golestan	Community	NS	NS	General population (female)	1,387	0.85
Ghafouri, 2011^137^	2006–2009	South Khorasan	NS	CS	Conv	Blood donors	95,538	0.01
Ghavanini, 2000^138^	1998	Shiraz	Regional blood transfusion center	CS	Conv	Blood donors	7,879	0.59
Ghezeldasht, 2015^139^	2009–2010	Khorasan Razavi	Community	CS	Cluster sampling	General population	1,227	0.57
Habibzadeh, 2005^140^	2003	Ardabil	Blood transfusion center	CS	Conv	Blood donors	441	0.23
Hajiani, 2006^141^	2003–2004	Tehran	Blood transfusion center	CC	NS	Blood donors	500	1.2
Hajiani, 2006^142^	1998–2003	Ahvaz	NS	CC	Conv	Healthy adults	360	1.0
Heydarabad, 2012^143^	2010–2012	Malekan	NS	NS	NS	Pregnant women	420	0.48
Hosseien, 2009^144^	2003–2005	Tehran	Regional blood transfusion center	CS	Conv	Blood donors	1,004,889	2.1
Hosseini, 2007^145^	2005	Boushehr	NS	NS	NS	Blood donors	19,627	0.23
Jadali, 2005^146^	NS	Tehran	NS	CC	NS	Healthy individuals	50	0.00
Jadali, 2005^147^	NS	NS	NS	CC	Conv	Healthy individuals	50	0.00
Jamali, 2008^148^	2006	Golestan	Community	CS	Cluster sampling	General population	2,049	1.0
Kafi-abad, 2009^149^	2004–2007	National	Blood transfusion center	CS	Conv	Blood donors	6,499,851	0.13
Karim, 2008^150^	2003–2005	Ahvaz	Blood transfusion center	CC	Conv	Blood donors	125	2.4
Karimi, 2008^151^	2004–2006	Shahre-Kord	Blood transfusion center	CS	Conv	Blood donors	35,124	0.20
Kasraian, 2008^152^	2007–2008	Shiraz	Blood transfusion center	CC	Conv	Blood donors	93,987	0.21
Kasraian, 2010^153^	2003	Shiraz	Blood transfusion center	Pre-post	Conv	Blood donors (post-earthquake)	239	0.84
Kasraian, 2010^153^	2003	Shiraz	Blood transfusion center	Pre-post	Conv	Blood donors (pre-earthquake)	1,694	0.47
Kasraian, 2015^154^	2002	Shiraz	Regional blood transfusion center	CS	Conv	Blood donors	NS	0.19
Kasraian, 2015^154^	2003	Shiraz	Regional blood transfusion center	CS	Conv	Blood donors	NS	0.13
Kasraian, 2015^154^	2004	Shiraz	Regional blood transfusion center	CS	Conv	Blood donors	NS	0.09
Kasraian, 2015^154^	2005	Shiraz	Regional blood transfusion center	CS	Conv	Blood donors	NS	0.16
Kavoosi, 2008^155^	2004–2005	Kermanshah	NS	CC	Conv	Healthy adults	57	1.7
Kazeminejad, 2005^156^	2003	Gorgan	Blood transfusion center	CS	Conv	Blood donors	38,920	0.19
Keshvari, 2015^157^	2008	Tehran	Blood transfusion center	CS	Conv	Blood donors	296,567	0.14
Keshvari, 2015^157^	2013	Tehran	Blood transfusion center	CS	Conv	Blood donors	282,010	0.07
Khedmat, 2007^158^	2005–2006	Tehran	Blood transfusion center	CS	SRS	Blood donors	1,014	2.1
Khodabandehloo, 2013^159^	2008–2011	Semnan	NS	CS	Conv	Blood donors	124,704	0.03
Kordi, 2011^160^	NS	Tehran	Community	CC	Cluster sampling	Volleyball and soccer players	410	0.00
Kordi, 2011^160^	NS	Tehran	Community	CC	Cluster sampling	Wrestlers (male)	420	0.48
Mahmoudian, 2006^161^	2003–2004	Mixed (28 provinces unspecified)	Blood transfusion center	CS	Conv	Blood donors	1,489,935	0.07
Maneshi, 2010^162^	2004–2008	Bushehr	Blood transfusion center	CS	Conv	Blood donors	51,884	0.33
Mansour-Ghanaei, 2007^163^	1998–2003	Guilan	Blood transfusion center	CS	Conv	Blood donors	221,508	0.32
Masaeli, 2006^164^	2002–2003	Isfahan	Blood transfusion center	CS	Conv	Blood donors	29,458	0.24
Merat, 2010^54^	2006	Mixed (Golestan, Tehran, Hormozgan)	Community	CS	Cluster sampling	General population	5,684	0.88
Metanet, 2006^165^	2004	Zahedan	NS	CC	Conv	Blood donors	1,399	0.07
Moezzi, 2015^166^	NS	Chaharmahal and Bakhtiari	Community	CS	Single stage cluster sampling	Adults	3,000	1.4
Mogaddam, 2010^167^	NS	Ardabil	Blood bank	CC	NS	Blood donors	60	0.00
Mohammadali, 2014^168^	2005–2011	Tehran	Blood transfusion center	CS	Conv	Blood donors	2,031,451	0.39
Mohebbi, 2011^169^	2007–2008	Lorestan	Primary health care centers (community)	CS	Conv	Pregnant women	827	0.24
Moniri, 2004^170^	2001–2002	Kashan	Blood bank	CS	Conv	Blood donors	600	0.50
Monsour-Ghanaei, 2007^73^	2003	Guilan	Community	CS	Conv	Residents of nursing home	383	2.3
Moradi, 2007^171^	2001–2002	Saravan city, Sistan and Baluchistan	Community	CS	Cluster sampling	Women in childbearing ages	356	0.84
Motlagh, 2001^172^	1999–2000	Ahvaz	NS	CS	Conv	Pregnant women	80	0.00
Mousavi, 2010^173^	2008	Khuzestan	Clinical setting (hospital)	CS	Conv	Renal transplant donors	79	0.00
Mousavi, 2011^174^	2009–2010	Ahvaz	Clinical setting (hospital)	CS	Conv	Renal transplant donors	52	0.00
Pourshams, 2005^175^	2001	Tehran	Blood bank	CS	SRS	Blood donors	1,959	0.46
Poustchi, 2011^56^	NS	Golestan	Community	CS	Cluster sampling	General population	49,338	0.50
Rahbar, 2004^176^	2001–2002	Mashhad	Blood transfusion center	CS	NS	Blood donors	60,892	0.10
Rahnama, 2005^177^	2000–2001	Kerman	Regional blood transfusion center	CC	SRS	Blood donors	140	2.1
Razjou, 2012^178^	2009	National	Blood bank	CS	Conv	Blood donors	1,494,282	0.13
Rezaie, 2016^179^	2011–2015	Semnan	Blood transfusion center	CS	Conv	Blood donors	42,253	0.06
Rezazadeh, 2006^180^	2004–2005	Hamadan	Blood transfusion center	CS	Conv	Blood donors	18,306	0.43
Roshan, 2012^181^	2007–2008	Ahvaz	Fertility clinic/IVF	CS	Conv	Infertile couples (male)	712	0.84
Roshan, 2012^181^	2007–2008	Ahvaz	Fertility clinic/IVF	CS	Conv	Infertile couple (female)	712	0.42
Salehi, 2011^182^	2002–2006	Isfahan	Clinical: hospital & health care centers	CS	Conv	Blood donors	4,808	0.27
Samadi, 2014^183^	2012	Ahvaz	Blood transfusion center	CS	Conv	Blood donors	2,108	0.00
Seyed-Askari, 2015^184^	2009–2013	Kerman	Blood transfusion center	CS	Conv	Blood donors	360,722	0.08
Shaheli, 2015^185^	2012	Shiraz	Community	CC	Conv	Healthy adults	100	0
Shahshahani, 2013^186^	2004–2010	Yazd	Blood transfusion center	CS	Conv	Blood donors	346,471	0.07
Shakeri, 2013^187^	2010–2011	Mashhad	Community	CS	Cluster sampling	General population	3,870	0.13
Shamsdin, 2012^188^	2010–2011	Shiraz	Community	CS	Conv	General population	2,080	0.72
Sofian, 2010^189^	2008	Arak	Regional blood transfusion center	CS	SRS	Blood donors	531	0.19
Sohrabpour, 2010^190^	NS	Mixed (Hormozgan, Tehran, Golestan)	Community	CS	Cluster sampling	General population	5,589	0.88
Tahereh, 2005^191^	2000–2002	Ghazvin	Blood transfusion center	CS	SRS	Blood donors	39,598	0.25
Taheri, 2008^192^	2003–2005	Rasht	Blood transfusion center	CS	Conv	Blood donors	49,820	0.18
Tajbakhsh, 2007^193^	2004	Shahrekord	Blood transfusion center	CS	Conv	Blood donors	11,472	0.60
Tanomand, 2007^194^	2005	Malekan city	Clinical setting (hospital)	CS	SRS	General population	346	0.29
Vahidi, 2000^195^	1996	Kerman	Clinical setting (hospital)	CC	Conv	Healthy children	107	0.00
Yazdani, 2006^196^	1998–2000	Kermanshah	Clinical setting (hospital)	CS	Conv	Pregnant women	2,000	0.60
Zamani, 2013^197^	2008–2011	Mazandaran	Primary health care centers (community)	CS	Cluster sampling	General population	6,145	0.08
Zanjani, 2013^198^	2005–2006	Zanjan	Blood transfusion center	CS	Conv	Blood donors	29716	0.11

^a^Abbreviations: CC, case-control; Conv, convenience; CS, cross-sectional; EMRO, Eastern Mediterranean Regional Office (WHO); IVF, *in vitro* fertilization; NS, not specified, SRS; simple random sampling.

^b^The decimal places of the prevalence figures are as reported in the original reports, but prevalence figures with more than one decimal places were rounded to one decimal place, with the exception of those below 1%.

#### Populations at high risk

A total of 208 HCV prevalence measures were identified in populations at high risk (Table 3), ranging from 0.0% to 90.0%, with a median of 26.3%. The majority were conducted on high risk clinical populations (n = 127). In hemophilia patients (n = 25), HCV prevalence ranged from 6.0% to 90.0%, with a median of 54.0%. In thalassemia patients (n = 58), HCV prevalence ranged from 0.0% to 68.9%, with a median of 16.6%. In hemodialysis patients (n = 41), HCV prevalence ranged from 0.0% to 31.4%, with a median of 8.3%. In HIV positive patients (n = 25), HCV prevalence ranged from 3.9% to 89.3%, with a median of 67.7%. Among PWID (n = 56), HCV prevalence ranged from 11.3% to 88.9%, with a median of 51.4%.

**Table 3:** Studies reporting hepatitis C virus (HCV) prevalence among populations at high risk in Iran.

Author, year (citation)	Year(s) of data collection	City or country of survey	Study site	Study design	Study sampling procedure	Population	Sample size	HCV prevalence (%)
Abdollahi, 2008^199^	2003	NS	Hemophilia units	CS	Conv	Hemophilia patients	174	83.3
Aghakhani, 2009^200^	NS	Tehran	NS	CS	Conv	HIV patients	106	67.0
Aghakhani, 2009^200^	NS	Tehran	NS	CS	Conv	Hemodialysis patients	289	3.1
Akbari, 2011^201^	2003–2004	Shiraz	Thalassemia center	CC	SRS	Thalassemia patients	200	25.0
Alavi, 2005^202^	2002	Tehran	Clinical: hospital & health care centers	CS	Conv	Thalassemia patients	110	11.8
Alavi, 2007^203^	2001–2003	Ahvaz	Clinical: hospital & health care centers	CS	Conv	PWID with HIV	104	74.0
Alavi, 2009^204^	2001–2006	Ahvaz	Clinical: hospital & health care centers	CS	Conv	PWID	142	52.1
Alavi, 2012^110^	NS	Tehran	Clinical: hospital & health care centers	CC	Conv	Thalassemia patients (<18)	90	13.3
Alavia, 2003^205^	NS	Ghazvin	Clinical: hospital & health care centers	NS	NS	Thalassemia patients	95	24.2
Alavian, 2003^206^	2000–2001	NS	Hemophilia units	CS	Conv	Hemophilia patients	176	60.2
Alavian, 2008^94^	1999	National	NS	NS	NS	Hemodialysis patients	NS	14.4
Alavian, 2008^94^	2000	National	NS	NS	NS	Hemodialysis patients	NS	11.2
Alavian, 2008^94^	2001	National	NS	NS	NS	Hemodialysis patients	NS	8.8
Alavian, 2008^94^	2002	National	NS	NS	NS	Hemodialysis patients	NS	8.2
Alavian, 2008^94^	2003	National	NS	NS	NS	Hemodialysis patients	NS	6.7
Alavian, 2008^94^	2004	National	NS	NS	NS	Hemodialysis patients	NS	5.6
Alavian, 2008^94^	2005	National	NS	NS	NS	Hemodialysis patients	NS	4.8
Alavian, 2008^94^	2006	National	NS	NS	NS	Hemodialysis patients	NS	4.5
Alavian, 2015^112^	2012	Isfahan	Hemodialysis units	CC	Conv	Hemodialysis units	274	0
Alipour, 2013^61^	2003–2011	Shiraz	Counseling centers	CS	Conv	HIV patients (male)	215	17.7
Alipour, 2013^61^	2003–2011	Shiraz	Counseling centers	CS	Conv	HIV patients (female)	1,230	89.1
Alipour, 2013^207^	NS	Mixed (Shiraz, Tehran, Mashhad)	Drop in centers and rehab centers	CS	Conv	PWID (male)	226	38.6
Alipour, 2013^67^	2011	Shiraz	Counseling centers	CS	SRS	HIV patients	168	87.5
Alizadeh, 2005^208^	2002	Hamedan	Prison	CS	SRS	PWID	149	31.5
Alizadeh, 2006^209^	NS	Hamadan	Clinical: hospital & health care centers	CS	NS	Hemophilia patients	66	59.1
Ameli, 2008^210^	2006	Mazandaran	Clinical: hospital & health care centers	CS	Conv	Thalassemia patients	65	16.9
Amin-Esmaeili, 2012^57^	2006–2007	Tehran	Drop in centers and rehab centers	CS	Conv	PWID	895	34.5
Amiri, 2005^211^	2001	Guilan	Hemodialysis units	CS	Conv	Hemodialysis patients	298	24.8
Ansar, 2002^114^	1997–1998	Rasht	Clinical: hospital & health care centers	CS	SRS	Thalassemia patients (female)	50	62.0
Ansar, 2002^114^	1997–1998	Rasht	Clinical: hospital & health care centers	CS	SRS	Thalassemia patients (male)	55	65.0
Ansar, 2002^114^	1997–1998	Rasht	Clinical: hospital & health care centers	CS	SRS	Hemophilia patients	93	55.9
Ansari, 2007^212^	2005–2006	Shiraz	Clinical: hospital & health care centers	CS	Conv	Thalassemia patients (female)	400	16.0
Ansari, 2007^212^	2005–2006	Shiraz	Clinical: hospital & health care centers	CS	Conv	Thalassemia patients (male)	406	12.8
Asl, 2013^213^	2003–2005	Alborz	Prisons	Coh	Conv	PWID	150	69.3
Assarehzadegan, 2012^214^	2008–2009	Ahvaz	Clinical: hospital & health care centers	CS	Conv	Hemophilia patients	87	54.0
Ataei, 2010^215^	2008–2009	Isfahan	Drop in centers and rehab centers	CS	Conv	PWID	3,284	38.0
Ataei, 2010^53^	1998–2007	Isfahan	Clinical: hospital & health care centers	CS	Conv	HIV patients	130	77.0
Ataei, 2011^216^	NS	Isfahan	Prison, drop in centers and rehab centers	CS	Conv	PWID	1,485	43.4
Ataei, 2011^217^	NS	Isfahan	Drop in centers and rehab centers	CS	Conv	PWID	136	19.8
Ataei, 2012^218^	1996–2011	Isfahan	Clinical: hospital & health care centers	CS	Conv	Thalassemia patients	463	8.0
Azarkeivan, 2010^219^	1996–2005	Tehran	Thalassemia center	CS	Conv	Thalassemia patients	395	27.5
Azarkeivan, 2011^220^	2008	Tehran	Thalassemia center	CS	Conv	Thalassemia patients	695	24.5
Azarkeivan, 2012^45^	1996–2009	Tehran	Clinical: hospital & health care centers	Coh	Conv	Thalassemia patients	395	7.6
Babamahmoodi, 2012^221^	2008–2010	Mazandaran	Clinical: hospital & health care centers	CS	Conv	HIV patients	80	58.8
Basiratnia, 2010^222^	1999	Shahrekord	Clinical: hospital & health care centers	NS	NS	Thalassemia patients (female)	50	22.0
Basiratnia, 2010^222^	1999	Shahrekord	Clinical: hospital & health care centers	NS	NS	Thalassemia patients (male)	63	23.8
Boroujerdnia, 2009^223^	2006–2007	Khuzestan	Clinical: hospital & health care centers	CS	Conv	Thalassemia patients	206	28.1
Bozorghi, 2006^224^	2004	Ghazvin	Clinical: hospital & health care centers	CS	Conv	Hemodialysis patients	89	6.7
Bozorgi, 2008^225^	2005	Ghazvin	Clinical: hospital & health care centers	CS	Conv	Thalassemia patients	207	24.2
Broumand, 2002^226^	NS	Tehran	Hemodialysis units	CS	Conv	Hemodialysis patients	548	19.6
Company, 2007^227^	2005–2006	Ahvaz	Clinical: hospital & health care centers	CS	Conv	Thalassemia patients	195	20.5
Dadgaran, 2005^228^	NS	Guilan	Hemodialysis units	NS	NS	Hemodialysis patients	393	17.8
Dadmanesh, 2015^229^	2012–2013	Tehran	Clinical: hospital & health care centers	CS	Conv	Hemodialysis patients	138	0
Davarpanah, 2013^230^	2006–2007	Shiraz	Counseling centers	CS	Conv	HIV patients	226	86.7
Davoodian, 2009^231^	2002	Tehran	Prisons	CS	SRS	PWID	249	64.8
Eghbalian, 2000^232^	NS	Hamedan	Clinical: hospital & health care centers	CS	NS	Thalassemia patients (<15)	53	34.0
Esfahani, 2014^233^	2012	Hamedan	Hemophilia units	CS	Conv	Hemophilia patients	89	49.4
Eskandarieh, 2013^234^	NS	Tehran	Drop in centers and rehab centers	CS	Conv	PWID	258	65.9
Eslamifar, 2007^235^	2006	Tehran	Hemodialysis units	CS	Conv	Hemodialysis patients	77	6.5
Etminani-Esfahani, 2012^236^	NS	Tehran	Clinical: hospital & health care centers	CS	Conv	HIV patients	98	55.1
Faramarzi, 2013^237^	2010	Shiraz	Voluntary counseling center	CS	Conv	HIV patients (male)	222	64.0
Faranoush, 2006^238^	2002	Mixed (Semnan, Damaghan, Garmsar)	Clinical: hospital & health care centers	CS	Conv	Thalassemia patients	630	39.7
Farhoudi, 2016^239^	2013–2014	Tehran	Prison	CS	Conv	HIV patients	56	89.3
Ghaderi, 1996^240^	NS	Fars	Blood transfusion center	CS	Conv	Thalassemia patients	90	68.5
Ghadir, 2009^241^	2008	Qom	Hemodialysis units	CS	Conv	Hemodialysis patients	90	21.1
Ghafoorian-Broujerdnia, 2006^242^	1999–2004	Ahvaz	Clinical: hospital & health care centers	CS	Conv	Thalassemia patients	122	26.2
Ghane, 2012^243^	2010	Mixed (Mazandaran and Guilan)	Clinical: hospital & health care centers	CS	Conv	Thalassemia patients	245	14.7
Haghazali, 2011^244^	2007	Ghazvin	Clinical: hospital & health care centers	CS	Conv	Hemodialysis patients (males)	76	7.5
Hamissi, 2011^245^	2009	Ghazvin	Clinical: hospital & health care centers	CS	Conv	Hemodialysis patients	195	6.7
Hariri, 2006^246^	2004	Isfahan	Clinical: hospital & health care centers	CS	Conv	Hemophilia patients	120	64.0
Hariri, 2006^246^	2004	Isfahan	Clinical: hospital & health care centers	CS	Conv	Thalassemia patients	616	10.9
Honarvar, 2013^247^	2012–2013	Shiraz	Drop in centers and rehab centers	CS	Conv	PWID	233	40.3
Hosseini, 2010^63^	2006	Tehran	Prison	CS	Conv	PWID	417	80.0
Imani, 2008^248^	2004	Shahr-e-Kord	Drop in centers and rehab centers	CS	Conv	PWID	133	11.3
Ismail, 2005^249^	NS	Tehran	Clinical: hospital & health care centers	CS	Conv	PWID	65	17.0
Jabbari, 2008^46^	2005–2006	Golestan	Clinical: hospital & health care centers	CS	Conv	Hemodialysis patients	93	24.7
Joukar, 2011^250^	2009	Guilan	Hemodialysis units	CS	Conv	Hemodialysis patients	514	11.9
Kaffashian, 2011^251^	NS	Isfahan	Prison	CS	Conv	PWID	951	42.0
Kalantari, 2011^252^	2008–2010	Isfahan	Clinical: hospital & health care centers	CS	Conv	Thalassemia patients	545	9.1
Kalantari, 2011^252^	2008–2010	Isfahan	Clinical: hospital & health care centers	CS	Conv	Hemophilia patients	615	80.5
Kalantari, 2014^253^	2010–2011	Isfahan	Hemodialysis units	CS	Cluster sampling	Hemodialysis patients	499	5.2
Karimi, 2001^254^	1999–2001	Shiraz	Thalassemia center	CS	Conv	Thalassemia patients	466	15.7
Karimi, 2001^255^	1999–2001	Shiraz	Hemophilia unit	CS	Conv	Hemophilia patients	281	15.7
Karimi, 2002^256^	2002	Shiraz	Clinical: hospital & health care centers	CS	Conv	Coagulation disorder patients	367	13.1
Kashef, 2008^257^	NS	Tabriz	Clinical: hospital & health care centers	CS	Conv	Thalassemia patients	131	18.3
Kassaian, 2011^258^	2009	Isfahan	Thalassemia center	CS	Conv	Thalassemia patients	570	10.5
Kassaian, 2011^258^	2009	Isfahan	Hemodialysis unit	CS	Conv	Hemodialysis patients	800	2.1
Kassaian, 2012^58^	2009	Isfahan	Prison	CS	Conv	PWID	943	41.6
Keramat, 2011^259^	2005–2007	Hamadan	Counseling center	CS	Conv	PWID	199	63.3
Keshvari, 2014^260^	2008–2010	Tehran	Thalassemia center	CS	Conv	Thalassemia patients	257	40.1
Khani, 2003^261^	2001	Zanjan	Prison	CS	Conv	PWID	346	50.9
Kheirandish, 2009^52^	2006	Tehran	Prison	CS	Conv	PWID	454	80.0
Khorvash, 2008^262^	2005	Isfahan	Clinical: hospital & health care centers	CS	Conv	PWID	92	74.3
Khosravi, 2010^263^	NS	Shiraz	Counseling center	CS	Conv	HIV patients	101	86.1
Kiakalayeh, 2013^264^	2002–2011	Rasht	Clinical: hospital & health care centers	CS	Conv	Thalassemia patients	1,113	10.5
Lak, 2000^265^	NS	Tehran	Hemophilia units	CS	Conv	Hemophilia patients	100	90.0
Lak, 2000^265^	NS	Tehran	Clinical: hospital & health care centers	CS	Conv	VWD patient	385	55.1
Langarodi, 2011^266^	2009–2010	Karaj	Clinical: hospital & health care centers	CS	Conv	Thalassemia patients	206	14.1
Mahdaviani, 2008^267^	2004	Markazi	Clinical: hospital & health care centers	CS	Conv	Hemophilia patients	68	36.7
Mahdaviani, 2008^267^	2004	Markazi	Clinical: hospital & health care centers	CS	Conv	Thalassemia patients	97	7.2
Mahdavimazdeh, 2009^268^	2005	Tehran	Hemodialysis units	CS	Conv	Hemodialysis patients	2,403	9.5
Mak, 2001^269^	NS	Isfahan	Hemodialysis units	CS	Conv	Hemodialysis patients	86	31.1
Makhlough, 2008^270^	2006	Sari and Ghaemshahr, Mazandaran	Hemodialysis units	CS	Conv	Hemodialysis patients	186	11.3
Mansour-Ghanaei,2002^271^	1999	Guilan	Hemophilia units	CS	Conv	Hemophilia patients	101	71.3
Mansour-Ghanaei,2009^272^	2007	Rasht	Clinical: hospital & health care centers	CS	Conv	Hemodialysis patients	163	10.4
Mansour-Ghanaei,2009^273^	NS	Guilan	Thalassemia center	CS	Conv	Thalassemia patients	370	50.4
Mashayekhi, 2011^274^	2008–2009	Tabriz	Thalassemia center	CS	Conv	Thalassemia patients	100	3.0
Mehrjerdi, 2014^68^	2011	Tehran	Drop in centers and rehab centers	CS	Conv	PWID	209	26.8
Meidani, 2009^275^	2007–2008	Isfahan	Clinical: hospital & health care centers	CS	Conv	PWID	150	26.0
Mirahmadizadeh, 2004^276^	NS	Shiraz	NS	CS	NS	PWID	186	80.1
Mirahmadizadeh, 2009^277^	NS	National	Drop in centers and rehab centers	CS	SRS	PWID	1,531	43.4
Mirmomen, 2006^278^	2002	Mixed (Tehran, Kerman, Ghazvin, Semnan, Zanjan)	Blood transfusion centers	CS	Conv	Thalassemia patients	732	19.6
Mir-Nasseri, 2005^62^	2001–2002	Tehran	Drop in centers and rehab centers	CS	NS	PWID	467	66.0
Mir-Nasseri, 2011^55^	2001–2002	Tehran	Prison, drop in centers and rehab centers	CS	Conv	PWID	518	69.3
Mobini, 2010^279^	2006	Yazd	Clinical: hospital & health care centers	CS	Conv	Hemophilia patients	77	49.4
Mohammadi, 2009^280^	2007–2008	Lorestan	NS	CS	Conv	HIV patients	391	72.1
Momen-Heravi, 2012^281^	NS	Kashan	Drop in centers and rehab centers	CS	Cluster sampling	PWID	300	47.3
Mousavi, 2002^282^	NS	NS	NS	NS	NS	Thalassemia patients	81	27.2
Mousavian, 2011^283^	2003–2005	Tehran	Hemophilia units	CS	Conv	Hemophilia patients	1,095	72.3
Naini, 2007^284^	1993–2006	Isfahan	Hemophilia units	CS	Conv	Hemophilia patients	553	22.6
Najafi, 2001^285^	1998	Qaemshahr	Thalassemia centers	CS	Conv	Thalassemia patients	100	18.0
Rahbar, 2004^176^	2001	Mashhad	Prison	CC	Conv	PWID	101	59.4
Rahimi-Movaghar, 2010^286^	2006–2007	Tehran	Drop in centers and rehab centers	CS	Snowball sampling	PWID	899	34.5
Ramezani, 2008^287^	2005–2006	Tehran	Counseling center	CS	Conv	HIV patients	171	52.6
Ramezani, 2009^288^	NS	Tehran	Clinical: hospital & health care centers	CS	Conv	HIV patients	91	68.5
Ramzani, 2014^289^	2012	Arak	Drop in centers and rehab centers	CS	Conv	PWID	100	56.0
Rostami, 2013^290^	2010–2011	Mixed	Hemodialysis units	CS	Conv	Hemodialysis patients	3963	1.3
Rostami-Jalilian, 2006^291^	2002–2004	Isfahan	Clinical: hospital & health care centers	CS	Conv	PWID with thrombosis	72	45.8
Rostami-Jalilian, 2006^291^	2002–2004	Isfahan	Clinical: hospital & health care centers	CS	Conv	PWID without thrombosis	76	34.2
Sabour, 2003^292^	1999–2000	Kermanshah	Clinical: hospital & health care centers	CS	Conv	Hemodialysis patients	140	26.4
Saleh, 2011^293^	2007–2008	Hamedan	Clinical: hospital & health care centers	CC	Conv	PWID (corpses)	94	60.6
Salehi, 2015^69^	2006–2011	Shiraz	Drop in centers and rehab centers	CS	Conv	PWID	1,327	13.5
Sali, 2013^294^	2010–2012	Tehran	Clinical: hospital & health care centers	CS	Conv	HIV patients	200	71.0
Samak, 2012^295^	2007	Qom	Clinical: hospital & health care centers	CS	Conv	Thalassemia patients	142	13.4
Samarbaf-Zadeh, 2015^296^	NS	Khuzestan	Clinical: hospital & health care centers	CS	Conv	Hemodialysis patients	430	9.1
Samimi-Rad, 2007^297^	2004	Markazi	Clinical: hospital & health care centers	CS	Conv	Thalassemia patients (male)	50	4.0
Samimi-Rad, 2007^297^	2004	Markazi	Clinical: hospital & health care centers	CS	Conv	Patients with Inherited bleeding disorder	76	43.4
Samimi-Rad, 2007^298^	2005	Isfahan	Clinical: hospital & health care centers	CS	Conv	Hemophilia patients	50	100.0
Samimi-Rad, 2007^298^	2005	Isfahan	Clinical: hospital & health care centers	CS	Conv	Thalassemia patients	53	100.0
Samimi-Rad, 2008^299^	2005	Markazi	Hemodialysis units	CS	Conv	Hemodialysis patients	204	4.9
Sanei, 2004^300^	2002	Zahedan	Clinical: hospital & health care centers	CS	Conv	Thalassemia patients	364	13.5
Sani, 2012^301^	2007–2009	Mashhad	Clinical: hospital & health care centers	CS	Conv	PWID	62	71.0
Sarkari, 2012^59^	2009–2010	Mixed (Kohgiloyeh and Boyerahmad)	NS	CS	Conv	PWID	158	42.2
SeyedAlinaghi, 2011^302^	2004–2005	Tehran	Clinical: hospital & health care centers	CS	Conv	HIV patients	201	67.2
Seyrafian, 2006^303^	2005	Isfahan	Hemodialysis units	CS	Conv	Hemodialysis patients	556	2.9
Shahshahani, 2006^304^	NS	Yazd	NS	CS	NS	Hemophilia patients	74	48.6
Shahshahani, 2006^304^	NS	Yazd	NS	CS	NS	Thalassemia patients	85	9.4
Sharif, 2009^305^	2001–2006	Kashan	Clinical: hospital & health care centers	CS	Conv	PWID	200	12.0
Sharifi-Mood, 2006^306^	1986–2005	Zahedan	Hemophilia units	CS	Conv	Hemophilia patients	74	31.1
Sharifi-Mood, 2007^307^	2003–2006	Zahedan	Hemophilia units	CS	Conv	Hemophilia patients	81	29.6
Siavash, 2008^308^	2007	Kermanshah	Clinical: hospital & health care centers	CS	Conv	HIV patients	888	3.9
Sofian, 2012^309^	2009	Markazi	Prison	CS	Conv	PWID	153	59.5
Somi, 2007^310^	2006	Tabriz	Hemodialysis units	CS	Conv	Hemodialysis patients	462	14.9
Somi, 2014^311^	2012	Tabriz	Hemodialysis units	CS	Conv	Hemodialysis patients	455	8.1
Taremi, 2005^312^	2004	Tabriz	Hemodialysis units	CS	Conv	Hemodialysis patients	324	20.4
Tayeri, 2008^313^	2000–2007	Isfahan	Clinical: hospital & health care centers	CS	Conv	PWID with HIV	106	75.5
Taziki, 2008^314^	2001	Mazandaran	Hemodialysis units	CS	Conv	Hemodialysis patients	348	18.0
Taziki, 2008^314^	2006	Mazandaran	Hemodialysis units	CS	Conv	Hemodialysis patients	497	12.0
Toosi, 2007^315^	NS	Tehran	Clinical: hospital & health care centers	CS	Conv	Hemodialysis patients	130	8.5
Torabi, 2005^316^	2003	Azerbaijan	Clinical: hospital & health care centers	CS	Conv	Thalassemia patients (<18)	84	7.1
Torabi, 2006^317^	2003	Azerbaijan	Clinical: hospital & health care centers	CS	Conv	Hemophilia patients	130	55.4
Vahidi, 2000^195^	1996	Kerman	Clinical: hospital & health care centers	CC	Conv	Thalassemia patients	107	22.4
Valizadeh, 2013^318^	2010	Urmia	Hemophilia units	CS	Conv	Hemophilia patients	50	6.0
Yazdani, 2012^319^	1996–2010	Isfahan	Hemophilia units	CS	Conv	Hemophilia patients	350	66.0
Zadeh, 2007^320^	2007	Tehran	NS	CS	Conv	PWID (males)	70	36.0
Zahedi, 2004^321^	2002	Kerman	Clinical: hospital & health care centers	CS	Conv	Hemophilia patients	97	44.3
Zahedi, 2012^322^	2010	Kerman	Hemodialysis centers	CS	Conv	Hemodialysis patients	228	3.0
Zali, 2001^323^	1995	Tehran	Prison	CS	SRS	PWID (male)	402	45.0
Zamani, 2007^51^	2004	Tehran	Community, drop in centers and rehab centers	CS	Conv	PWID	202	52.0
Zamani, 2010^64^	2008	Foulad-Shahr City	Drop in centers and rehab centers	CS	Snowball sampling	PWID	117	60.7
Ziaee, 2005^324^	2000	Khorasan	Drop in centers and rehab centers	CS	Conv	Hemophilia patients	80	55.0
Ziaee, 2007^325^	NS	South Khorassan	Hemophilia units	CS	Conv	Hemophilia patients	80	26.3
Ziaee, 2015^326^	2010–2012	Birjand	Hemophilia units	CS	Conv	Hemophilia patients	108	20.4

^a^Abbreviations: CC, case-control; Coh, cohort; Conv, convenience; CS, cross-sectional; NS, not specified; PWID, people who inject drugs; SRS, simple random sampling; VWD, von Willebrand disease.

^b^The decimal places of the prevalence figures are as reported in the original reports, but prevalence figures with more than one decimal places were rounded to one decimal place, with the exception of those below 1%.

#### Populations at intermediate risk

A total of 70 HCV prevalence measures were identified in intermediate risk populations (Table S2), ranging from 0.0% to 48.0%, with a median of 3.3%. In prisoners (n = 15), HCV prevalence ranged from 0.7% to 37.9%, with a median of 4.1%. In homeless people (n = 10), HCV prevalence ranged from 0.0% to 48.0%, with a median of 3.0%. Half of these studies were conducted on homeless children, among which HCV prevalence ranged from 0.0% to 3.5%, with a median of 1.0%. In household contacts of HCV index patients (n = 5), HCV prevalence ranged from 0.0% to 3.3%, with a median of 2.2%. In healthcare workers (n = 11), HCV prevalence ranged from 0.0% to 37.0%, with a median of 0.0%. In drug users (where the route of drug use was not specified (n = 13), HCV prevalence ranged from 3.4% to 36.1%, with a median of 14.5%.

#### Special clinical populations

A total of 72 HCV prevalence measures were identified in special clinical populations (Table S3), ranging from 0.0% to 69.1%, with a median of 3.2%. In hepatitis B virus patients, prevalence ranged from 0.0% to 18.0%, with a median of 10.3%. In viral hepatitis patients (n = 9), HCV prevalence ranged from 0.0% to 34.9%, with a median of 6.1%. In patients with liver cirrhosis (n = 5), HCV prevalence ranged from 1.7% to 14.9%, with a median of 7.3%.

### Pooled mean HCV prevalence estimates

Table 4 shows the results of our meta-analyses for HCV prevalence. The estimated national population-level HCV prevalence, based on the pooled HCV prevalence in the general population, was 0.3% (95% CI: 0.2–0.4%). There was significant evidence of heterogeneity (p < 0.0001). I^2^ was estimated at 99.8% (95% CI: 99.8–99.8%), indicating that almost all observed variation is attributed to true variation in HCV prevalence rather than sampling error. The prediction interval was 0.0–1.5%.

**Table 4:** Results of the meta-analyses for hepatitis C virus (HCV) prevalence measures in Iran stratified by populations’ risk of exposure.

Population at risk	Studies	Samples	HCV prevalence				Heterogeneity measures		
	Total N	Total n	Range (%)	Mean (%)	95% CI		Q (p-value)ª	I² (confidence limits)^c^	Prediction interval (%)^d^
General population (populations at low risk)	122	16,073,479	0.0–3.1	0.3	0.2–0.4		56269.6 (p < 0.0001)	99.8% (99.8–99.8%)	0.0–1.5
Populations at high risk	208	55,257	0.0–90.0	32.1	28.1–36.2		217272.1 (p < 0.0001)	99.0% (99.0–99.1%)	0.0–88.5
PWID	56	17,999	11.3–88.9	52.2	46.9–57.5		2615 (p < 0.0001)	97.9% (97.6–98.1%)	15.8–87.3
Populations at high risk of healthcare-related exposures	127	32,517	0.0–90.0	20.0	16.4–23.9		8786.2 (p < 0.0001)	98.6% (98.5–98.8%)	0.0–69.7
Populations at intermediate risk	70	36,879	0.0–48.0	6.2	3.4–9.6		9,128 (p < 0.0001)	99.2% (99.2–99.3%)	0.0–49.9
Special clinical populations	72	55,187	0.0–69.1	4.6	3.2–6.1		2293.6 (p < 0.0001)	96.9% (96.5–97.3%)	0.0–21.6
Populations with liver-related conditions	28	6,338	0.0–34.9	7.5	4.3–11.4		639.5 (p < 0.0001)	95.8% (94.8–96.6%)	0.0–35.2
Other special clinical populations	44	48,849	0.0–69.1	2.7	1.8–3.6		520.6 (p < 0.0001)	91.7% (89.8–93.3%)	0.0–9.6

^a^Abbreviations: CI, confidence interval.

^b^Q: Cochran Q statistic assessing the existence of heterogeneity in HCV prevalence estimates.

^c^I²: a measure assessing the magnitude of between-study variation that is due to difference in HCV prevalence estimates across studies rather than chance.

^d^Prediction interval: a measure estimating the 95% interval in which the true HCV prevalence in a new HCV study will lie.

The pooled mean HCV prevalence for populations at high risk was 32.1% (96% CI: 28.1–36.2%). There was significant evidence of heterogeneity (p < 0.0001), with an I^2^ of 99.0% (95% CI: 99.0–99.1%). The prediction interval was 0.0–88.5%. For the subpopulations of PWID and populations at high risk of healthcare-related exposures, the pooled means were 52.2% and 20.0%, respectively.

The pooled mean HCV prevalence for populations at intermediate risk was 6.2% (95% CI: 3.4–9.6%). There was significant evidence of heterogeneity (p < 0.0001), with an I^2^ of 99.2% (95% CI: 99.2–99.3%). The prediction interval was 0.0–49.9%.

The pooled mean HCV prevalence for special clinical populations was 4.6% (95% CI: 3.2–6.1%). There was significant evidence of heterogeneity (p < 0.0001), with an I^2^ of 96.9% (95% CI: 96.5–97.3%). The prediction interval was 0.0–21.6%. For the subpopulations of populations with liver-related conditions and other special clinical populations, the pooled means were 7.5% and 2.7%, respectively.

The forest plots for the HCV prevalence meta-analyses can be found in Figs S3 and S4.

### Sensitivity analysis

After excluding blood donor data, the national population-level HCV prevalence was estimated at 0.3% (95% CI: 0.2–0.5%). There was significant evidence of heterogeneity (p < 0.0001), with an I^2^ of 76.3% (95% CI: 67.5–81.7%). The prediction interval was 0.0–1.3%. The forest plot for this sensitivity analysis can be found in Fig. S5.

### HCV RNA prevalence

Our search identified a total of 55 HCV RNA measures. The details of each of these measures can be found in Table S4. These were reported either among HCV antibody positive individuals, or as a proportion of the entire sample. HCV RNA prevalence among HCV antibody positive individuals ranged from 0% to 89.3%, with a median of 61.9%. HCV RNA prevalence as a proportion of the entire sample ranged from 0% to 60.0%, with a median of 8.6%. HCV RNA prevalence as a proportion of the entire sample was high in several populations at high risk of healthcare-related exposures.

### Risk factors for HCV infection

A number of studies assessed risk factors for HCV exposure using multivariable regression analyses. Risk factors most commonly reported included history and duration of incarceration and multiple incarcerations^50–62^, history and duration of intravenous drug use^50,51,54,57,58,60–67^, history of sharing a needle or syringe^55,57,62,68,69^, history of tattooing^50–52,61,70,71^, history of sharing razors^67^, multiple sex partners^57,58,66,67,69,70,72^, being a man who have sex with men^54,62,68,73^, history of surgery^70,73^, history of blood transfusion^56,60,73^, and history of hemodialysis^74^.

### HCV genotypes

HCV genotype data was identified in 66 studies including a total of 24,029 HCV RNA positive individuals. Of these, 895 individuals had an undetermined genotype and were therefore excluded from further analysis. The vast majority of individuals were infected by a single genotype, with only 2.9% being infected by multiple genotypes. The proportion of infections for each HCV genotype was highest in genotype 1 (58.2%), followed by genotype 3 (39.0%), genotypes 2 (1.7%), and genotype 4 (1.0%).

The pooled mean proportion for genotype 1 was 56.3% (95% CI: 52.9–59.6%), genotype 3 was 38.8% (95% CI: 35.7–41.9%), genotype 2 was 0.4% (95% CI: 0.0–1.0%), and genotype 4 was 0.0% (95% CI: 0.0–0.1%).

Genotype 1 was more common among populations at high risk of healthcare-related exposures than genotype 3. Meanwhile, genotype 3 was more common among PWID than genotype 1. Within genotype 1, subtype 1a and subtype 1b were isolated (where subtype information was available) from 79.5% and 20.5% of individuals, respectively.

### Quality assessment

The results of the quality assessment are summarized in Table 5. The majority of HCV incidence measures (60%) were based on samples with >100 participants, and therefore were classified as having high precision. Incidence studies were based on convenience sampling from clinical facilities, and 60% had a response rate >80%. All incidence measures were based on biological assays.

**Table 5:** Summary of precision and risk of bias (ROB) assessment for the hepatitis C virus (HCV) incidence and prevalence measures extracted from eligible reports.

Quality assessment	HCV incidence		HCV prevalence	
	n	%	n	%
**Precision of estimates**				
High precision	3	60.0	312	77.4
Low precision	2	40.0	79	19.6
Unclear^a^			12	3.0
**Risk of bias quality domains**				
**HCV ascertainment**				
Low risk of bias	5	100	402	100
High risk of bias	0	0	0	0
**Sampling methodology**				
Low risk of bias	0	0	48	11.9
High risk of bias	5	100	350	87.1
Unclear	0	0	4	1.0
**Response rate**				
Low risk of bias	3	60.0	370	92.0
High risk of bias	2	40.0	10	2.5
Unclear^a^	0	0	22	5.5
**Total studies where risk of bias assessment was possible**	**5**	**100**	**402**	**99.8**
**Unknown** ^b^	0	0		0.2
**Total studies**	**5**	**100**	**403**	**100**
**Summary of risk of bias assessment for HCV prevalence measures**	**n**	**%**		
**Low risk of bias**				
In at least one quality domain	402	100		
In at least two quality domains	371	92.3		
In all three quality domains	47	11.7		
**High risk of bias**				
In at least one quality domain	350	87.1		
In at least two quality domains	10	2.5		
In all three quality domains	0	0		
**Total studies where risk of bias assessment was possible**	**402**	**99.8**		
**Total studies**	**403**	**100**		

^a^Studies with missing information for any of the domains were classified as having unclear risk of bias for that specific domain.

^b^Studies extracted through country-level routine reporting with limited description of the sample (not permitting the conduct of risk of bias assessment) were classified as being of unknown quality.

The majority of HCV prevalence measures (77.4%) were based on samples with >100 participants, and therefore were classified as having high precision. Of the 403 prevalence measures, ROB assessment was possible for 402 measures.

All HCV prevalence measures were based on biological assays. In 25.0% of measures, information on the exact biological assay was missing. Approximately one third of the samples underwent secondary confirmatory testing, with the majority using the more sensitive and specific recombinant immunoblot assay (RIBA). Among studies where information was available on assay generation, the majority (71.2%) used the more recent, sensitive, and specific 3rd generation Enzyme-linked immunosorbent assay (ELISA) tests, and 26.9% used 2^nd^ generation ELISA. The majority of samples (82.6%) were drawn using non-probability based sampling. Response rate was high in 92.0% of studies.

In summary, HCV prevalence measures were of reasonable quality. All studies had a low ROB in at least one quality domain, 92.3% had a low ROB in at least two of the three quality domains, and 11.7% had a low ROB in all three quality domains. Only 2.5% of studies had a high ROB in two of the three quality domains, and no study had a high ROB in all three quality domains.

### Meta-regressions and sources of heterogeneity

The results of our meta-regression models can be found in Table 6. The univariable meta-regression analyses identified population, study site, sample size, and year of data collection as significant predictors (with p < 0.1), and therefore eligible for inclusion in the final multivariable meta-regression model. Sampling methodology used (probability-based or nonprobability-based) was not associated with HCV prevalence (p > 0.1). In the final multivariable meta-regression analysis, all variables remained statistically significant (p < 0.05) with the exception of healthcare setting and unspecified study site. The final multivariable model explained 71.7% of the variability in HCV prevalence. Of note, the model indicated a statistically significant declining trend in HCV prevalence in Iran—year of data collection had an AOR of 0.93 (95% CI: 0.91–0.96).

**Table 6:** Univariable and multivariable meta-regression models for the mean HCV prevalence among populations in Iran.

		Number of studies	Univariable analysis		Multivariable analysis^a^	
			OR (95% CI)	p-value	AOR (95% CI)	p-value
Population classification	General population (low risk)	122	1	—	1	—
	PWID	56	269.41 (175.01–414.72)	0.000	88.80 (52.30–150.75)	0.000
	HIV patients	25	273.98 (152.38–492.64)	0.000	135.48 (72.30–253.87)	0.000
	Populations at high risk of healthcare-related exposures	127	48.08 (34.26–67.46)	0.000	39.33 (25.69–60.20)	0.000
	Populations at intermediate risk	70	10.10 (6.76–15.10)	0.000	4.36 (2.74–6.94)	0.000
	Populations with liver-related conditions	28	16.34 (9.34–28.61)	0.000	10.24 (5.65–18.55)	0.000
	Other special clinical populations	44	7.22 (4.51–11.55)	0.000	6.01 (3.61–10.02)	0.000
Study site	Community	42	1	—	1	—
	Blood bank	73	0.31 (0.15–0.64)	0.002	0.45 (0.7–0.77)	0.003
	Prison	44	28.12 (12.6–62.71)	0.000	3.66 (2.05–6.52)	0.000
	Rehab/Drop-in-center	35	48.49 (20.71–113.56)	0.000	2.26 (1.19–4.33)	0.013
	Healthcare setting	256	5.72 (3.08–10.62)	0.000	0.64 (0.40–1.02)	0.063
	Unspecified	22	3.74 (1.41–9.96)	0.008	1.11 (0.56–2.18)	0.766
Sampling methodology	Probability-based	52	1	—	1	—
	Nonprobability-based	390	1.7 (0.88–3.46)	0.114	—	—
	Unspecified	30	1.81 (0.62–5.26)	0.274	—	—
Sampling size	<100	144	1	—	1	—
	≥100	328	0.28 (0.18–0.44)	0.000	0.70 (0.53–0.92)	0.010
Year of data collection		472	0.95 (0.90–1.00)	0.032	0.93 (0.91–0.96)	0.000
Year of publication		472	1.00 (0.91–1.02)	0.150	—	—

^a^The adjusted R-square for the full model was 71.74%. Abbreviations: OR= Odds ratio; AOR= Adjusted odds ratio; CI = Confidence interval; PWID = People who inject drugs.

## Discussion

We presented a comprehensive systematic review and synthesis of HCV epidemiology in Iran. The pooled mean HCV prevalence in the general population was estimated at only 0.3%, on the lower side of the levels observed in other MENA countries^7,9,21–25,27^ and globally^75–77^. Despite this low prevalence in the general population, high prevalence was found among PWID and populations at high risk of healthcare-related exposures. These findings suggest that most ongoing HCV transmission in Iran is driven by injecting drug use and specific healthcare-related exposures. Genotypes 1 and 3 were the most frequently circulating strains. Of note, HCV prevalence in Iran is on a declining trend (Table 6).

Our estimate for the general population is slightly lower than an estimate provided for the whole adult population as part of a global estimation using a different methodology—0.3% in our study versus 0.5% in *Gower et al*.^78^. The difference may be explained by the fact that our estimate is strictly for the general (normally healthy) population. Moreover, our estimate is a pooled estimate of 122 studies as opposed to *Gower’s et al*. estimate which was based on five studies^78^. Inclusion of blood donor studies in our estimation did not explain the difference—our sensitivity analysis showed that estimated HCV prevalence in the general population was invariable with exclusion of blood donors (Fig. S5).

Iran has one of the highest population proportion of current PWID in the adult population (0.43%) in MENA, with an estimate of 185,000 current PWID^16,79^. Our synthesis indicated that injecting drug use was one of the most commonly reported risk factors for HCV infection, and that the pooled mean HCV prevalence among PWID was 52.2% (Table 4). These results suggest that injecting drug use is a main driver, if not the main driver, of HCV incidence in this country (Table 6). The regional context of Iran and the drug trafficking routes^21,80,81^, support an environment of active injection and a major role for PWID in HCV transmission. In this regard, HCV epidemiology in Iran appears to resemble that in developed countries, such as in the United States of America (USA), where most HCV incidence is attributed to drug injection^17,82,83^. Of note, we identified high HCV prevalence even among drug users where the route of drug use was not specified or excluded drug injection. This may suggest under-reporting of drug injection among those who report just drug use, or past drug injection among them before shifting to other forms of drug use.

Having said so, the estimated low HCV prevalence in the general population of only 0.3% apparently contradicts with a large PWID population in Iran. In the USA, it is estimated that the population proportion of current PWID is 0.3%^84^, and that of lifetime PWID is 2.6%^84^, compared to 0.43% for current PWID in Iran^16^. HCV prevalence among PWID in the USA is just over 50%^85^, therefore comparable with the pooled estimate of 52.2% for PWID in Iran (Table 4). HCV prevalence in the wider adult population in the USA is estimated at 1.0%^86^, much higher than the pooled estimate for HCV prevalence in the general population in Iran (0.3%). This discrepancy may be explained by an over-estimated current PWID population in Iran, very recent trend of drug injection with relatively small lifetime PWID population, or that the estimated HCV prevalence in the general population considerably underestimates the actual HCV prevalence in the whole adult population in Iran.

Our synthesis suggests that prisons have been a major setting for HCV transmission in Iran (Table 6). With nearly 60% of prisoners being incarcerated for drug-related offences^81^, high reported injecting risk behaviors in prisons^16,28^, and the high HCV prevalence among prisoners (Tables 3 and S2), prisons should be a main focus of HCV prevention and treatment efforts. Iran has made major and internationally-recognized strides in establishing harm reduction services for PWID including in prisons^16,33,87–90^, but further scale-up of these services in all prisons is warranted.

High HCV prevalence was found in populations at high risk of healthcare-related exposures such as hemodialysis, hemophilia, and thalassemia patients, though with geographical variation (Tables 3 and 6). This finding, along with the higher HCV prevalence generally among clinical populations (Table S3), suggests that healthcare is also a main driver of HCV transmission, though less so than in most other MENA countries^7,9,21–25,27^. The quality of healthcare and application of stringent protocols for infection control appear also to vary by setting within Iran. Overall, however, Iran seems to have made major progress in reducing HCV exposures through healthcare, which may explain the declining trend in HCV prevalence (Table 6)^91–93^. For example, HCV prevalence among hemodialysis patients was reported in one study to have declined from 14.4% in 1999 to 4.5% in 2006^94^.

HCV genotype 1 was the dominant circulating strain in Iran (56% of infections), followed by genotype 3 (39% of infections). This shows similarity to the pattern observed in multiple countries globally^95^. Nevertheless, this genotype distribution differs substantially from that found in most other MENA countries^29^. Several recent studies have also indicated an increasing presence of genotype 3^96,97^. This shift may be due to the fact that injecting drug use is a major driver of HCV incidence^29,98^ (Table 6), or the fact that this is a sub-regional pattern—genotype 3 is the main circulating strain in neighboring Pakistan^29^.

Our meta-analyses confirmed high heterogeneity in estimated effect sizes (Table 4). This was expected, due to differences between studies in variables such as risk population, study site, sampling methodology, sample size, and year of data collection, among others. Our meta-regressions identified several sources of heterogeneity in HCV prevalence studies in Iran. As expected, large differences in HCV prevalence by risk population were observed (Table 6). A small-study effect was also observed, with small studies reporting higher HCV prevalence. Importantly, a time trend was also observed with a declining HCV prevalence with time.

Our study is limited by the quality of available studies, as well as their representativeness of the different risk populations. High heterogeneity in prevalence measures were identified in all meta-analyses for all risk populations (Table 4). Meta-regression analyses were performed to identify the sources of heterogeneity, and while the final multivariable regression model accounted for 71.7% of observed heterogeneity, there are variables that we are unable to assess, such as “hidden” selection bias in recruitment.

Another limitation is the absence of reporting of the specific used biological assay in 25.0% of studies. The majority of included studies were based on convenience sampling. Although this is presumed a limitation, the meta-regression analyses did not identify sampling methodology as a statistically significant source of heterogeneity in HCV prevalence (p = 0.114; Table 6).

Despite these limitations, the main strength of our study is that we identified a very large number of studies, in fact the largest of all MENA countries^7,9,21–25,27^, that covered all risk populations and that allowed us to have such a comprehensive synthesis of HCV epidemiology.

## Conclusions

HCV prevalence in the wider population in Iran appears to be considerably below 1%—on the lower range compared to HCV prevalence in other MENA countries and globally. However, high HCV prevalence was found among PWID and populations at high risk of healthcare-related exposures. Most ongoing HCV transmission appears to be driven by injecting drug use and specific healthcare-related exposures. Genotypes 1 and 3 were the most frequently circulating strains.

There are still gaps in our understanding of HCV epidemiology in this country. Conduct of a nationally-representative population-based survey is strongly recommended to provide a better estimate of HCV prevalence in the whole population, delineate the spatial variability in prevalence, identify specific modes of exposure, and assess HCV knowledge and attitudes, as has been recently conducted in Egypt^10,99–103^ and Pakistan^6,15,104^.

Our study informs planning of health service provision, development of policy guidelines, and implementation of HCV prevention and treatment programming to reduce HCV transmission and decrease the burden of its associated diseases. Our findings suggest the need of a targeted approach to HCV control based on settings of exposure. Iran has established internationally-celebrated harm reduction services for PWID^16,87–90,105^, but these services need to be accessible to all PWID across the country, as well as in relevant settings, such as prisons. Further focus on infection control in healthcare facilities is also warranted, such as the adoption of the new WHO guidelines for the use of safety-engineered syringes^106,107^.

## Electronic supplementary material

Supplementary material

## References

[1] Alavian S, Fallahian F (2009). Epidemiology of Hepatitis C in Iran and the World. Shiraz E Medical Journal.

[2] Adler M, Goubau P, Nevens F, Van Vlierberghe H (2001). Hepatitis C virus: the burden of the disease. Acta gastro-enterologica Belgica.

[3] Stanaway JD (2016). The global burden of viral hepatitis from 1990 to 2013: findings from the Global Burden of Disease Study 2013. The Lancet.

[4] WHO. Global Hepatitis Report, http://www.who.int/hepatitis/publications/global-hepatitis-report2017/en/ (2017).

[5] The epidemiology of hepatitis C virus in the World Health Organization Eastern Mediterranean Region: Implications for strategic action. Eastern Mediterranean Hepatitis C Virus Epidemiology Synthesis Project. (in press).

[6] Umar M (2010). Hepatitis C in Pakistan: a review of available data. Hepatitis monthly.

[7] Al-Kanaani, Z., Kouyoumjian, S. P. & Abu-Raddad, L. J. The epidemiology of hepatitis C virus in Pakistan: systematic review and meta-analyses (under preparation). 10.1098/rsos.180257 PMC5936963 29765698

[8] Qureshi H, Bile K, Jooma R, Alam S, Afrid H (2010). Prevalence of hepatitis B and C viral infections in Pakistan: findings of a national survey appealing for effective prevention and control measures. Eastern Mediterranean Health Journal.

[9] Mohamoud YA, Mumtaz GR, Riome S, Miller D, Abu-Raddad LJ (2013). The epidemiology of hepatitis C virus in Egypt: a systematic review and data synthesis. BMC infectious diseases.

[10] El-Zanaty, F. & Egypt, W. A. Demographic and Health Survey 2008. Cairo: Egyptian Ministry of Health, National Population Council, El-Zanaty and Associates, and ORC Macro. https://dhsprogram.com/publications/publication-fr220-dhs-final-reports.cfm (2008) (2009).

[11] Brown RS, Gaglio PJ (2003). Scope of worldwide hepatitis C problem. Liver transplantation.

[12] A SPECIAL MEETING REVIEW EDITION: Advances in the Treatment of Hepatitis C Virus Infection From EASL 2014: The 49th Annual Meeting of the European Association for the Study of the Liver * April 9–13, 2014 * London, United Kingdom Special Reporting on:* SAPPHIRE II: Phase 3 Placebo-Controlled Study of Interferon-Free, 12-Week Regimen of ABT-450/R/ABT-267, ABT-333, and Ribavirin in Treatment-Experienced Adults With Hepatitis C Virus Genotype 1* All Oral Fixed-Dose Combination Sofosbuvir/Ledipasvir With or Without Ribavirin for 12 or 24 Weeks in Treatment-Naive Genotype 1 HCV-Infected Patients: the Phase 3 ION-1 Study* PEARL-III: 12 Weeks of ABT-450/R/267 + ABT-333 Achieved SVR in >99% of 419 Treatment-Naive HCV Genotype 1B-Infected Adults With or Without Ribavirin* Results of the Phase 2 Study M12-999: Interferon-Free Regimen of ABT-450/R/ABT-267 + ABT-333 + Ribavirin in Liver Transplant Recipients With Recurrent HCV Genotype 1 Infection* Sofosbuvir and Ribavirin for the Treatment of Chronic HCV With Cirrhosis and Portal Hypertension With and Without Decompensation: Early Virologic Response and Safety* All-Oral Dual Therapy With Daclatasvir and Asunaprevir in Patients With HCV Genotype 1B Infection: Phase 3 Study Results* Sofosbuvir/Ledipasvir Fixed Dose Combination Is Safe and Effective in Difficult-to-Treat Populations Including Genotype-3 Patients, Decompensated Genotype-1 Patients, and Genotype-1 Patients With Prior Sofosbuvir Treatment Experience* Sofosbuvir and Ribavirin for the Treatment of Recurrent Hepatitis C Infection After Liver Transplantation: Results of a Prospective, Multicenter Study PLUS Meeting Abstract Summaries With Expert Commentary by: Steven L. Flamm, MDChief, Liver Transplantation Program Professor of Medicine and Surgery Northwestern University Feinberg School of Medicine Chicago, Illinois. Gastroenterol Hepatol (N Y) 10, 1–19 (2014).

[13] WHO. Global health sector strategy on viral hepatitis 2016–2021. Towards ending viral hepatitis. Online at: http://www.who.int/hepatitis/strategy2016–2021/ghss-hep/en/ (2016).

[14] WHO. Combating hepatitis B and C to reach elimination by 2030: advocacy brief. Online at: http://www.who.int/hepatitis/publications/hep-elimination-by-2030-brief/en/ (2016).

[15] Qureshi, H., Bile, K., Jooma, R., Alam, S. & Afrid, H. Prevalence of hepatitis B and C viral infections in Pakistan: findings of a national survey appealing for effective prevention and control measures (2010). 21495584

[16] Mumtaz GR (2014). HIV among people who inject drugs in the Middle East and North Africa: systematic review and data synthesis. PLoS Med.

[17] Armstrong GL (2006). The prevalence of hepatitis C virus infection in the United States, 1999 through 2002. Annals of internal medicine.

[18] Zafarghandi MBS, Jadidi M, Khalili N (2015). Iran’s Activities on Prevention, Treatment and Harm Reduction of Drug Abuse. International journal of high risk behaviors & addiction.

[19] Razzaghi EM, Movaghar AR, Green TC, Khoshnood K (2006). Profiles of risk: a qualitative study of injecting drug users in Tehran, Iran. Harm Reduction Journal.

[20] Razzaghi, E., Rahimi Movaghar, A., Hosseini, M., Madani, S. & Chatterjee, A. Rapid Situation Assessment (RSA) of drug abuse in Iran. Prevention Department, State Welfare Organization, Ministry of Health, IR of Iran and United Nations International Drug Control Program (1999).

[21] Chemaitelly H, Mahmud S, Rahmani AM, Abu-Raddad LJ (2015). The epidemiology of hepatitis C virus in Afghanistan: systematic review and meta-analysis. International Journal of Infectious Diseases.

[22] Mohamoud YA, Riome S, Abu-Raddad LJ (2016). Epidemiology of hepatitis C virus in the Arabian Gulf countries: Systematic review and meta-analysis of prevalence. International Journal of Infectious Diseases.

[23] Chemaitelly H, Chaabna K, Abu-Raddad LJ (2015). The Epidemiology of Hepatitis C Virus in the Fertile Crescent: Systematic Review and Meta-Analysis. PLOS ONE.

[24] Fadlalla FA, Mohamoud YA, Mumtaz GR, Abu-Raddad LJ (2015). The Epidemiology of Hepatitis C Virus in the Maghreb Region: Systematic Review and Meta-Analyses. PloS one.

[25] Chaabna K, Kouyoumjian SP, Abu-Raddad LJ (2016). Hepatitis C virus epidemiology in Djibouti, Somalia, Sudan, and Yemen: systematic review and meta-analysis. PloS one.

[26] Ayoub H, Abu‐Raddad LJ (2017). Impact of treatment on hepatitis C virus transmission and incidence in Egypt: A case for treatment as prevention. Journal of viral hepatitis.

[27] Chaabna K, Mohamoud YA, Chemaitelly H, Mumtaz GR, Abu-Raddad LJ (2014). Protocol for a systematic review and meta-analysis of hepatitis C virus (HCV) prevalence and incidence in the Horn of Africa sub-region of the Middle East and North Africa. Systematic reviews.

[28] Heijnen, M., Mumtaz, G. R. & Abu-Raddad, L. J. Status of HIV and hepatitis C virus infections among prisoners in the Middle East and North Africa: review and synthesis. *Journal of the International AIDS Society* **19** (2016). 10.7448/IAS.19.1.20873 PMC4884676 27237131

[29] Mahmud, S. *et al*. Hepatitis C Virus Genotypes in the Middle East and North Africa: Distribution, Diversity, and Patterns. *Journal of Medical Virology* **90**, 131–141 (2018). 10.1002/jmv.24921 PMC5724492 28842995

[30] Kouyoumjian, S., Chemaitelly, H. & Abu-Raddad, L. Understanding the hepatitis C virus epidemic in Egypt: systematic reviews, meta-analyses, and meta-regression analyses (under review). 10.1038/s41598-017-17936-4 PMC5785953 29374178

[31] Higgins, J. P. & Green, S. *Cochrane handbook for systematic reviews of interventions*. Vol. 5 (Wiley Online Library, 2008).

[32] Moher D, Liberati A, Tetzlaff J, Altman DG (2009). Preferred reporting items for systematic reviews and meta-analyses: the PRISMA statement. Annals of internal medicine.

[33] Abu-Raddad, L. J. *et al*. Characterizing the HIV/AIDS epidemic in the Middle East and North Africa: time for strategic action. (Washington DC: The World Bank Press, 2010).

[34] Abu-Raddad LJ (2010). Epidemiology of HIV infection in the Middle East and North Africa. AidS.

[35] Choo Q-L (1989). Isolation of a cDNA clone derived from a blood-borne non-A, non-B viral hepatitis genome. Science.

[36] Kuo G (1989). An assay for circulating antibodies to a major etiologic virus of human non-A, non-B hepatitis. Science.

[37] Freeman MF, Tukey JW (1950). Transformations related to the angular and the square root. The Annals of Mathematical Statistics.

[38] Borenstein, M., Hedges, L. V., Higgins, J. P. T. & Rothstein, H. R. *Front Matter, in Introduction to Meta-Analysis*. (John Wiley & Sons, Ltd, 2009).

[39] Higgins JP, Thompson SG, Deeks JJ, Altman DG (2003). Measuring inconsistency in meta-analyses. BMJ: British Medical Journal.

[40] Higgins J, Thompson SG, Spiegelhalter DJ (2009). A re‐evaluation of random‐effects meta‐analysis. Journal of the Royal Statistical Society: Series A (Statistics in Society).

[41] Shannon CE (2001). A mathematical theory of communication. ACM SIGMOBILE Mobile Computing and Communications Review.

[42] R 3.1.2: A language and environment for statistical computing (Vienna, Austria, 2014).

[43] Schwarzer, G. General Package for Meta-Analysis. Version 4.1–0. Available at: http://cran.r-project.org/web/packages/meta/meta.pdf.

[44] StataCorp. Stata Statistical Software: Release 13. College Station, TX: StataCorp LP (2013).

[45] Azarkeivan A (2012). The incidence of hepatitis C in patients with thalassemia after screening in blood transfusion centers: A fourteen-year study. Transfusion.

[46] Jabbari A, Besharat S, Khodabakshi B (2008). Hepatitis C in hemodialysis centers of golestan province, northeast of Iran (2005). Hepatitis Monthly.

[47] Pourmand G (2007). Infectious complications after kidney transplantation: A single-center experience. Transplant Infectious Disease.

[48] Bahar A, Azizi F (2007). Insulin Resistance and β Cell Function in Patients with Chronic Hepatitis and Impaired Glucose Tolerance. Int J Endocrinol Metab.

[49] Dolan K (2012). Six-month follow-up of Iranian women in methadone treatment: drug use, social functioning, crime, and HIV and HCV seroincidence. Subst Abuse Rehabil.

[50] Mohtasham Amiri Z, Rezvani M, Jafari Shakib R, Jafari Shakib A (2007). Prevalence of hepatitis C virus infection and risk factors of drug using prisoners in Guilan province. Eastern Mediterranean Health Journal.

[51] Zamani S (2007). Prevalence and correlates of hepatitis C virus infection among injecting drug users in Tehran. International Journal of Drug Policy.

[52] Kheirandish P (2009). Prevalence and correlates of hepatitis C infection among male injection drug users in detention, Tehran, Iran. Journal of Urban Health.

[53] Ataei B, Tayeri K, Kassaian N, Farajzadegan Z, Babak A (2010). Hepatitis B and C among patients infected with human immunodeficiency virus in Isfahan, Iran: Seroprevalence and associated factors. Hepatitis Monthly.

[54] Merat S (2010). Seroprevalence of hepatitis C virus: The first population-based study from Iran. International Journal of Infectious Diseases.

[55] Mir-Nasseri MM, Mohammadkhani A, Tavakkoli H, Ansari E, Poustchi H (2011). Incarceration is a major risk factor for blood-borne infection among intravenous drug users: Incarceration and blood borne infection among intravenous drug users. Hepat Mon.

[56] Poustchi, H. *et al*. The impact of illicit drug use on Spontaneous Hepatitis C Clearance: Experience from a large cohort population study. *PLoS ONE* **6** (2011). 10.1371/journal.pone.0023830 PMC3161071 21887326

[57] Amin-Esmaeili M, Rahimi-Movaghar A, Razaghi EM, Baghestani AR, Jafari S (2012). Factors correlated with hepatitis C and B virus infections among injecting drug users in Tehran, IR Iran. Hepatitis Monthly.

[58] Kassaian, N. *et al*. Hepatitis C virus and associated risk factors among prison inmates with history of drug injection in Isfahan, Iran. *International Journal of Preventive Medicine* **3** (2012). PMC3399300 22826759

[59] Sarkari B (2012). High prevalence of hepatitis C infection among high risk groups in Kohgiloyeh and Boyerahmad Province, Southwest Iran. Archives of Iranian Medicine.

[60] Abedian S, Firoozi M, Malekzadeh R (2013). Etiology of hepatocellular carcinoma in IRAN: Single center experience in a large referral center, 2000–2011. Journal of Gastroenterology and Hepatology.

[61] Alipour A (2013). High prevalence of HCV coinfection in HIV-infected individuals in Shiraz, Islamic Republic of Iran. Eastern Mediterranean health journal = La revue de sante de la Mediterranee orientale = al-Majallah al-sihhiyah li-sharq al-mutawassit.

[62] Mir-Nasseri S (2005). HCV in intravenous drug users. Govaresh.

[63] Seyed Alinaghi S (2010). Prevalence and correlates of co-infection with human immunodeficiency virus and hepatitis C virus in male injection-drug users in Iran. Clinical Microbiology and Infection.

[64] Zamani S (2010). Prevalence of HIV/HCV/HBV infections and drug-related risk behaviours amongst IDUs recruited through peer-driven sampling in Iran. International Journal of Drug Policy.

[65] Nokhodian, Z. *et al*. Seroprevalence and risk factors of hepatitis C virus among juveniles in correctional center in Isfahan, Iran. *International Journal of Preventive Medicine* **3** (2012). PMC3399313 22826752

[66] Nokhodian Z (2012). Prevalence and risk factors of HIV, syphilis, hepatitis B and C among female prisoners in Isfahan, Iran. Hepatitis Monthly.

[67] Alipour, A., Rezaianzadeh, A., Hasanzadeh, J., Rajaeefard, A. & Davarpanah, M. A. Sexual transmission of hepatitis C virus between HIV infected subjects and their main heterosexual partners. *Hepatitis Monthly* **13** (2013). 10.5812/hepatmon.13593 PMC3859182 24348647

[68] Mehrjerdi ZA, Abarashi Z, Noroozi A, Arshad L, Zarghami M (2014). Correlates of shared methamphetamine injection among methamphetamine-injecting treatment seekers: The first report from Iran. International Journal of STD and AIDS.

[69] Salehi A, Naghshvarian M, Marzban M, Lankarani KB (2015). Prevalence of HIV, HCV, and High-Risk behaviors for substance users in drop in centers in southern Iran. Journal of Addiction Medicine.

[70] Zakizad M (2009). Seroprevalence of hepatitis C infection and associated risk factors among addicted prisoners in Sari-Iran. Pakistan journal of biological sciences: PJBS.

[71] Hosseini Asl SK, Avijgan M, Mohamadnejad M (2004). High prevalence of HBV, HCV, and HIV infections in gypsy population residing Shahr-e-Kord. Archives of Iranian Medicine.

[72] Azizi A, Amirian F, Amirian M (2011). Prevalence and Associated Factors of Hepatitis C in Self-introduced Substance Abusers. Hayat.

[73] Mansour-Ghanaei F (2007). Seroprevalence of hepatitis B and C among residents of Guilan Nursing Home. Hepat Mon.

[74] Samimi-Rad K, Hosseini M, Shahbaz B (2008). Hepatitis C virus infection and hcv genotypes of hemodialysis patients. Iranian Journal of Public Health.

[75] Lavanchy D (2011). Evolving epidemiology of hepatitis C virus. Clinical Microbiology and Infection.

[76] Mohd Hanafiah K, Groeger J, Flaxman AD, Wiersma ST (2013). Global epidemiology of hepatitis C virus infection: New estimates of age‐specific antibody to HCV seroprevalence. Hepatology.

[77] Cornberg M (2011). A systematic review of hepatitis C virus epidemiology in Europe, Canada and Israel. Liver International.

[78] Gower E, Estes C, Blach S, Razavi-Shearer K, Razavi H (2014). Global epidemiology and genotype distribution of the hepatitis C virus infection. Journal of hepatology.

[79] Mumtaz, G. R., Awad, S. F., Faizzadeh, A., Weiss, H. A. & LJ, A.-R. HIV incidence among people who inject drugs in the Middle East and North Africa: mathematical modeling analysis (under review). 10.1002/jia2.25102 PMC5867334 29577623

[80] United Nations Office on Drugs and Crime. World drug report, 2015. https://www.unodc.org/documents/wdr2015/World_Drug_Report_2015.pdf (2015).

[81] Calabrese J (2007). Iran’s War on Drugs: Holding the line. The Middle East Institute, Policy Brief.

[82] Alter MJ (2007). Epidemiology of hepatitis C virus infection. World Journal of gastroenterology.

[83] Shepard CW, Finelli L, Alter MJ (2005). Global epidemiology of hepatitis C virus infection. The Lancet infectious diseases.

[84] Lansky A (2014). Estimating the number of persons who inject drugs in the united states by meta-analysis to calculate national rates of HIV and hepatitis C virus infections. PLoS One.

[85] Armstrong GL (2006). The prevalence of hepatitis C virus infection in the United States, 1999 through 2002. Ann Intern Med.

[86] Denniston MM (2014). Chronic hepatitis C virus infection in the United States, National Health and Nutrition Examination Survey 2003 to 2010. Ann Intern Med.

[87] Zobeiri M, Adibi P, Alavian SM (2012). Intravenous drug use and hepatitis C virus in iran. Hepatitis monthly.

[88] Harm Reduction International. The global state of harm reduction 2012: Towards an integrated response. https://www.hri.global/global-state-of-harm-reduction-2012 (2012).

[89] Nissaramanesh, B., Trace, M. & Roberts, M. The rise of harm reduction in the Islamic Republic of Iran. *Beckley Foundation Drug Policy Programme, Briefing Paper* **8** (2005).

[90] Zafarghandi, M. B. S., Jadidi, M. & Khalili, N. Iran’s Activities on Prevention, Treatment and Harm Reduction of Drug Abuse. *International Journal of High Risk Behaviors and Addiction* **4** (2015). 10.5812/ijhrba.22863 PMC4744908 26870709

[91] Alavian S-M, Adibi P, Zali M-R (2005). Hepatitis C virus in Iran: Epidemiology of an emerging infection. Arch Iranian Med.

[92] Alavian S, Kafaee J, Yektaparast B, Hajarizadeh B, Doroudi T (2002). The efficacy of blood donor screening in reducing the incidence of hepatitis C virus infection among thalassemic patients in Iran. Transfusion Today.

[93] Azarkeivan A (2011). Evaluation of new cases of HCV infection in thalassaemia patients for source of infection. Asian journal of transfusion science.

[94] Alavian SM, Bagheri-Lankarani K, Mahdavi-Mazdeh M, Nourozi S (2008). Hepatitis B and C in dialysis units in Iran: changing the epidemiology. Hemodialysis international.

[95] Messina JP (2015). Global distribution and prevalence of hepatitis C virus genotypes. Hepatology.

[96] Taherkhani R, Farshadpour F (2015). Epidemiology of hepatitis C virus in Iran. World Journal of Gastroenterology.

[97] Sefidi FJ (2013). Distribution of hepatitis C virus genotypes in Iranian chronic infected patients. Hepatitis Monthly.

[98] Sefidi, F. J. *et al*. Distribution of hepatitis C virus genotypes in Iranian chronic infected patients. *Hepatitis monthly* **13** (2013). 10.5812/hepatmon.7991 PMC3582303 23550108

[99] Cuadros DF, Branscum AJ, Miller FD, Abu‐Raddad LJ (2014). Spatial epidemiology of hepatitis C virus infection in Egypt: analyses and implications. Hepatology.

[100] Miller FD, Abu-Raddad LJ (2010). Evidence of intense ongoing endemic transmission of hepatitis C virus in Egypt. Proceedings of the National Academy of Sciences.

[101] Chemaitelly H, Abu-Raddad LJ, Miller FD (2013). An apparent lack of epidemiologic association between hepatitis C virus knowledge and the prevalence of hepatitis C infection in a national survey in Egypt. PloS one.

[102] Benova L, Awad SF, Miller FD, Abu‐Raddad LJ (2015). Estimation of hepatitis C virus infections resulting from vertical transmission in Egypt. Hepatology.

[103] Guerra J, Garenne M, Mohamed M, Fontanet A (2012). HCV burden of infection in Egypt: results from a nationwide survey. Journal of viral hepatitis.

[104] Benova L, Awad SF, Abu‐Raddad LJ (2017). Estimate of vertical transmission of Hepatitis C virus in Pakistan in 2007 and 2012 birth cohorts. Journal of Viral Hepatitis.

[105] Abu-Raddad, L. J. *et al*. Policy Notes. Characterizing the HIV/AIDS epidemic in the Middle East and North Africa: Time for Strategic Action. Middle East and North Africa HIV/AIDS Epidemiology Synthesis Project. World Bank/UNAIDS/WHO Publication. (The World Bank Press, 2010).

[106] World Health Organization. WHO guideline on the use of safety-engineered syringes for intramuscular, intradermal and subcutaneous injections in health-care settings. http://apps.who.int/iris/handle/10665/250144 (2016). 27748094

[107] Organization, W. H. Making all injections safe. *Geneva, Switzerland: WHO* (2015).

[108] Afzali H, Taghavi A, Gholamreza G (2002). Seroepidemiology of hepatitis B and C in blood donors in Kashan, 1996–1999 [Persian]. Feyz.

[109] Aghajaanipour K, Zandieh T (2006). Seroepidemiology of hepatitis B, C and HIV in healthy blood donors in Babol city center in 2002 [Persian]. Blood Quarterly Journal.

[110] Alavi S (2012). Torque teno virus and hepatitis C virus co-infection in Iranian pediatric thalassemia patients. Turkish journal of haematology: official journal of Turkish Society of Haematology.

[111] Alavian SM, Gholami B, Masarrat S (2002). Hepatitis C risk factors in Iranian volunteer blood donors: A case-control study. Journal of Gastroenterology and Hepatology.

[112] Alavian SM (2015). Anti-hepatitis e antibody in hemodialysis patients in Isfahan, Iran: Prevalence and risk factors. Hepatitis Monthly.

[113] Amini S, Mahmoodabadi SA, Lamian S, Joulaie M, Farahani MM (2005). Prevalence of hepatitis G virus (HGV) in high-risk groups and blood donors in Tehran, Iran. Iranian Journal of Public Health.

[114] Ansar MM, Kooloobandi A (2002). Prevalence of hepatitis C virus infection in thalassemia and haemodialysis patients in north Iran-Rasht. Journal of Viral Hepatitis.

[115] Ansari-Moghaddam A (2012). Seroprevalence of Hepatitis B Surface Antigen and Anti Hepatitis C Antibody in Zahedan City, Iran: A Population-Based Study. Hepatitis Monthly.

[116] Ardebili M, Fattahi MR, Khademolhosseini F, Shirazi ZH, Doust FM (2012). Hepatitis C infection in a rural population in southern Iran: A report from kavar cohort study. Hepatology International.

[117] Arfaee R (2002). The prevalence of hepatitis B and C virus infection in war veterans of the 27th Islamic Revolutionary Guard Corps [Persian]. Military Medicine Magazine.

[118] Assarehzadegan MA, Shakerinejad G, Amini A, Rezaee SAR (2008). Seroprevalence of hepatitis E virus in blood donors in Khuzestan Province, Southwest Iran. International Journal of Infectious Diseases.

[119] Sayad B (2008). Seroepidemiology of hepatitis C in Kermanshah (West of Iran, 2006). Hepatitis Monthly.

[120] Barhaghtalab MY, Saboori S, Damiri M, Ekrahi M (2008). Prevalence of Viral Markers for Hepatitis B and C in Healthy Volunteer Blood Donors in Fasa Region, South Iran. International Journal of Infectious Diseases.

[121] Bozorgi SH (2012). Risk factors of viral hepatitis: yet to explore. Transfus Apher Sci.

[122] Chamani L (2007). Seroepidemiologic study of CMV, toxoplasma and hepatitis B and C in clients of Avicenna Infertility Clinic. Iran J Infect Dis Trop Med.

[123] Delavari M, Tabatabaei S (2004). Frequency of hepatitis C and its related factors in blood donors in Kerman in 2003. Annals of Military and Health Sciences Research.

[124] Doosti A, Arnini-Bavil-Olyaee S, Tajbakhsh E, Adeli A, Mahboudi F (2009). Prevalence of viral hepatitis and molecular analysis of HBV among voluntary blood donors in west Iran. New Microbiologica.

[125] Emamghorashi F, Fathi G, Mohtashami A (2006). Evaluation of demographic characteristics and hepatitis B, C and HIV prevalence among blood donors in Jahrom. SJIBTO.

[126] Fallahian F, Najafi A (2011). Epidemiology of hepatitis C in the Middle East. Saudi journal of kidney diseases and transplantation: an official publication of the Saudi Center for Organ Transplantation, Saudi Arabia.

[127] Esfandiarpour I, Zandi S, Rahnama Z, Dervish D (2005). Prevalence of anti-HCV-Ab (C) antibacterial antibody in psoriasis patients in Kerman [Persian]. Scientific Journal of Hamadan University of Medical Sciences & Health services.

[128] Esmaeili M, Mostafazadeh A, Sharbatdarn M, Hajiahmadi M, Alijanpoor M (2004). Hepatitis C in blood products receivers. Iranian Journal of Pediatrics.

[129] Esmaieli H, Hajiani G, Mankhian A, Poumehdi Broujeni M (2009). Seroepidemiological survey of hepatitis B, C, HIV and syphilis among blood donors in Bushehr-Iran. ISMJ.

[130] Farajzadeh S, Shakibi MR, Moghaddam SD, Rahnama Z (2005). Behcet disease: clinical spectrum and association with hepatitis B and C viruses. Eastern Mediterranean health journal = La revue de sante de la Mediterranee orientale = al-Majallah al-sihhiyah li-sharq al-mutawassit.

[131] Farshadpour F, Makvandi M, Samarbafzadeh AR, Jalalifar MA (2010). Determination of hepatitis C virus genotypes among blood donors in Ahvaz, Iran. Indian Journal of Medical Microbiology.

[132] Farshadpour F (2016). Prevalence and Trends of Transfusion-Transmissible Viral Infections among Blood Donors in South of Iran: An Eleven-Year Retrospective Study. PLoS One.

[133] Gachkar L (2005). Frequency of antibodies to hepatitis E virus among male blood donors in Tabriz. The Scientific Journal of Iranian Blood Transfusion Organization.

[134] Gerayli S (2015). The association between oral lichen planus and hepatitis C virus infection; a report from northeast of Iran. Jundishapur Journal of Microbiology.

[135] Ghaderi R, Makhmalbaf Z (2007). The Relationship between Lichen Planus and Hepatitis C in Birjand, Iran. Shiraz E-Med J.

[136] Ghadir M (2006). Hepatitis C in Golestan Province-Iran. Govaresh.

[137] Ghafouri M, Ameli M (2011). Comparing prevalence of transfusion transmitted viral infections in various population groups of South Khorasan. The Scientific Journal of Iranian Blood Transfusion Organization.

[138] Ghavanini AA, Sabri MR (2000). Hepatitis B surface antigen and anti-hepatitis C antibodies among blood donors in the Islamic Republic of Iran. Eastern Mediterranean health journal = La revue de sante de la Mediterranee orientale = al-Majallah al-ihhiyah li-sharq al-mutawassi.

[139] Ghezeldasht SA (2015). Oncogenic virus infections in the general population and end-stage renal disease patients with special emphasis on Kaposi’s Sarcoma Associated Herpes Virus (KSHV) in Northeast of Iran. Jundishapur Journal of Microbiology.

[140] Habibzadeh S, Davarnia B, Bagherzadeh J, Kholgh G (2005). Epidemiological evaluation of transfusion transmitted diseases in Ardabil in Tasoua and Ashoura 1381 (2003). The Scientific Journal of Iranian Blood Transfusion Organization.

[141] Hajiani E, Hashemi SJ, Masjedi-zade A, Cheraghi M (2006). Risk of Hepatitis C Virus transmission Following Upper Gastrointestinal Endoscopy. Yafteh.

[142] Hajiani E, Masjedizadeh R, Hashemi J, Azmi M, Rajabi T (2006). Hepatis C virus transmission and its risk factors within families of patients infected with hepatitis C virus in southern Iran: Khuzestan. World J Gastroenterol.

[143] Sharify Heydarabad H, Farid Soltany F, Montazam SH (2012). Seroepidemiology of sti viruses in pregnant women: A retrospective study 2010–2011. Journal of Sexual Medicine.

[144] Khedmat H (2009). Trends in seroprevalence of hepatitis B, hepatitis C, HIV, and syphilis infections in Iranian blood donors from 2003 to 2005. Hepatitis Monthly.

[145] Hosseini I (2007). Survey on the status of hepatitis C, B, AIDS and syphillis in blood donors in Bushehr Province in 2005 [Persian]. Infectious and Tropical Diseases of Iran.

[146] Jadali Z, Esfahanian F, Farhoud D, Alavian S, Soltan Dallal M (2005). Hashimoto’s Thyroiditis and Its Association with Hepatitis C Virus Infection. Int J Endocrinol Metab.

[147] Jadali Z, Esfahanian F, Eslami MB, Sanati MH (2005). Serum Antibodies against Hepatitis C Virus in Iranian Patients with Graves’ Disease. Iranian journal of allergy, asthma, and immunology.

[148] Jamali R (2008). Persistent alanine aminotransferase elevation among the general Iranian population: Prevalence and causes. World Journal of Gastroenterology.

[149] Amini Kafi-Abad S, Rezvan H, Abolghasemi H, Talebian A (2009). Prevalence and trends of human immunodeficiency virus, hepatitis B virus, and hepatitis C virus among blood donors in Iran, 2004 through 2007. Transfusion.

[150] Mowla K, Hajiani E (2008). Prevalence of Hepatitis C Virus Infection in Patients with Systemic Lupus Erythematosus: A Case-Control Study. Hepat Mon.

[151] Karimi A, Hoseini SM (2008). Seroprevalence of hepatitis B and C virus and HIV markers among blood donors from Shahre-Kord, Iran (2004–2006). Kuwait Medical Journal.

[152] Kasraian L, Tavassoli A (2008). Prevalence of hepatitis C and its risk factors in blood donors at Shiraz transfusion center. Koomesh.

[153] Kasraian L (2010). National disasters in iran and blood donation: Bam earthquake experience. Iranian Red Crescent Medical Journal.

[154] Kasraian L, Torab Jahromi SA (2007). Prevalence of major transfusion-transmissible viral infections in blood donors attending Fars Blood Transfusion Center, Shiraz, southern Iran: 2002–05. Iranian Journal of Medical Sciences.

[155] Kavoosi, H., Ebrahimi, A., Rezaei, M. & Jahani, M. Association of Lichen Planus with Hepatitis B and C. *Journal of Kermanshah University of Medical Sciences* **11** (2008).

[156] Kazemi Nejad V, Azar Housh R, Molana A (2005). & GR, D. Frequency of Hepatitis B virus, Hepatitis C virus and human immunodeficiency virus in blood donors and patients in Gorgan Blood Transfusion Organization in 2003 [Persian]. Journal of Gorgan University of Medical Sciences.

[157] Keshvari M, Sharafi H, Alavian SM, Mehrabadi H, Zolfaghari S (2015). Prevalence and trends of transfusion-transmitted infections among blood donors in Tehran, Iran from 2008 to 2013. Transfusion and Apheresis Science.

[158] Khedmat H (2007). Seroepidemiologic study of hepatitis B virus, hepatitis C virus, human immunodeficiency virus and syphilis infections in Iranian blood donors. Pakistan Journal of Biological Sciences.

[159] Aghamohamad, A., Montazeri, M. & Akbari, M. Prevalence of hepatitis B and hepatitis C in blood donors at Semnan province from 2008 to 2011. *Koomesh* **15**, Pe162–Pe167, En123 (2014).

[160] Kordi R, Neal K, Pourfathollah AA, Mansournia MA, Wallace WA (2011). Risk of hepatitis B and C infections in Tehranian wrestlers. Journal of athletic training.

[161] Mahmoodian-Shooshtari M, Pourfathollah A (2006). An overview analysis of blood donation in the Islamic Republic of Iran. Archives of Iranian Medicine.

[162] Maneshi HO, Zare S, Karimi M, Hajiani GR (2010). HBV and HCV viral markers seroperevalence in first-time healthy blood donors refered to transfusion centers of bushehr province, South of Iran (April 2004 to March 2008). Retrovirology.

[163] Mansour-Ghanaei F (2007). Prevalence of hepatitis B surface antigen and hepatitis C virus antibody and their risk factors among Guilan’s volunteer blood donors (1998–2003). Hepat Mon.

[164] Masaeli Z, Jaberi M, Magsudlu M (2006). A comparison of seroprevalence of blood-borne infections among regular, sporadic, and first-time blood donors in Isfahan. The Scientific Journal of Iranian Blood Transfusion Organization.

[165] Metanat M (2006). Prevalence of hepatitis C among diabetes mellitus patients in Zahedan. Zahedan Journal of Research in Medical Sciences.

[166] Moezzi M, Imani R, Karimi A, Pourheidar B (2015). Hepatitis C seroprevalence and risk factors in adult population of Chaharmahal and Bakhtiari province of Iran in 2013. Journal of Clinical and Diagnostic Research.

[167] Mogaddam MR, Anamzade F (2010). Survey of relationship between hepatitis C and lichen planus among dermatology outpatients of Imam Hospital of Ardabil city. Journal of Pakistan Association of Dermatologists.

[168] Mohammadali F, Pourfathollah A (2014). Association of ABO and Rh Blood Groups to Blood-Borne Infections among Blood Donors in Tehran-Iran. Iranian journal of public health.

[169] Mohebbi SR (2011). Hepatitis C and hepatitis B virus infection: Epidemiology and risk factors in a large cohort of pregnant women in Lorestan, West of Iran. Hepatitis Monthly.

[170] Moniri R, Mosayebii Z, Mossavi G (2004). Seroprevalence of cytomegalovirus, hepatitis B, hepatitis C and human immunodeficiency virus antibodies among volunteer blood donors. Iranian Journal of Public Health.

[171] Moradi, A. *et al*. Seroepidemiology of Rubella, Measles, HBV, HCV and B19 Virus Within Women in Child Bearing Ages (Saravan City of Sistan and Bloochastan Province). *Research Journal of Microbiology* **2** (2007).

[172] Motlagh ME, Makvandi M, M J (2001). Prevalence of anti-HCV among pregnant women. The Journal of Qazvin University of Medical Science.

[173] Beladi Mousavi SS, Hayati F, Ghorbani A (2010). Seroprevalence of cytomegalovirus antibody in renal transplant recipients and donors in Khuzestan Province, Iran. Shiraz E Medical Journal.

[174] Mousavi SSB, Hayati F (2011). Do we need to screen our patients for EBVIgGantibody before kidney transplantation?. Nephro-Urology Monthly.

[175] Pourshams A (2005). Prevalence and etiology of persistently elevated alanine aminotransferase levels in healthy Iranian blood donors. Journal of Gastroenterology and Hepatology.

[176] Rahbar AR, Rooholamini S, Khoshnood K (2004). Prevalence of HIV infection and other blood-borne infections in incarcerated and non-incarcerated injection drug users (IDUs) in Mashhad, Iran. International Journal of Drug Policy.

[177] Rahnama Z, Esfandiarpour I, Farajzadeh S (2005). The relationship between lichen planus and hepatitis C in dermatology outpatients in Kerman, Iran. International Journal of Dermatology.

[178] Razjou F, Maghsudlu M, Nasizadeh S, Zadsar M (2012). The impact of donor selection on blood safety in Iran. Transfusion and Apheresis Science.

[179] Rezaie M, Khaleghian A (2016). Prevalence of hepatitis B, hepatitis C and HIV in blood donors in Semnan Province (Iran) from 2011 to 2015. [Persian]. Koomesh.

[180] Rezazadeh M (2006). Prevalence of human immunodeficiency, hepatitis B and hepatitis C viruses in the first time, repeat and regular donors in blood transfusion center, Hamadan, 2004–2005. Iranian Journal of Infectious Diseases and Tropical Medicine.

[181] Nikbakht R, Saadati N, Firoozian F (2012). Prevalence of HBsAG, HCV and HIV antibodies among infertile couples in Ahvaz, South-West Iran. Jundishapur Journal of Microbiology.

[182] Salehi, H., Salehi, M., Ardestani, M. K., Khorvash, F. & Zadeh, K. M. Comparing the Blood Safety on the Blood Donors within the Religious Ceremonies and Routine Conditions. *Journal of Isfahan Medical School* **28** (2011).

[183] Samadi M (2014). The comparison of the prevalence rates of HBV, HCV, and HIV in blood donors having deferred for high risk behaviors. Scientific Journal of Iranian Blood Transfusion Organization.

[184] Seyed-Askari SM, Beigzadeh A, Mohammadpoor-Ravari M (2015). The prevalence of transfusion transmitted infections among blood donors in Kerman, Iran. [Persian]. Journal of Kerman University of Medical Sciences.

[185] Shaheli M, Yaghobi R, Rezaeian A, Saadi MI, Ramzi M (2015). Study of the associations between TT Virus single and mixed infections with leukemia. Jundishapur Journal of Microbiology.

[186] Javadzadeh Shahshahani H, Vaziri M, Mansouri F (2013). Seven Years Trends in Prevalence of Transfusion-Transmissible Viral Infections in Yazd blood Transfusion Organization. Iran J Ped Hematol Oncol.

[187] Shakeri, M. T. *et al*. The prevalence of hepatitis C virus in Mashhad, Iran: A population-based study. *Hepatitis Monthly* **13** (2013). 10.5812/hepatmon.7723 PMC3669679 23745128

[188] Shamsdin SA, Fattahi MR (2012). & Amirzadeh fard, S. The prevalence of hepatitis C infection in general population in Shiraz, southern Iran. International Journal of Infectious Diseases.

[189] Sofian M (2010). Lack of occult hepatitis B virus infection among blood donors with isolated hepatitis B core antibody living in an HBV low prevalence region of Iran. International Journal of Infectious Diseases.

[190] Sohrabpour A (2010). Prevalence of Nonalcoholic Steatohepatitis in Iran: A Population based Study. Middle East J Dig Dis.

[191] Tahereh Vahid M, Seyed-Moayed Alavian M, Ali Kabir M, Jafar Kafaee M (2005). Hepatitis B Prevalence and Risk Factors in Blood Donors in Ghazvin, IR. IranÍ. Hepatitis Monthly.

[192] Taheri Azbarmi Z (2008). Transfusion transmitted disease in Rasht blood donors. The Scientific Journal of Iranian Blood Transfusion Organization.

[193] Tajbakhsh E, Yaghobi R, Vahedi AR (2007). A serological survey on hepatitis C virus Antibody in blood donors with an ELISA method. Tehran University Medical Journal.

[194] A. Tanomand, H. M. A. M. K. Seroepidemiology of Hepatitis C Antibody Between Rural and Urban People: A Retrospective Study in June to December of 2005 in Malekan City, Iran. *Research Journal of Biological Sciences* **2**, 561–563 (2007).

[195] Vahidi A, Taheri A, Nikian Y (2000). Prevalence of Hepatitis C among Thalassemic patients referring to Kerman university of medical sciences hospital No. 1 in 1996. Journal of Kerman University of Medical Sciences.

[196] Yazdani M, Shakeri S (2006). Vertical transmission of hepatitis C from pregnant-mothers to their. Bimonthly Journal of Hormozgan University of Medical Sciences.

[197] Zamani, F. *et al*. Prevalence and risk factors of hepatitis C virus infection in Amol city, north of Iran: A population-Based study (2008–2011). *Hepatitis Monthly* **13** (2013). 10.5812/hepatmon.13313 PMC3867021 24358039

[198] Sorouri Zanjani R, Mazloomzadeh S, Koocheki A, Noori M (2013). Prevalence of Hepatitis B, C and HIV Infection in Blood Donors in Zanjan, 2005–2006. Preventive Care In Nursing & Midwifery Journal.

[199] Abdollahi A (2008). Seroprevalence of Human Immunodificiency Virus (HIV) and Hepatitis C Infection in Hemophilic Patients in Iran. Iranian Journal of Pathology.

[200] Aghakhani A, Ramezani A, Mohraz M, Banifazl M, Eslamifar A (2009). Significance of hepatitis B core antibody as the only marker of hepatitis B virus infection in high risk patients. Iranian Journal of Pathology.

[201] Akbari A, Imanieh M, Karimi M, Tabatabaee H (2011). Hepatitis C virus antibody positive cases in multitransfused thalassaemic patients in south of Iran. Hepatology International.

[202] Alvai S, Arzanian M, Hatami K, Shirani A (2005). Frequency of hepatitis C in thalassemic patients and its association with liver enzyme, MOfid Hospital, Iran, 2002. Pejouhesh.

[203] Alavi SM, Etemadi A (2007). HIV/HBV, HIV/HCV and HIV/HTLV-1 co infection among injecting drug user patients hospitalized at the infectious disease ward of a training hospital in Iran. Pakistan Journal of Medical Sciences.

[204] Alavi SM, Alavi L (2009). Seroprevalence study of HCV among hospitalized intravenous drug users in Ahvaz, Iran (2001–2006). Journal of Infection and Public Health.

[205] Alavia S (2003). The prevalence of Hepatitis B and C among thalassemia major patients in Ghazvin. Kowsar Medical Journal.

[206] Alavian S, Ardeshiri A, Hajarizadeh B (1994). Prevalence of HCV, HBV and HIV infections among hemophiliacs. J tongji Med Univ.

[207] Alipour A, Haghdoost AA, Sajadi L, Zolala F (2013). HIV prevalence and related risk behaviours among female partners of male injecting drugs users in Iran: Results of a bio-behavioural survey, 2010. Sexually Transmitted Infections.

[208] Alizadeh AHM, Alavian SM, Jafari K, Yazdi N (2005). Prevalence of hepatitis C virus infection and its related risk factors in drug abuser prisoners in Hamedan - Iran. World Journal of Gastroenterology.

[209] Mohammad Alizadeh A (2006). Frequencies of hepatitis B and C infections in hemophiliacs. Research in Medicine.

[210] Ameli M, Besharati S, Nemati K, Zamani F (2008). Relationship between elevated liver enzyme with iron overload and viral hepatitis in thalassemia major patients in Northern Iran. Saudi Medical Journal.

[211] Amiri ZM, Shakib AJ, Toorchi M (2005). Seroprevalence of hepatitis C and risk factors in haemodialysis patients in Guilan, Islamic Republic of Iran. Eastern Mediterranean Health Journal.

[212] Ansari H, Kamani H, Arbabi Sarjo A (2007). Prevalence of hepatitis C and related factors among beta-thalassemia major patients in Southern Iran in 2005-2006. Journal of Medical Sciences.

[213] Asl, R. T. *et al*. Outcome assessment of a triangular clinic as a harm reduction intervention in Rajaee-Shahr Prison, Iran. *Harm Reduction Journal* **10** (2013). 10.1186/1477-7517-10-41 PMC3883467 24369092

[214] Assarehzadegan MA, Boroujerdnia MG, Zandian K (2012). Prevalence of hepatitis B and C infections and HCV genotypes among haemophilia patients in Ahvaz, Southwest Iran. Iranian Red Crescent Medical Journal.

[215] Ataei B (2010). Seroepidemiology of hepatitis C in cases with history of intravenous drug use in Isfahan province, Iran. Clinical Microbiology and Infection.

[216] Ataei B (2011). Hepatitis c in intravenous drug users: seroprevalence and risk factors. J Isfahan Med Sch.

[217] Ataei, B. *et al*. Hepatitis C Screening in Intravenuos Drug Users in Golpayegan, Isfahan through Community Announcement: Pilot Study. *Journal of Isfahan Medical School* **28** (2011).

[218] Ataei, B. *et al*. Prevalence of anti HCV infection in patients with beta-thalassemia in Isfahan-Iran. *International Journal of Preventive Medicine* **3** (2012). PMC3399295 22826753

[219] Azarkeivan A (2010). Trace back of thalassemic patients with positive HCV markers to their donors in Adult Thalassemia Center. Sci J Blood Transfus Organ.

[220] Azarkeivan A (2011). Evaluation of clinical conditions of thalassemic patients having referred to Adult Thalassemia Center, Tehran. Sci J Iran Blood Transfus Organ.

[221] Babamahmoodi F, Gorji MAH, Nasehi MM, Delavarian L (2012). The prevalence rate of hepatitis B and hepatitis C co-infection in HIV positive patients in Mazandaran province, Iran. Medicinski Glasnik.

[222] Basirat Nia M, Asl S (2002). Determining the prevalence of hepatitis C in patients with thalassemia in Shahrekord, 1998 [Persian]. Research in Medicine.

[223] Boroujerdnia MG, Zadegan MAA, Zandian KM, Rodan MH (2009). Prevalence of hepatitis-C virus (HCV) among thalassemia patients in Khuzestan province, southwest Iran. Pakistan Journal of Medical Sciences.

[224] Bozorghi S (2006). Assessment of prevalence and risk factors of hepatitis C virus infection in haemodialysis patients in Ghazvin. The Scientific Journal of Iranian Blood Transfusion Organization.

[225] Bozorghi S (2008). The prevalence and risk factors of hepatitis C virus infection among thalassemic patients of Qazvin (2005). J Qazvin Univ Med Sci.

[226] Broumand B (2002). Prevalence of hepatitis C infection and its risk factors in hemodialysis patients in tehran: preliminary report from “the effect of dialysis unit isolation on the incidence of hepatitis C in dialysis patients” project. Saudi J Kidney Dis Transpl.

[227] Company F, Rezaei N (2007). Prevalence of hepatitis C and its relationship with glucose and diabetes mellitus in patients with major beta thalassemia [Persian]. Scientific Journal of Kurdistan University of Medical Sciences.

[228] Dadgaran S (2005). Prevalence and risk factors of hepatitis C virus among hemodialysis patients. Journal of Guilan University of Medical Sciences.

[229] Dadmanesh M (2015). Evaluation of prevalence and risk factors of hepatitis G virus infection among hemodialysis patients referred to Iranian Army Hospitals in Tehran during 2012–2013. Hepatitis monthly.

[230] Davarpanah MA (2013). Hepatitis C Virus Infection in HIV Positive Attendees of Shiraz Behavioral Diseases Consultation Center in Southern Iran. Indian J Community Med.

[231] Davoodian P, Dadvand H, Mahoori K, Amoozandeh A, Salavati A (2009). Prevalence of selected sexually and blood-borne infections in injecting drug abuser inmates of bandar abbas and roodan correction facilities, Iran, 2002. Brazilian Journal of Infectious Diseases.

[232] Eghbalian, F & AR, M. Study of prevalence of posttransfusion infections in Hamadan Thalassemic children. *Scientific Journal of Hamedan University of Medical Sciences* **17** (2000).

[233] Esfahani H, Bazmamoun H (2014). The prevalence of blood-borne viral infection (HBV, HCV, HIV) among hemophilia patients in Hamedan province of Iran. Iranian Journal of Blood and Cancer.

[234] Eskandarieh S (2013). Descriptive Aspects of Injection Drug Users in Iran’s National Harm Reduction Program by Methadone Maintenance Treatment. Iran J Public Health.

[235] Eslamifar A (2007). Hepatitis G virus exposure in dialysis patients. International Urology and Nephrology.

[236] Etminani-Esfahani M (2012). Serum vitamin D concentration and potential risk factors for its deficiency in HIV positive individuals. Current HIV Research.

[237] Faramarzi, H., Amini Lari, M., Marzban, M. & Shams, M. Hypogonadism and associated factors among men with HIV infection in Shiraz, Southern Iran. *Sexually Transmitted Infections* **89** (2013).

[238] Faranoush M (2006). Prevalence of hepatitis C resulted from blood transfusion in major thalassemia patients in Semnan, Damghan and Garmsar (2002). Medical Journal of Hormozgan University.

[239] Farhoudi B, SeyedAlinaghi S, Mohraz M, Hosseini M, Farnia M (2016). Tuberculosis, hepatitis C and hepatitis B co-infections in patients with HIV in the Great Tehran Prison, Iran. Asian Pacific Journal of Tropical Disease.

[240] Ghaderi A, Habib-Agahi M (1996). High prevalence of anti-HCV and HTLV-1 antibodies in thalassemia major patients of southern Iran. Irn J Med Sci.

[241] Ghadir, M., Movahhed, M., Movahhed, A., Movahhed, T. & Heidari, A. Effect of hepatitis C and B infections on anemia in hemodialysis patients. *Journal of Medical Council of Islamic Republic of Iran* **27**, Pe9–Pe15, En155 (2009).

[242] Ghafourian-Boroujerdnia M, Asarehzadegan M, Zandian K (2006). Seroprevalence of hepatitis B, hepatitis C and human immunodeficiency virus (HIV) among thalassemia patients refer to Ahwaz Shapha hospital (1999–2004). Jundishapur Journal of Microbiology.

[243] Ghane M, Eghbali M, Nejad HR, Saeb K, Farahani M (2012). Distribution of hepatitis C virus genotypes amongst the beta-thalassemia patients in north of Iran. Pakistan Journal of Biological Sciences.

[244] Haghazali S (2011). Occult HBV infection in hemodialysis patients in Qazvin. Razi Journal of Medical Sciences.

[245] Hamissi J, Hamissi H (2011). Occurrence of hepatitis B and C infection among hemodialyzed patients with chronic renal failure in Qazvin, Iran: A preliminary study. International Journal of Collaborative Research on Internal Medicine & Public Health.

[246] Hariri, M., Akbari, N., Yavari, F., Javadi, E. & Javer, S. In *Vox Sanguinis*. 268–268 (Wiley-Blackwell).

[247] Honarvar, B. *et al*. Blood-borne hepatitis in opiate users in Iran: A poor outlook and urgent need to change nationwide screening policy. *PLoS ONE* **8** (2013). 10.1371/journal.pone.0082230 PMC3846675 24312645

[248] Imani R, Karimi A, Rouzbahani R, Rouzbahani A (2008). Seroprevalence of HBV, HCV and HIV infection among intravenous drug users in Shahr-e-Kord, Islamic Republic of Iran. Eastern Mediterranean Health Journal.

[249] Ismail H (2005). Investigation of intravenous drug users and determining the rate of HIV and hepatitis virus in Loghman Hakim hospital [Persian]. Iranian Journal of Surgery.

[250] Joukar F, Besharati S, Mirpour H, Mansour-Ghanaei F (2011). Hepatitis C and hepatitis B seroprevalence and associated risk factors in hemodialysis patients in Guilan province, north of Iran: HCV and HBV seroprevalence in hemodialysis patients. Hepat Mon.

[251] Kaffashian, A. *et al*. The experience of hepatitis C screening among prison inmates with drug injection history. *Journal of Isfahan Medical School* **28** (2011).

[252] Kalantari H, Mirzabaghi A, Akbari M, Shahshahan Z (2011). Prevalence of hepatitis C virus, hepatitis B virus, human immunodeficiency virus and related risk factors among hemophilia and thalassemia patients in Iran. Iranian Journal of Clinical Infectious Diseases.

[253] Kalantari H, Ebadi S, Yaran M, Maracy MR, Shahshahan Z (2014). Prevalence and risk factors of hepatitis B and C viruses among hemodialysis patients in Isfahan, Iran. Adv Biomed Res.

[254] Karimi M, Ghavanini AA (2001). Seroprevalence of hepatitis B, hepatitis C and human immunodeficiency virus antibodies among multitransfused thalassaemic children in Shiraz, Iran. Journal of Paediatrics and Child Health.

[255] Karimi M, Ghavanini AA (2001). Seroprevalence of HBsAg, anti-HCV, and anti-HIV among haemophiliac patients in Shiraz, Iran. Haematologia.

[256] Karimi M, Ardeshiri R, Yarmohammadi H (2002). Inherited coagulation disorders in southern Iran. Haemophilia.

[257] Kashef S (2008). Antiphospholipid antibodies and hepatitis C virus infection in Iranian thalassemia major patients. International Journal of Laboratory Hematology.

[258] Kassaian N (2011). Hepatitis C in patients with multi blood transfusion in Isfahan, Iran. Hepatology International.

[259] Keramat F, Eini P, Majzoobi MM (2011). Seroprevalence of HIV, HBV and HCV in persons referred to hamadan behavioral counseling center, west of Iran. Iranian Red Crescent Medical Journal.

[260] Keshvari M (2014). Seroepidemiology of human T-cell lymphotropic virus among Iranian adult thalassemic patients. Transfusion Medicine.

[261] Khani M, Vakili MM (2003). Prevalence and risk factors of HIV, hepatitis B virus and hepatitis C virus infections in drug addicts among Zanjan prisoners. Archives of Iranian Medicine.

[262] Khorvash, F., Fasihi Dastjerdi, M. & Emami Naeini, A. In *1st National Congress of Infection in Addicts*. 23–29.

[263] Khosravi A, Bahmani M, Ghezel-Sofla I (2010). Co-infection by hepatitis C virus in human immunodeficiency virus infected patients in southwest of Iran. Iranian Journal of Clinical Infectious Diseases.

[264] Davoudi-kiakalayeh A (2014). Blood safety among beta-thalassemia major patients: Ten year experience in northern Iran. Vox Sanguinis.

[265] Lak M, Peyvandi F, Mannucci PM (2000). Clinical manifestations and complications of childbirth and replacement therapy in 385 Iranian patients with type 3 von Willebrand disease. British Journal of Haematology.

[266] Langarodi K, Poorheravi H (2011). Prevalence of HCV among thalassemia patients in Shahid Bahonar hospital, Karaj. The Scientific Journal of Iranian Blood Transfusion Organization.

[267] Mahdaviani F, Saremi S, Rafiei M (2008). Prevalence of hepatitis B, C and HIV infection in thalassemic and hemophilic patients of Markazi province in 2004. The Scientific Journal of Iranian Blood Transfusion Organization.

[268] Mahdavimazdeh M, Hosseini-Moghaddam SM, Alavian SM, Yahyazadeh H (2009). Hepatitis B infection in hemodialysis patients in Tehran province, Iran. Hepatitis Monthly.

[269] Mak, V., Mombaini, H., Mirmoemen, S., Latefi, S. & Borhanpour, K. A study on the prevalence anti-hepatitis C virus among the hemodialysis patients referred to Sina hospital of Ahwaz. *Jundishapur Scientific Medical Journal*, 1–5 (2001).

[270] Makhlough A, Jamshidi M, Mahdavi MR (2008). Hepatitis C prevalence studied by polymerase chain reaction and serological methods in haemodialysis patients in Mazandaran, Iran. Singapore Medical Journal.

[271] Mansour-Ghanaei F (2002). Prevalence of hepatitis B and C seromarkers and abnormal liver function tests among hemophiliacs in Guilan (northern province of Iran). Medical Science Monitor.

[272] Mansour-Ghanael F (2009). Prevalence of hepatitis B and C infection in hemodialysis patients of Rasht (Center of Guilan Province, Northern part of Iran). Hepatitis Monthly.

[273] Taheri-Azbarmi Z (2009). Prevalence of Transfusion Transmitted Infections among patients with Major Thalassemia in Guilan, the north province of Iran. Transfusion.

[274] Mashayekhi SO (2011). Prevalence of blood transfusion induced hepatitis among patients with thalassemia in Tabriz. Hepatology International.

[275] Meydani M, Farzaneh S, Ajami B, Hasanzadeh A (2009). Seroprevalence of HTLV1, 2 virus among injection drug addicts in Isfahan, 2007–2008. Journal of Shahid Sadoughi University of Medical Sciences.

[276] Mirahmadizadeh, A., Kadivar, M., Hemmati, A. & Javadi, A. In *International Conference on AIDS*. 16.

[277] Mirahmadizadeh AR, Majdzadeh R, Mohammad K, Forouzanfar MH (2009). Prevalence of HIV and hepatitis C virus infections and related behavioral determinants among injecting drug users of drop-in centers in Iran. Iranian Red Crescent Medical Journal.

[278] Mirmomen S (2006). Epidemiology of hepatitis B, hepatitis C, and human immunodeficiency virus infections in patients with beta-thalassemia in Iran: A multicenter study. Archives of Iranian Medicine.

[279] Mobini G (2010). Prevalence of anti-HCV antibody and related risk factors among bleeding disorder patients in Yazd province of Iran. Journal of Shahrekord University of Medical Sciences.

[280] Mohammadi, M. *et al*. Survey of both Hepatitis B Virus (HBsAg) and Hepatitis C Virus (HCV-Ab) coinfection among HIV positive patients. *Virology Journal* **6** (2009). 10.1186/1743-422X-6-202 PMC2785785 19922624

[281] Momen-Heravi M, Afzali H, Moosavipanah H (2012). Prevalence of anti HIV, ANTIHCV and, HBSAG positive among injection drug users in kashan-Iran. Journal of Clinical Immunology.

[282] Mousavi F (2002). A. B. M. A-IFN treatment of hepatitis C in thalassemic patients. Pejouhandeh.

[283] Mousavian S, Mansouri F, Saraei A, Sadeghei A, Merat S (2011). Seroprevalence of hepatitis C in hemophilia patients refering to Iran Hemophilia Society Center in Tehran. Govaresh.

[284] Naini MM, Derakhshan F, Hourfar H, Derakhshan R, Rajab FM (2007). Analysis of the related factors in hepatitis C virus infection among hemophilic patients in Isfahan, Iran. Hepatitis Monthly.

[285] Najafi N, Baba Mohammadei F, Azizi S (2001). Prevalence of chronic hepatitis C in patients with positive HCV-Ab thalassemia referring to Razi clinic of Ghaemshahr hospital in 1998 [Persian]. Journal of Mazandaran University of Medical Sciences.

[286] Rahimi-Movaghar A, Razaghi EM, Sahimi-Izadian E, Amin-Esmaeili M (2010). HIV, hepatitis C virus, and hepatitis B virus co-infections among injecting drug users in Tehran, Iran. International Journal of Infectious Diseases.

[287] Ramezani A (2008). Frequency and associated factors of proteinuria in Iranian HIV-positive patients. International Journal of Infectious Diseases.

[288] Ramezani A (2009). Frequency of isolated hepatitis B core antibody in HIV-hepatitis C virus co-infected individuals. Int J STD AIDS.

[289] Ramezani A (2014). HCV, HBV, and HIV seroprevalence, coinfections, and related behaviors among male injection drug users in Arak, Iran. AIDS Care - Psychological and Socio-Medical Aspects of AIDS/HIV.

[290] Rostami, Z., Lessan Pezeshki, M., Solemani Najaf Abadi, A. & Einollahi, B. Health related quality of life in iranian hemodialysis patients with viral hepatitis: Changing epidemiology. *Hepatitis Monthly* **13** (2013). 10.5812/hepatmon.9611 PMC3768204 24032050

[291] RostamiJalilian M, Omid Ghaemi M, Kassaeian N (2006). Relationship of Hepatitis B and C with Deep Vein Thrombosis in I.V Drug Abusers. Journal of Military Medicine.

[292] Sabour B, Boroumand P, Elah Y, Ghanbari M, Zarrinfam H (2003). Prevalence and risk factors of hepatitis C infection in hemodialysis patients (Kermanshah, 1999–2000). Journal of Kerman University of Medical Sciences.

[293] Saleh M, Mohammad K, Saleh A, Asghar H, Rasool S (2011). Prevalence of HIV, hepatitis B and C seropositivity in expired IV drug abusers in Hamedan. Scientific Journal of Forensic Medicine.

[294] Sali, S., Nikbin, M., Yadegarinia, D., Hesseinifar, M. & Shahmuradi, M. HIV and viral hepatitis (B,D,c) co-infection, genotyping, epidemiological profile in west of Tehran. *Sexually Transmitted Infections* **89** (2013).

[295] Sammak, H., Qomi, H. & Bitarafan, M. Prevalence of hepatitis B, C and HIV in patients with major β thalassaemia in Qom, 2007. *Qom University of Medical Sciences Journal* **4**, Pe17–Pe20, En13 (2010).

[296] Samarbaf-Zadeh AR (2015). Prevalence of hepatitis G virus among hemodialysis and kidney transplant patients in Khuzestan Province, Iran. Jundishapur Journal of Microbiology.

[297] Samimi-Rad K, Shahbaz B (2007). Hepatitis C virus genotypes among patients with thalassemia and inherited bleeding disorders in Markazi province, Iran. Haemophilia.

[298] Samimi-Rad K (2006). Prevalence of hepatitis C by RT-PCR in patients with thalassemia and hemophilia in Isfahan province 2005 [Persian]. Infectious and Tropical Diseases of Iran.

[299] Samimi-Rad K, Hosseini M (2008). Hepatitis C virus infection and hcv genotypes of hemodialysis patients. Iranian Journal of Public Health.

[300] Sanei-Moghaddam E, Savadkoohi S, Rakhshani F (2004). Prevalence of hepatitis B and C in patients with major Beta-thalassaemia referred to Ali-Asghar hospital In Zahedan, 2002. Blood Sci J Iran.

[301] Tavanaee S, Khaleghi N (2012). Epidemiological evaluation and some species in injection drug users that admitted in infectious department of Imam Reza hospital (2007–2009). Journal of Medical Council of Islamic Republic of Iran.

[302] SeyedAlinaghi S (2011). Hepatitis-C and hepatitis-B co-infections in patients with human immunodeficiency virus in Tehran, Iran. Acta medica Iranica.

[303] Seyrafian, S., Mobasherizadeh, S., Javadi, A., Akhzari, M. & Esfandiari, J. In *Nephrology Dialysis Transplantation*. 484–484 (Oxford University Press).

[304] Javadzadeh S, Attar M, Yavari M, Savabieh S (2006). Study of the prevalence of hepatitis B,C and HIV infection in hemophilia and thalassemia populations of Yazd. The Scientific Journal of Iranian Blood Transfusion Organization.

[305] Sharif M, Sherif A, Sayyah M (2009). Frequency of HBV, HCV and HIV infections among hospitalized injecting drug users in Kashan. Indian Journal of Sexually Transmitted Diseases.

[306] Sharifi-Mood B (2006). Viral infection among patients with hemophilia in the Southeast of Iran. Journal of Medical Sciences.

[307] Sharifi-Mood B, Eshghi P, Sanei-Moghaddam E, Hashemi M (2007). Hepatitis B and C virus infections in patients with hemophilia in Zahedan, southeast Iran. Saudi Medical Journal.

[308] Vaziri S (2008). Hepatitis D virus infection among HIV-HBV co-infected patients in Kermanshah, West of Iran. Hepatitis Monthly.

[309] Sofian M (2012). Viral hepatitis and HIV infection among injection drug users in a central iranian city. Journal of Addiction Medicine.

[310] Somi, M. H., Ardalan, M. R., Sokhanvar, H., Farhang, S. & Pouri, A. Hepatitis C virus infection in dialysis centers of Tabriz, Iran: a multicenter study. *Archives of Clinical Infectious Diseases* **2** (2007).

[311] Somi, M. H. *et al*. Risk factors of HCV seroconversion in hemodialysis patients in Tabriz, Iran. *Hepatitis Monthly* **14** (2014). 10.5812/hepatmon.17417 PMC4071359 24976839

[312] Taremi, M., Khoshbaten, M., Gachkar, L., Ehsani Ardakani, M. J. & Zali, M. R. Hepatitis E virus infection in hemodialysis patients: A seroepidemiological survey in Iran. *BMC Infectious Diseases* **5** (2005). 10.1186/1471-2334-5-36 PMC1156896 15904504

[313] Tayeri K, Kasaeian N, Fadaei N, Ataei B (2008). The prevalence of hepatitis B, hepatitis C and associated risk factors in intravenous drug addicts (IVDA) with HIV in Isfahan. Journal of Isfahan Medical School.

[314] Taziki O, Espahbodi F (2008). Prevalence of hepatitis C virus infection in hemodialysis patients. Saudi journal of kidney diseases and transplantation: an official publication of the Saudi Center for Organ Transplantation, Saudi Arabia.

[315] Toosi MN (2007). Risk factors and seroprevalence of hepatitis B and C infections among hemodialysis patients in Tehran. Iranian Journal of Pathology.

[316] Torabi S (2005). Prevalence of hepatitis B and C in thalassemic patients of East Azarbaijan in 2003. The Scientific Journal of Iranian Blood Transfusion Organization.

[317] Torabi S (2006). Prevalence of hepatitis B, C and HIV in hemophiliac patients of East Azarbaijan in 2004. The Scientific Journal of Iranian Blood Transfusion Organization.

[318] Valizadeh N (2013). Seroprevalence of hepatitis C, hepatitis B and HIV viruses in hemophiliacs born 1985–2010 in west Azarbaijan of Iran. Asian J Transfus Sci.

[319] Yazdani, M. R., Kassaian, N., Ataei, B., Nokhodian, Z. & Adibi, P. Hepatitis C virus infection in patients with hemophilia in Isfahan, Iran. *International Journal of Preventive Medicine* **3** (2012). PMC3399298 22826775

[320] Aminzadeh Z, Korough S (2007). Seroepidemiology of HIV, hepatitis B, hepatitis C and syphillis among injection drug addicts admitted to Loghman Hakim hospital, Tehran [Persian]. Journal of Medical Microbiology.

[321] Zahedi MJ, Darvishmoghadam S (2004). Frequency of Hepatitis B and C infection among Hemophiliac patients in Kerman. Journal of Kerman University of Medical Sciences.

[322] Zahedi MJ, Moghaddam SD, Alavian SM, Dalili M (2012). Seroprevalence of hepatitis viruses B, C, D and HIV infection among hemodialysis patients in Kerman province, South-East Iran. Hepatitis Monthly.

[323] Zali M-R, Aghazadeh R, Nowroozi A, Amir-Rasouly H (2001). Anti-HCV antibody among Iranian IV drug users: is it a serious problem. Arch Iran Med.

[324] Ziaee M, Power M, Hosseini S, Azarkar G (2005). Evaluation of hepatitis C infection and its prevalence in hemophilic patients in Khorasan [Persian]. Horizon of Knowledge.

[325] Ziaee M, Zarban A, Malekinejad P, Akhbary H (2007). Evaluation of HGV viremia prevalence and its co-infection with HBV, HCV, HIV and HTLV-1 in hemophilic patients of Southern Khorassan, Iran. Hepat Mon.

[326] Ziaee M, Namaei MH, Azarkar G (2015). The prevalence of HTLV-1 and its co-infection with HCV, HBV and HIV in hemophilic patients. Pakistan Journal of Medical Sciences.

