# Supplementary material for: The epidemiology of hepatitis C virus in Iran: Systematic review and meta-analyses
Source: Sci Rep. 2018 Jan 9;8:150. doi: 10.1038/s41598-017-18296-9 (PMC5760657; doi:10.1038/s41598-017-18296-9)
Supplement: Supplementary file 1 — Supplementary material [file 41598_2017_18296_MOESM1_ESM.pdf]

SUPPLEMENTARY MATERIAL

The epidemiology of hepatitis C virus in Iran: Systematic review and meta-analyses

Sarwat Mahmud,[a] Vajiheh Akbarzadeh,[a,b] and Laith J. Abu-Raddad[a, b]

[a] *Infectious Disease Epidemiology Group, Weill Cornell Medical College in Qatar, Cornell University, Qatar Foundation - Education City, Doha, Qatar*

[b] *Department of Healthcare Policy and Research, Weill Cornell Medical College, Cornell University, New York, New York, USA*

Table S1. Preferred Reporting Items for Systematic Reviews and Meta-analyses (PRISMA) checklist[1].

Section/topic	#	Checklist item	Reported in main text on
**TITLE**			
Title	1	Identify the report as a systematic review, meta-analysis, or both.	p.1
**ABSTRACT**			
Structured summary	2	Provide a structured summary including, as applicable: background; objectives; data sources; study eligibility criteria, participants, and interventions; study appraisal and synthesis methods; results; limitations; conclusions and implications of key findings; systematic review registration number.	p. 2
**INTRODUCTION**			
Rationale	3	Describe the rationale for the review in the context of what is already known.	p. 3
Objectives	4	Provide an explicit statement of questions being addressed with reference to participants, interventions, comparisons, outcomes, and study design (PICOS).	p. 3-4
**METHODS**			
Protocol and registration	5	Indicate if a review protocol exists, if and where it can be accessed (e.g., Web address), and, if available, provide registration information including registration number.	p. 4
Eligibility criteria	6	Specify study characteristics (e.g., PICOS, length of follow-up) and report characteristics (e.g., years considered, language, publication status) used as criteria for eligibility, giving rationale.	p. 5-6
Information sources	7	Describe all information sources (e.g., databases with dates of coverage, contact with study authors to identify additional studies) in the search and date last searched.	p. 4-5 and S1 Fig
Search	8	Present full electronic search strategy for at least one database, including any limits used, such that it could be repeated.	S1 Fig
Study selection	9	State the process for selecting studies (i.e., screening, eligibility, included in systematic review, and, if applicable, included in the meta-analysis).	p. 5
Data collection process	10	Describe method of data extraction from reports (e.g., piloted forms, independently, in duplicate) and any processes for obtaining and confirming data from investigators.	p. 5
Data items	11	List and define all variables for which data were sought (e.g., PICOS, funding sources) and any assumptions and simplifications made.	p. 6-9
Risk of bias in individual studies	12	Describe methods used for assessing risk of bias of individual studies (including specification of whether this was done at the study or outcome level), and how this information is to be used in any data synthesis.	p. 9-10
Summary measures	13	State the principal summary measures (e.g., risk ratio, difference in means).	p. 6
Synthesis of results	14	Describe the methods of handling data and combining results of studies, if done, including measures of consistency (e.g., I^2) for each meta-analysis.	p. 7-9
Risk of bias across studies	15	Specify any assessment of risk of bias that may affect the cumulative evidence (e.g., publication bias, selective reporting within studies).	p. 8
Additional analyses	16	Describe methods of additional analyses (e.g., sensitivity or subgroup analyses, meta-regression), if done, indicating which were pre-specified.	p. 8

**RESULTS**			
Study selection	17	Give numbers of studies screened, assessed for eligibility, and included in the review, with reasons for exclusions at each stage, ideally with a flow diagram.	p. 10-11 and Fig 1
Study characteristics	18	For each study, present characteristics for which data were extracted (e.g., study size, PICOS, follow-up period) and provide the citations.	p. 11-12 and Tables 2 and 3
Risk of bias within studies	19	Present data on risk of bias of each study and, if available, any outcome level assessment (see item 12).	p. 16 and Table 5
Results of individual studies	20	For all outcomes considered (benefits or harms), present, for each study: (a) simple summary data for each intervention group (b) effect estimates and confidence intervals, ideally with a forest plot.	Tables 2 and 3, S2 Table and S3 Table, S3 Fig and S4 Fig
Synthesis of results	21	Present results of each meta-analysis done, including confidence intervals and measures of consistency.	p. 11-14 and Table 4
Risk of bias across studies	22	Present results of any assessment of risk of bias across studies (see Item 15).	p. 15-16, Table 5
Additional analysis	23	Give results of additional analyses, if done (e.g., sensitivity or subgroup analyses, meta-regression [see Item 16]).	p. 14-16 and S5 Fig, p.16 and Table 6
**DISCUSSION**			
Summary of evidence	24	Summarize the main findings including the strength of evidence for each main outcome; consider their relevance to key groups (e.g., healthcare providers, users, and policy makers).	p. 17-20
Limitations	25	Discuss limitations at study and outcome level (e.g., risk of bias), and at review-level (e.g., incomplete retrieval of identified research, reporting bias).	p. 20
Conclusions	26	Provide a general interpretation of the results in the context of other evidence, and implications for future research.	p. 20-21
**FUNDING**			
Funding	27	Describe sources of funding for the systematic review and other support (e.g., supply of data); role of funders for the systematic review.	p. 63

Fig S1. Data sources and search criteria for systematically reviewing hepatitis C virus (HCV) incidence and prevalence data in Iran.

1) Literature search on PubMed:

("Hepatitis C"[Mesh] OR "Hepatitis C Antibodies"[Mesh] OR "Hepatitis C Antigens"[Mesh] OR "Hepacivirus"[Mesh] OR "Hepatitis C"[Text] OR "Hepacivirus"[Text] OR "HCV"[Text]) AND ("Iran"[Mesh] or Iran*[text] or "Persia"[Mesh] or Persia*[text])

Searched: 27/06/16

Results: 443 citations

2) Literature search on EMBASE:

(Ovid 1988 to present)

(exp Iran/ OR Iran*.mp. OR Persia*.mp.) AND (exp Hepatitis C/ OR hepatitis C.mp. OR exp hepatitis C antibody/ OR exp hepatitis C antigen/ OR exp Hepatitis C virus/ OR HCV.mp. OR hepacivirus.mp.)

Searched: 27/06/16

Results: 772 citations

3) Literature search on Scientific Iranian Database (SID):

(Hepatitis)

Searched: 29/06/2016

Results: 1885 citations

4) Literature search on International AIDS Society (IAS) Abstract Archive:

(Iran)

Searched: 01/07/2016

Results: 354 citations

5) Literature search on Index Medicus for the Eastern Mediterranean Region (IMEMR WHO):

(HCV AND Iran)

Searched: 01/07/2016

Results: 242 citations

Fig S2. Flow chart of article selection for the systematic review of hepatitis C virus (HCV) genotypes, adapted from the PRISMA 2009 guidelines[1].

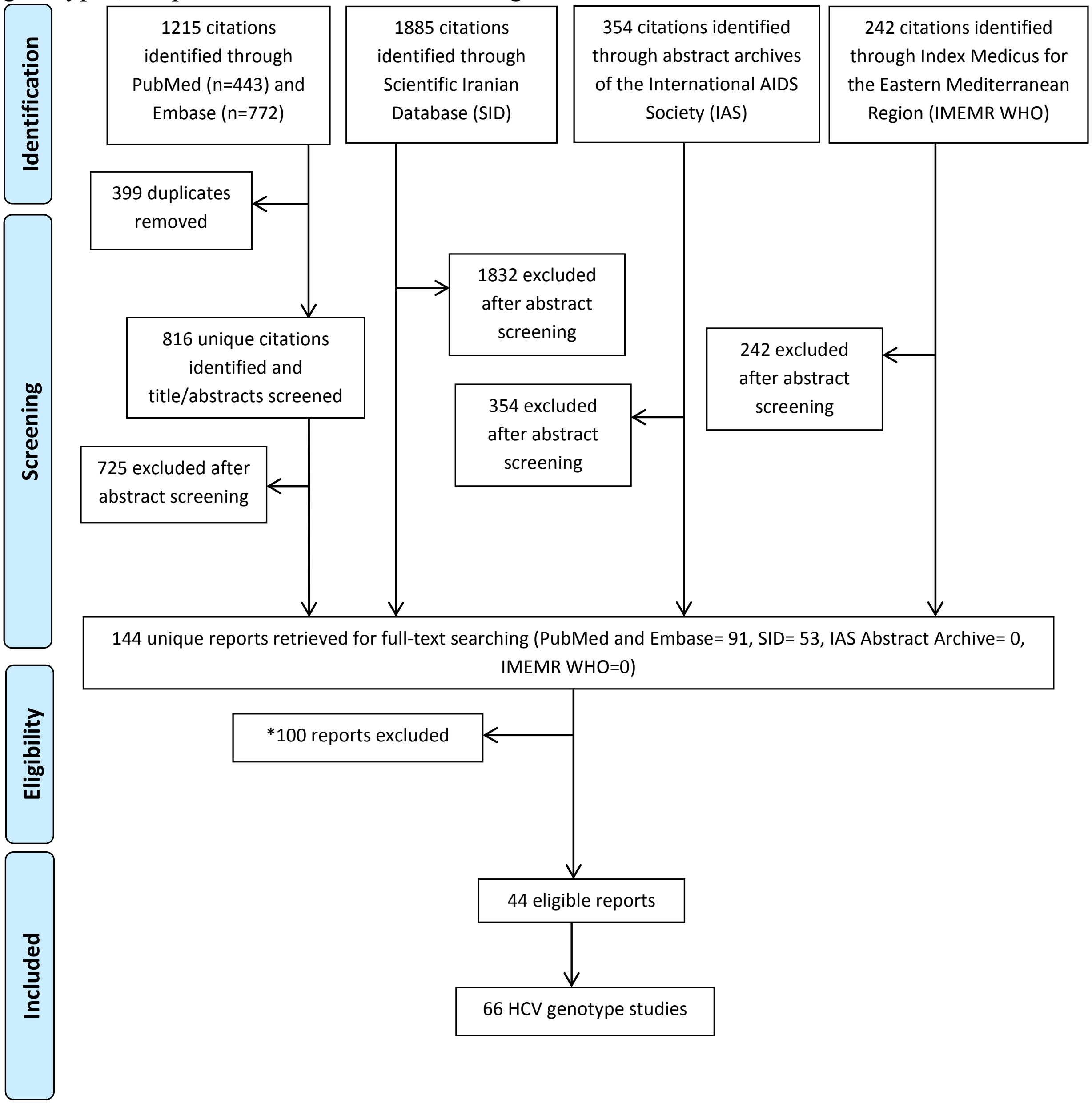

*Reasons for exclusion:

- Duplicate data (n= 11)
- Full-texts could not be retrieved and abstract does not have data on relevant outcomes (n= 55)
- Eligibility criteria not met (n= 27)
- Full-text did not include relevant indicators (n= 7)

Table S2. Studies reporting hepatitis C virus (HCV) prevalence among populations at intermediate risk in Iran.

Author, year (citation)	Year(s) of data collection	City or governorate	Study site	Study design	Sampling	Population	Sample size	HCV prevalence (%)
Akhoundi, 2014[2]	2011	Tehran	Community	CS	Conv	Dentists	1,628	0.06
Alavi-Naini, 2006[3]	NS	Zahedan	Community	CC	NS	Household contacts of index patients	159	3.1
Alipour, 2013[4]	NS	Mixed (Shiraz, Tehran, Mashhad)	Drop in centers and rehab centers	CS	Conv	Non-injecting spouses of PWID	184	8.4
Alipour, 2013[5]	2011	Shiraz	Counseling centers	CS	SRS	Sexual partners of HIV positive patients	168	9.5
Alizadeh, 2005[6]	2002	Hamedan	Prison	CS	SRS	Non-injecting drug users	278	29.1
Amiri, 2007[7]	2003	Guilan	Prison	CS	Conv	Non-infecting drug users	379	36.1
Amiri, 2014[8]	2012	Tehran	Primary health care centers (community)	CS	Conv	Homeless	593	23.3
Asgari, 2008[9]	2001-2005	Mixed	Prison	CS	NS	Prisoners	8,630	37.9
Asghar, 2003[10]	2002	Kermanshah	Clinical: hospital & health care centers	CS	Conv	Healthcare workers	110	0.0
Asl, 2004[11]	NS	Shahrekord	Community	CS	Conv	Homeless	226	3.1
Ataei, 2010[12]	2005-2007	Isfahan	Community	CS	Conv	Homeless	116	0.0
Ataei, 2010[12]	2005-2007	Isfahan	Community	CS	Conv	Homeless	270	1.5
Ataei, 2011[13]	2005-2007	Isfahan	Community	CS	Conv	Homeless	386	1.0
Azarkar, 2007[14]	NS	Birjand	Prison	CS	SRS	Prisoners	400	7.8
Azarkar, 2010[15]	2008	Birjand	Prison	CS	SRS	Non-injecting drug users	140	15.7
Azarkar, 2010[15]	2008	Birjand	Prison	CS	SRS	Prisoners	218	3.2
Azizi, 2011[16]	2007	Kermanshah	Drop in centers and rehab centers	CS	Conv	Non-injecting drug users	263	22.1
Bassirat-Nia, 2001[17]	1999	Shahrekord	Community	NS	Conv	Household contacts of index patients	136	0.0
Behnaz, 2004[18]	2002	Yazd	Dental Clinics	CS	Conv	Healthcare workers	104	0.0
Behnaz, 2007[19]	2002-2003	Gorgan	Prison	CS	SRS	Non-injecting drug users	121	28.1
Dolan, 2012[20]	2007-2008	Tehran	Drop in centers and rehab centers	CS	Conv	Non-injecting drug users	50	24.0

Author, year (citation)	Year(s) of data collection	City or governorate	Study site	Study design	Sampling	Population	Sample size	HCV prevalence (%)
Eilami, 2012[21]	2009-2010	Mixed (Kohgiloyeh and Boyerahmad)	Hospital	CS	NS	Healthcare workers	212	4.2
Fallah, 2008[22]	2007	Tehran	Counseling centers	CS	Conv	Homeless	203	3.5
Hajiani, 2006[23]	1998-2003	Ahvaz	Community	CC	Conv	Household contacts of index patients	300	1.3
Hasanpour, 2003[24]	2001-2003	Tehran	Clinical: hospital & health care centers	CS	Conv	Inpatients	3,714	1.5
Honarvar, 2013[25]	2012-2013	Shiraz	Counseling centers	CS	Conv	Non-injecting drug users	336	4.5
Imani, 2010[26]	2005-2008	Shahrekord	Community	CS	Conv	Household contacts of index patients	230	2.2
Jahani, 2005[27]	2002	NS	Prisoners	CS	Conv	Female sex workers	149	2.7
Kamangar, 2003[28]	2000-2001	Kerman	Clinical: hospital & health care centers	CS	Conv	Healthcare workers	285	2.1
Kaserani, 2007[29]	1999-2003	Kermanshah	Clinical: hospital & health care centers	CS	Conv	Inpatients	6,820	0.45
Kassaian, 2011[30]	2009-2010	Isfahan	Community, drop in centers and rehab centers	CS	Snowball sampling	Female sex workers	93	9.9
Khodadi, 2006[31]	2003	Rafsanjan	Drop in centers and rehab centers	CS	Conv	Non-injecting drug users	149	3.4
Lari, 2010[32]	2007-2008	Shiraz	Community	CS	Conv	Homeless	50	7.5
Metanet, 2006[33]	2004	Zahedan	Clinical: hospital & health care centers	CC	Conv	Diabetes patients	505	0.2
Moayedi-Nia, 2016[34]	2012-2013	Tehran	Community	CS	Snowball sampling	Female sex workers	161	8.1
Mohammadalizadeh, 2013[35]	1998	Hamadan	Prison	CS	Conv	Non-injecting drug users	479	27.4
Mousavijahed, 2001[36]	NS	Mixed (Imam Khomeinim Taleghani, Labafi)	Clinical: hospital & health care centers	Coh	Conv	Healthcare workers	240	0.83
Mousavijahed, 2001[36]	NS	Mixed (Imam Khomeinim Taleghani, Labafi)	Clinical: hospital & health care centers	Coh	Conv	Hospital administrative staff	400	0.0
Nasir, 2010[37]	NS	NS	Diabetes patients	NS	Conv	Diabetes patients	400	2.5
Nokhodian, 2012[38]	2008-2009	Isfahan	Prison	CS	Conv	Prisoners	160	4.4
Nokhodian, 2012[39]	2008-2009	Isfahan	Prison	CS	Conv	Prisoners	163	7.4

Author, year (citation)	Year(s) of data collection	City or governorate	Study site	Study design	Sampling	Population	Sample size	HCV prevalence (%)
Norouzian, 2016[40]	2012-2013	Lorestan	Drop in centers and rehab centers	CS	NS	Non-injecting drug users	271	16.2
Peyvandi, 2006[41]	2005-2006	Mashshad	Clinical: hospital & health care centers	CS	Conv	Inpatients	108	2.8
Peyvandi, 2006[41]	2005-2006	Mashshad	Clinical: hospital & health care centers	CS	Conv	Inpatients	562	3.9
Pourahmad, 2007[42]	2003	Mixed (Esfahan, Chaharmahal Bakthiary, Lorestan)	Prison	CC	Conv	Prisoners	1,431	34.7
Rostami, 2012[43]	2009	Anidmeshk	Clinical: hospital & health care centers	CS	NS	Patients referred to laboratory	205	0.49
Rostami, 2012[43]	2009	Anidmeshk	Clinical: hospital & health care centers	CS	NS	Patients referred to laboratory	486	0.41
Safaar, 2005[44]	2003	Sari	Clinical: hospital & health care centers	CS	Conv	Healthcare workers	280	0.37
Sharifi, 2008[45]	NS	Qazvin	Clinical: hospital & health care centers	CS	Conv	Healthcare workers	74	0.0
Shoaei, 2012[46]	2010	Isfahan	Clinical: hospital & health care centers	CS	SRS	Healthcare workers	203	0.0
Shoaei, 2013[47]	NS	Isfahan	Barber shops and beauty salons	CS	SRS	Barbers/barbers' clients/community healers	479	0.0
Tajbakhsh, 2008[48]	NS	Shahrekord	Prison	NS	NS	Prisoners	50	0.67
Tajbakhsh, 2008[48]	NS	Shahrekord	Prison	NS	NS	Prisoners	60	1.2
Tajbakhsh, 2008[48]	NS	Shahrekord	Prison	NS	NS	Prisoners	100	3.8
Tajbakhsh, 2008[48]	NS	Shahrekord	Prison	NS	NS	Prisoners	110	1.7
Tajbakhsh, 2008[48]	NS	Shahrekord	Prison	NS	NS	Prisoners	110	2.2
Tajbakhsh, 2008[48]	NS	Shahrekord	Prison	NS	NS	Prisoners	170	3.2
Talaie, 2007[49]	2004-2005	Tehran	Hospital	CS	Conv	Non-injecting drug users	169	14.5
Vahdani, 2006[50]	NS	Tehran	Community	CS	Conv	Homeless people	102	0.0
Vahdani, 2009[51]	2007	Tehran	Counseling centers	CS	Conv	Homeless people	202	34.3
Yarmohammadi, 2011[52]	2008	Shahroud	Clinical: hospital & health care centers	CS	Conv	Healthcare workers	191	0.0

Author, year (citation)	Year(s) of data collection	City or governorate	Study site	Study design	Sampling	Population	Sample size	HCV prevalence (%)
Zadeh, 2006[53]	2003	Rafsanjan	Prison	CS	Conv	Non-injecting drug users	180	7.2
Zakizad, 2009[54]	2001-2003	Sari	Prison	CS	SRS	Non-injecting drug users	312	30.8
Ziaee, 2014[55]	2009-2010	Southern Khorasan	Prison	CS	SRS	Prisoners	881	7.7

[a]Abbreviations: CC, case-control; Coh, cohort; Conv, convenience; CS, cross-sectional; NS, not specified; PWID, people who inject drugs; SRS, simple random sampling.
[b]The decimal places of the prevalence figures are as reported in the original reports, but prevalence figures with more than one decimal places were rounded to one decimal place, with the exception of those below 0.1%.

Table S3. Studies reporting hepatitis C virus (HCV) prevalence among special clinical populations in Iran.

Author, year (citation)	Year(s) of data collection	City or governorate	Study site	Study design	Sampling	Population	Sample size	HCV prevalence (%)
Abedian, 2013[56]	2000-2011	Tehran	Clinical: hospital & health care centers	CS	Conv	Cirrhosis patients (female)	303	7.4
Abedian, 2013[56]	2000-2011	Tehran	Clinical: hospital & health care centers	CS	Conv	Cirrhosis patients (male)	1,943	14.9
Abedian, 2013[57]	2000-2011	Tehran	Clinical: hospital & health care centers	CS	Conv	Hepatocellular carcinoma patients (Female)	62	5.6
Abedian, 2013[57]	2000-2011	Tehran	Clinical: hospital & health care centers	CS	Conv	Hepatocellular carcinoma patients (male)	176	10.2
Aledavood, 2014[58]	2007-2009	Mashhad	Clinical: hospital & health care centers	CS	Conv	Patients with NHL-B cell	128	0.78
Ardakani, 2009[59]	2001	Tehran	Clinical: hospital & health care centers	CS	Conv	Patients with rheumatoid arthritis	200	2.5
Azimi, 2002[60]	1994-1997	Tehran	Clinical: hospital & health care centers	CS	Conv	Cirrhosis patients	170	3.5
Baba Mahmoodi, 2010[61]	2003-2008	Razi	Clinical: hospital & health care centers	CS	Conv	Hepatitis patients	439	31.0
Bahar, 2006[62]	1998-2001	Tehran	Tehran Lipid and Glucose Study	Coh	Conv	Impaired glucose tolerance patients	560	0.36
Ebadi, 2011[63]	2007-2010	NS	Clinical: hospital & health care centers	CS	Conv	Renal transplant recipients	106	9.4
Ehsani, 2013[64]	2009-2010	Tehran	Clinical: hospital & health care centers	CC	Conv	Dermatology patients	60	1.7
Ehsani, 2013[64]	2009-2010	Tehran	Clinical: hospital & health care centers	CC	SRS	Patients with cutaneous disorders	230	1.3
Einollahi, 2003[65]	1995-2001	Tehran	Clinical: hospital & health care centers	Coh	Conv	Renal transplant patients	909	4.5
Esfandiarpour, 2005[66]	2002-2003	Kerman	Clinical: hospital & health care centers	CC	Conv	Psoriasis patients	132	2.3
Geryali, 2015[67]	2012	Mashhad	Clinical: hospital & health care centers	CC	Conv	Lichen planus patients	134	2.2
Ghaderi, 2007[68]	2004-2006	Birjand	Clinical: hospital & health care centers	CC	Conv	Lichen planus patients	73	4.1
Ghafari, 2008[69]	1990-2006	NS	Clinical: hospital & health care centers	CS	Conv	Renal transplant patients	1,350	3.2
Ghafourian-Boroujerdnia, 2013[70]	2009-2010	Ahvaz	Clinical: hospital & health care centers	CS	Conv	Sickle cell anemia patients	56	12.5
Gharagozloo, 2001[71]	NS	Tehran	Clinical: hospital & health care centers	CS	Conv	Patients with lymphoproliferative diseases	110	31.8

Author, year (citation)	Year(s) of data collection	City or governorate	Study site	Study design	Sampling	Population	Sample size	HCV prevalence (%)
Ghodsi, 2004[72]	1997-1998	Tehran	Clinical: hospital & health care centers	CC	Conv	Lichen planus patients	146	4.8
Hajiani, 2006[73]	2003-2004	Tehran	Clinical: hospital & health care centers	CC	Conv	Endoscopic/colonoscopy patients	456	1.1
Hajiani, 2009[74]	2002-2007	Khuzestan	Clinical: hospital & health care centers	CS	Conv	HBV patients	1,095	0.90
Hajiani, 2012[75]	1999-2008	Ahvaz	Clinical: hospital & health care centers	CS	Conv	Patients with liver cirrhosis	165	9.1
Jadali, 2005[76]	NS	NS	Clinical: hospital & health care centers	CC	Conv	Vitiligo patients	65	1.5
Jadali, 2005[77]	NS	Tehran	Clinical: hospital & health care centers	CC	Conv	Patients with Hashimoto's Thyroiditis	50	4.0
Karim, 2008[78]	2003-2005	Ahvaz	Clinical: hospital & health care centers	CC	Conv	Systemic lupus erythematosus patients	124	4.8
Kavoosi, 2008[79]	2004-2005	Kermanshah	Clinical: hospital & health care centers	CC	Conv	Lichen planus patients	57	1.7
Mamani, 2009[80]	2006-2007	Hamadan	Counseling center	CS	Conv	Chronic psychiatric patients	170	1.8
Mogaddam, 2010[81]	NS	Ardabil	Clinical: hospital & health care centers	CC	Conv	Lichen planus patients	60	1.7
Mousavi, 2010[82]	2008	Khuzestan	Clinical: hospital & health care centers	CS	Conv	Renal transplant recipients	69	2.9
Mousavi, 2011[83]	2010-2011	Khuzestan	Clinical: hospital & health care centers	CS	Conv	ESRD patients	1,117	3.4
Mousavi, 2011[83]	2009-2010	Ahvaz	Clinical: hospital & health care centers	CS	Conv	Renal transplant recipients	60	3.3
Ossareh, 2009[84]	2007	Tehran	Clinical: hospital & health care centers	CS	Conv	Renal transplant patients	300	1.7
Pourmand, 2007[85]	2002-2004	Isfahan	Clinical: hospital & health care centers	CS	Conv	Renal transplant patients	142	0.70
Pourshams, 2003[86]	NS	Tehran	Blood transfusion center	CS	Conv	Patients with ALT	52	7.7
Poustchi, 2012[87]	NS	NS	Clinical: hospital & health care centers	CC	Conv	Patients with Graves diseease	55	1.8
Rahimi, 2007[88]	2000-2005	Tehran	Clinical: hospital & health care centers	CS	Conv	Hepatitis patients	200	22.0
Rahnama, 2005[89]	2000-2001	Kerman	Clinical: hospital & health care centers	CC	Conv	Lichen planus patients	66	1.5
Ramezani, 2008[90]	NS	NS	Clinical: hospital & health care centers	CS	Conv	HBV patients	77	0.0
Razjou, 2012[91]	2003-2008	National	Blood transfusion center	CS	Conv	Deferred blood donors	40,107	0.58
Roshandel, 2008[92]	2004	Golestan	Clinical: hospital & health care centers	CS	Cluster sampling	HBV patients	139	12.3

Author, year (citation)	Year(s) of data collection	City or governorate	Study site	Study design	Sampling	Population	Sample size	HCV prevalence (%)
Saleh, 2011[93]	2007-2008	Hamedan	Clinical: hospital & health care centers	CC	Conv	Corpses of accidental death	94	5.3
Salehi, 2003[94]	1997-1999	Zahedan	Clinical: hospital & health care centers	CS	Conv	Viral hepatitis patients	273	1.8
Salehi, 2008[95]	2002-2002	Sistan & Baluchestan	Clinical: hospital & health care centers	CS	Conv	Acute viral hepatitis	263	2.3
Salem, 2013[96]	2008-2009	Alborz	Prison	CS	Conv	HBV prisoners	122	18.0
Samarbaf-Zadeh, 2015[97]	NS	Khuzestan	Clinical: hospital & health care centers	CS	Conv	Transplant patients	150	1.3
Samadi, 2014[98]	2012	Ahvaz	Blood transfusion center	CS	Conv	Deferred blood donors	619	1.3
Sanaei-Zadeh, 2002[99]	2000-2001	Tehran	Tehran Legal Medicine Organization	CS	SRS	Corpses	173	4.1
Semnani, 2007[100]	2004-2005	Golestan	Community	CS	Cluster sampling	HBV patients	138	12.3
Shaheli, 2015[101]	2012-2013	Shiraz	Clinical: hospital & health care centers	CS	Conv	Leukemia patients	95	26.1
Tahaei, 2011[102]	2006-2010	Tehran	Clinical: hospital & health care centers	CS	Conv	HBV patients	264	4.5
Tahaei, 2012[103]	2009-2012	Tehran	Clinical: hospital & health care centers	CS	Conv	Endoscopy patients	219	0.0
Talebi-Taher, 2010[104]	NS	Tehran	Clinical: hospital & health care centers	CS	Conv	Viral hepatitis patients	252	34.9
Valizadeh, 2012[105]	2006-2011	Azarbaijan	Clinical: hospital & health care centers	CS	Conv	Patients with hepatocellular cancer	60	0.0
Yeganeh, 2015[106]	2009-2011	Tehran	Clinical: hospital & health care centers	CS	SRS	Orthopedic surgeries	320	3.2

[a]Abbreviations: ALT, Alanine transaminase; CC, case-control; Conv, convenience; CS, cross-sectional; ESRD, end-stage renal disease; HBV, hepatitis B virus; NHL, Non-Hodgkin lymphoma; NS, not specified; SRS, simple random sampling.

[b]The decimal places of the prevalence figures are as reported in the original reports, but prevalence figures with more than one decimal places were rounded to one decimal place, with the exception of those below 0.1%

Table S4. Studies reporting hepatitis C virus (HCV) ribonucleic acid (RNA) prevalence in Iran.

Author, year (citation)	Year(s) of data collection	City or governorate	Study site	Population	Sample size	Sample tested for RNA	RNA prevalence among anti-HCV+	RNA prevalence among whole sample
Abdollahi, 2008[107]	2003	NS	Hemophilia units	Hemophilia patients	174	145	80.2%	
Alavi, 2005[108]	2002	Tehran	Clinical: hospital & health care centers	Thalassemia patients	110	13	84.6%	
Alavi, 2010[109]	2002-06	Ahvaz	Clinical: hospital & health care centers	Hospitalized PWID	333	333		30.9%
Aledavood, 2014[58]	2007-09	Mashhad	Research center	Non-Hodgkin's B-cell lymphoma patients	128	1		0.78%
Amini, 2005[110]	NS	Tehran	NS	Thalassemia patients	31	31		20.6%
Amini, 2005[110]	NS	Tehran	NS	PWID	34	34		19.4%
Amini, 2005[110]	NS	Tehran	NS	Blood donors	100	100		0%
Ansari, 2008[111]	1996-01	Tehran	Clinical: hospital & health care centers	Children with leukemia	408	408		2.0%
Arababadi, 2008[112]	2007	Kerman	NS	Thalassemia patients	60	60		45%
Ardakani, 2009[59]	2001	Tehran	Clinical: hospital & health care centers	Rheumatoid arthritis patients	200	5		2%
Assarehzadegan, 2012[113]	2008-09	Ahvaz	Research center	Hemophilia patients	87	47	89.3%	48.3%
Azarbahra, 2014[114]	NS	Shahrekord	Laboratory, blood transfusion center	Hepatitis B patients	350	350		0.57%
Azarkeivan, 2011[115]	2008	Tehran	Clinical: hospital & health care centers	Thalassemia patients	695	170	66%	
Boroujerdnia, 2009[116]	2006-07	Khuzestan	Clinical: hospital & health care centers	Thalassemia patients	206	58	79.3%	22.3%
Broumand, 2002[117]	NS	Tehran	Clinical: hospital & health care centers	Hemodialysis patients	548	105	48.6%	
Davarpanah, 2013[118]	2006-07	Shiraz	Counseling centers	HIV infected individuals	226	59		26.1%

Author, year (citation)	Year(s) of data collection	City or governorate	Study site	Population	Sample size	Sample tested for RNA	RNA prevalence among anti-HCV+	RNA prevalence among whole sample
Doosti, 2009[119]	2003-04	Shahrekord	Blood transfusion center	Blood donors	11,200	76	61.8%	
Esmaeli, 2004[120]	2003	Babol	Clinical: hospital & health care centers	Kids receiving blood products before 1996	100	2	50%	2%
Faranoush, 2006[121]	2002	Mixed (Semnan, Damaghan and Garmsar)	Clinical: hospital & health care centers	Thalassemia patients	63	25	60%	23.8%
Farshadpour, 2010[122]	2007-08	Ahvaz	Blood transfusion center	Blood donors	2,376	55		1.8%
Ghafourian-Boroujerdnia, 2009[123]	2005	Khuzestan	Clinical: hospital & health care centers	Thalassemia patients	206	206		22.3%
Ghafourian-Boroujerdnia, 2013[70]	2009-10	Ahvaz	Research center	Sickle cell anemia patients	47	42	89.3%	
Ghane, 2012[124]	2010	Mixed (Mazandaran and Guilan)	Clinical: hospital & health care centers	Thalassemia patients	245	36		11.4%
Hajiani, 2006[73]	2003-04	Tehran	Clinical: hospital & health care centers	Endoscopic/ colonoscopy patients	456	5		0.66%
Hajiani, 2006[73]	2003-04	Tehran	NS	Healthy adult	500	6		1.2%
Hassanshahi, 2011[125]	2006-07	Kerman	Clinical: hospital & health care centers	Hemodialysis patients	203	203		31.5%
Hassanshahi, 2011[125]	2006-07	Kerman	Clinical: hospital & health care centers	Thalassemia patients	181	181		44.7%
Joukar, 2011[126]	2009	Guilan	Clinical: hospital & health care centers	Hemodialysis patients	514	61	50.8%	11.9%
Kalantari, 2011[127]	2008-10	Isfahan	Clinical: hospital & health care centers	Hemophilia patients	495	459	70.1%	
Kalantari, 2011[127]	2008-10	Isfahan	Clinical: hospital & health care centers	Thalassemia patients	50	50	62%	
Kalantari, 2014[128]	2010	Birjand	Clinical: hospital & health care centers	Hemodialysis patients	41	41		2.4%

Author, year (citation)	Year(s) of data collection	City or governorate	Study site	Population	Sample size	Sample tested for RNA	RNA prevalence among anti-HCV+	RNA prevalence among whole sample
Karim, 2008[78]	2003-05	Ahvaz	Clinical: hospital & health care centers	Lupus patients	124	6		2.4%
Karim, 2008[78]	NS	Ahvaz	Blood transfusion center	Blood donors	125	3		0.8%
Kashef, 2008[129]	NS	Tabriz	Hospital	Thalassemia patients	131	131		5.3%
Khedmat, 2007[130]	2005-06	Tehran	Blood transfusion center	Blood donors	1,014	1,014		0.19%
Makhlough, 2008[131]	2006	Sari and Ghaemshahr, Mazandaran	Clinical: hospital & health care centers	Hemodialysis patients	186	39	11.3%	
Mansoori, 2003[132]	1998-00	Tehran	Clinical: hospital & health care centers	HIV patients	44	15	80%	
Metanat, 2006[33]	2004	Zahedan	Clinical: hospital & health care centers	Diabetes patients	505	6	16.7%	
Mohammad-Alizadeh, 2012[133]	2004-11	Tehran	Clinical: hospital & health care centers	Cancer patients	283	283		15.2%
Monsour-Ghanaei, 2007[134]	2003	Guilan	Nursing home	Residents of nursing home	383	9	55.6%	1.3%
Motamed, 2009[135]	1985-2006	NS	NS	Primary antibody deficient patients	62	62		1.6%
Mousavi, 2002[136]	NS	NS	NS	Thalassemia patients	81	22	77.3%	21%
Naghavi, 2007[137]	2005	Isfahan	Clinical: hospital & health care centers	Thalassemia patients	53	53		58.5%
Naghavi, 2007[137]	2005	Isfahan	Clinical: hospital & health care centers	Hemophilia patients	50	50		60%
Pourmand, 2007[85]	2002-04	Isfahan	Clinical: hospital & health care centers	ESRD patients undergoing renal transplantation	142	1	0%	
Samimi-Rad, 2007[138]	2004	Markazi	Clinical: hospital & health care centers	Hemophilia patients	76	34	68%	
Samimi-Rad, 2007[138]	2004	Markazi	Clinical: hospital & health care centers	Thalassemia patients	98	5	40%	

Author, year (citation)	Year(s) of data collection	City or governorate	Study site	Population	Sample size	Sample tested for RNA	RNA prevalence among anti-HCV+	RNA prevalence among whole sample
Samimi-rad, 2008[139]	2005	Markazi	Hemodialysis units	Hemodialysis patients	204	14		4.4%
Shamshiraz, 2004[140]	NS	Tehran	Clinical: hospital & health care centers	Hemodialysis patients	593	NS		8.6%
Somi, 2007[141]	2006	Tabriz	Clinical: hospital & health care centers	Hemodialysis patients	462	69		10%
Zamani, 2013[142]	2008-11	Mazandaran	Clinical: hospital & health care centers	General population	6,145	5		0.05%
Ziaee, 2007[143]	NS	South Khorassan	Hemophilia units	Hemophilia patients	80	21	80%	
Ziaei, 2005[144]	2000	Khorassan	Clinical: hospital & health care centers	Hemophilia patients	80	44	56.8%	31.3%
Ziyaeyan, 2012[145]	2009-10	Shiraz	Clinical: hospital & health care centers	Newborn babies to HCV positive mothers	29	0		0%

[a]Abbreviations: ESRD, end-stage renal disease; NS, not specified; PWID, people who inject drugs.

Fig S3. Forest plots presenting results of the meta-analyses of hepatitis C virus (HCV) prevalence in Iran among: A) general population (populations at low risk), B) populations at high risk, C) populations at intermediate risk, and D) special clinical populations.

A)

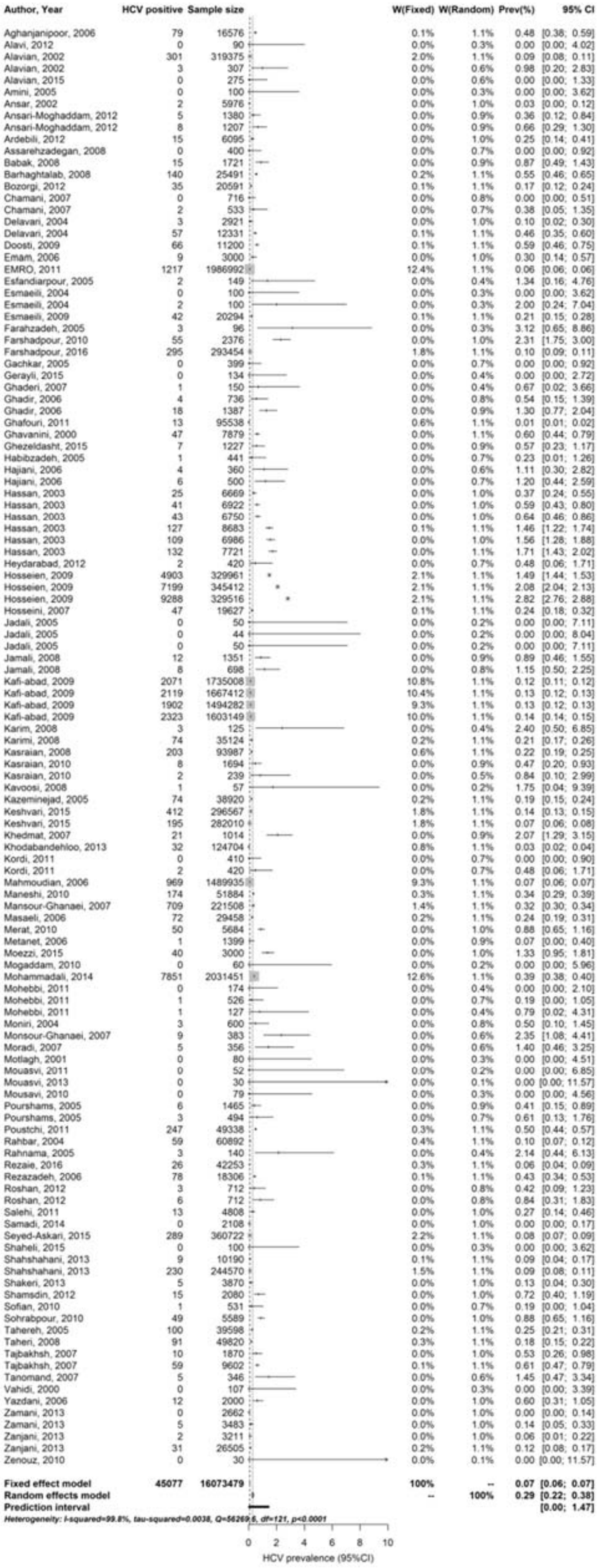

B)

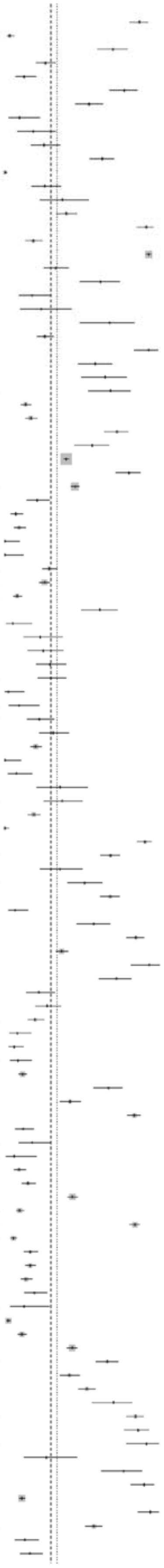

Author, Year	HCV positive	Sample size	W(Fixed)	W(Random)	Prev(%)	95% CI
Abdollahi, 2008	145	174	0.3%	0.5%	83.33	[76.95; 88.54]
Aghakhani, 2009	9	289	0.5%	0.5%	3.11	[1.43; 5.83]
Aghakhani, 2009	71	106	0.2%	0.5%	66.98	[57.18; 75.81]
Akbari, 2011	50	200	0.4%	0.5%	25.00	[19.16; 31.60]
Alavi, 2005	13	110	0.2%	0.5%	11.82	[6.45; 19.36]
Alavi, 2007	77	104	0.2%	0.5%	74.04	[64.52; 82.14]
Alavi, 2009	74	142	0.3%	0.5%	52.11	[43.58; 60.56]
Alavi, 2012	4	44	0.1%	0.5%	9.09	[2.53; 21.67]
Alavi, 2012	8	46	0.1%	0.5%	17.39	[7.82; 31.42]
Alavia, 2003	23	95	0.2%	0.5%	24.21	[16.01; 34.08]
Alavian, 2003	106	176	0.3%	0.5%	60.23	[52.59; 67.51]
Alavian, 2015	0	274	0.5%	0.5%	0.00	[0.00; 1.34]
Ali, 2008	23	93	0.2%	0.5%	24.73	[16.37; 34.76]
Alipour, 2013	15	42	0.1%	0.5%	35.71	[21.55; 51.97]
Alipour, 2013	86	226	0.4%	0.5%	38.05	[31.70; 44.73]
Alipour, 2013	147	168	0.3%	0.5%	87.50	[81.53; 92.09]
Alipour, 2013	38	215	0.4%	0.5%	17.67	[12.82; 23.44]
Alipour, 2013	1095	1230	2.2%	0.5%	89.02	[87.14; 90.72]
Alizadeh, 2005	47	149	0.3%	0.5%	31.54	[24.18; 39.65]
Alizadeh, 2006	39	66	0.1%	0.5%	59.09	[46.29; 71.05]
Ameli, 2008	11	65	0.1%	0.5%	16.92	[8.76; 28.27]
Amini, 2005	7	31	0.1%	0.5%	22.58	[9.59; 41.10]
Amini, 2005	22	34	0.1%	0.5%	64.71	[46.49; 80.25]
Amiri, 2005	74	298	0.5%	0.5%	24.83	[20.03; 30.14]
Amiri, 2007	72	81	0.1%	0.5%	88.89	[79.95; 94.79]
Ansar, 2002	52	93	0.2%	0.5%	55.91	[45.24; 66.20]
Ansar, 2002	31	50	0.1%	0.5%	62.00	[47.17; 75.35]
Ansar, 2002	36	55	0.1%	0.5%	65.45	[51.42; 77.76]
Ansari, 2007	52	406	0.7%	0.5%	12.81	[9.72; 16.45]
Ansari, 2007	64	400	0.7%	0.5%	16.00	[12.55; 19.97]
Asl, 2013	104	150	0.3%	0.5%	69.33	[61.29; 76.59]
Assarehzadegan, 2012	47	87	0.2%	0.5%	54.02	[43.00; 64.77]
Ataei, 2010	1248	3284	5.9%	0.5%	38.00	[36.34; 39.69]
Ataei, 2010	100	130	0.2%	0.5%	76.92	[68.72; 83.86]
Ataei, 2011	644	1485	2.7%	0.5%	43.37	[40.83; 45.93]
Ataei, 2011	27	136	0.2%	0.5%	19.85	[13.51; 27.55]
Ataei, 2012	13	193	0.3%	0.5%	6.74	[3.63; 11.24]
Ataei, 2012	24	270	0.5%	0.5%	8.89	[5.78; 12.94]
Azarkar, 2009	0	38	0.1%	0.5%	0.00	[0.00; 9.25]
Azarkar, 2009	0	30	0.1%	0.5%	0.00	[0.00; 11.57]
Azarkeivan, 2010	109	395	0.7%	0.5%	27.59	[23.24; 32.29]
Azarkeivan, 2011	170	695	1.3%	0.5%	24.46	[21.31; 27.83]
Azarkeivan, 2012	30	395	0.7%	0.5%	7.59	[5.18; 10.66]
Babamahmoodi, 2012	47	80	0.1%	0.5%	58.75	[47.18; 69.65]
Barak, 2003	2	40	0.1%	0.5%	5.00	[0.61; 16.92]
Basiratnia, 2010	11	50	0.1%	0.5%	22.00	[11.53; 35.96]
Basiratnia, 2010	15	63	0.1%	0.5%	23.81	[13.98; 36.21]
Boroujerdnia, 2009	27	97	0.2%	0.5%	27.84	[19.21; 37.86]
Boroujerdnia, 2009	31	109	0.2%	0.5%	28.44	[20.21; 37.88]
Bozorghi, 2006	1	44	0.1%	0.5%	2.27	[0.06; 12.02]
Bozorghi, 2006	4	45	0.1%	0.5%	8.89	[2.48; 21.22]
Bozorgi, 2008	21	99	0.2%	0.5%	21.21	[13.64; 30.58]
Bozorgi, 2008	31	104	0.2%	0.5%	29.81	[21.23; 39.57]
Broumand, 2002	105	548	1.0%	0.5%	19.16	[15.95; 22.71]
Company, 2007	0	35	0.1%	0.5%	0.00	[0.00; 10.00]
Company, 2007	4	57	0.1%	0.5%	7.02	[1.95; 17.00]
Company, 2007	13	38	0.1%	0.5%	34.21	[19.63; 51.35]
Company, 2007	23	65	0.1%	0.5%	35.38	[23.92; 48.23]
Dadgaran, 2005	70	393	0.7%	0.5%	17.81	[14.16; 21.96]
Dadmanesh, 2015	0	138	0.3%	0.5%	0.00	[0.00; 2.64]
Davarpanah, 2013	196	226	0.4%	0.5%	86.73	[81.60; 90.86]
Davoodian, 2009	163	249	0.5%	0.5%	65.46	[59.20; 71.35]
Eghbalian, 2000	18	53	0.1%	0.5%	33.96	[21.52; 48.27]
Esfahani, 2014	44	89	0.2%	0.5%	49.44	[38.67; 60.25]
Eskandarieh, 2013	168	258	0.5%	0.5%	65.12	[58.96; 70.92]
Eslamifar, 2007	5	77	0.1%	0.5%	6.49	[2.14; 14.51]
Etminani-Esfahani, 2012	54	98	0.2%	0.5%	55.10	[44.72; 65.17]
Faramarzi, 2013	180	222	0.4%	0.5%	81.08	[75.30; 86.01]
Faranoush, 2006	222	630	1.1%	0.5%	35.24	[31.51; 39.11]
Farhoudi, 2016	50	56	0.1%	0.5%	89.29	[78.12; 95.97]
Ghaderi, 1996	62	90	0.2%	0.5%	68.89	[58.26; 78.23]
Ghadir, 2009 (1053)	19	90	0.2%	0.5%	21.11	[13.21; 30.99]
Ghafoorian-Broujerdnia, 2006	32	122	0.2%	0.5%	26.23	[18.68; 34.96]
Ghane, 2012	46	245	0.4%	0.5%	18.78	[14.09; 24.24]
Haghazali, 2011	6	76	0.1%	0.5%	7.89	[2.95; 16.40]
Hamissi, 2011	7	120	0.2%	0.5%	5.83	[2.38; 11.65]
Hamissi, 2011	6	75	0.1%	0.5%	8.00	[2.99; 16.60]
Hariri, 2006	67	616	1.1%	0.5%	10.88	[8.53; 13.61]
Hariri, 2006	77	120	0.2%	0.5%	64.17	[54.90; 72.71]
Honarvar, 2013	94	233	0.4%	0.5%	40.34	[33.99; 46.95]
Hosseini, 2010	334	417	0.8%	0.5%	80.10	[75.94; 83.82]
Imani, 2008	15	133	0.2%	0.5%	11.28	[6.45; 17.92]
Ismail, 2005	11	65	0.1%	0.5%	16.92	[8.76; 28.27]
Jahromi, 2007	2	34	0.1%	0.5%	5.88	[0.72; 19.68]
Joukar, 2011	20	228	0.4%	0.5%	8.77	[5.44; 13.22]
Joukar, 2011	41	286	0.5%	0.5%	14.34	[10.49; 18.94]
Kaffashian, 2011	398	951	1.7%	0.5%	41.85	[38.69; 45.06]
Kalantari, 2011	50	545	1.0%	0.5%	9.17	[6.89; 11.92]
Kalantari, 2011	495	615	1.1%	0.5%	80.49	[77.13; 83.55]
Kalantari, 2014	26	499	0.9%	0.5%	5.21	[3.43; 7.54]
Karimi, 2001	44	281	0.5%	0.5%	15.66	[11.61; 20.44]
Karimi, 2001	73	466	0.8%	0.5%	15.67	[12.48; 19.29]
Karimi, 2002	48	367	0.7%	0.5%	13.08	[9.80; 16.96]
Kashef, 2008	24	131	0.2%	0.5%	18.32	[12.11; 26.02]
Kasraiyan, 2003	4	34	0.1%	0.5%	11.76	[3.30; 27.45]
Kassaian, 2011	17	800	1.4%	0.5%	2.12	[1.24; 3.38]
Kassaian, 2011	60	570	1.0%	0.5%	10.53	[8.13; 13.34]
Kassaian, 2012	392	943	1.7%	0.5%	41.57	[38.40; 44.79]
Keramat, 2011	126	199	0.4%	0.5%	63.32	[56.21; 70.02]
Keshvari, 2014	103	257	0.5%	0.5%	40.08	[34.04; 46.35]
Khani, 2003	176	346	0.6%	0.5%	50.87	[45.47; 56.25]
Kheirandish, 2009	41	61	0.1%	0.5%	67.21	[54.00; 78.69]
Kheirandish, 2009	185	229	0.4%	0.5%	80.79	[75.08; 85.68]
Kheirandish, 2009	85	103	0.2%	0.5%	82.52	[73.79; 89.30]
Kheirandish, 2009	43	49	0.1%	0.5%	87.76	[75.23; 95.37]
Khodadi, 2003	8	31	0.1%	0.5%	25.81	[11.86; 44.61]
Khorvash, 2008	39	53	0.1%	0.5%	73.58	[59.67; 84.74]
Khosravi, 2010	87	101	0.2%	0.5%	86.14	[77.84; 92.21]
Kiakalayeh, 2013	117	1113	2.0%	0.5%	10.51	[8.77; 12.46]
Lak, 2000	90	100	0.2%	0.5%	90.00	[82.38; 95.10]
Lak, 2000	212	385	0.7%	0.5%	55.06	[49.94; 60.11]
Langarodi, 2011	11	90	0.2%	0.5%	12.22	[6.26; 20.82]
Langarodi, 2011	18	116	0.2%	0.5%	15.52	[9.46; 23.41]

Study	Events	Total	Weight (fixed)	Weight (random)	Prevalence	95% CI
Mahdaviani, 2008	7	97	0.2%	0.5%	7.22	[2.95; 14.30]
Mahdaviani, 2008	25	68	0.1%	0.5%	36.76	[25.39; 49.33]
Mahdavimazdeh, 2009	227	2403	4.3%	0.5%	9.45	[8.31; 10.69]
Mak, 2001	27	86	0.2%	0.5%	31.40	[21.81; 42.30]
Makhlough, 2008	39	186	0.3%	0.5%	20.97	[15.36; 27.53]
Mansoori, 2003	29	39	0.1%	0.5%	74.36	[57.87; 86.96]
Mansour-Ghanaei, 2002	72	101	0.2%	0.5%	71.29	[61.43; 79.85]
Mansour-Ghanaei, 2009	17	163	0.3%	0.5%	10.43	[6.19; 16.17]
Mansour-Ghanaei, 2009	186	370	0.7%	0.5%	50.27	[45.06; 55.48]
Mashayekhi, 2011	3	100	0.2%	0.5%	3.00	[0.62; 8.52]
Mehrjerdi, 2014	56	209	0.4%	0.5%	26.79	[20.92; 33.34]
Meidani, 2009	39	150	0.3%	0.5%	26.00	[19.19; 33.79]
Mirahmadizadeh, 2004	149	186	0.3%	0.5%	80.11	[73.64; 85.59]
Mirahmadizadeh, 2009	406	936	1.7%	0.5%	43.38	[40.17; 46.62]
Mirmomen, 2006	141	732	1.3%	0.5%	19.26	[16.47; 22.31]
Mir-Nasseri, 2005	21	42	0.1%	0.5%	50.00	[34.19; 65.81]
Mir-Nasseri, 2005	287	425	0.8%	0.5%	67.53	[62.85; 71.96]
Mir-Nasseri, 2008	21	54	0.1%	0.5%	38.89	[25.92; 53.12]
Mir-Nasseri, 2008	287	464	0.8%	0.5%	61.85	[57.26; 66.29]
Mir-Nasseri, 2011	359	518	0.9%	0.5%	69.31	[65.13; 73.25]
Mobini, 2010	38	77	0.1%	0.5%	49.35	[37.76; 61.00]
Mohammadi, 2009	8	33	0.1%	0.5%	24.24	[11.09; 42.26]
Mohammadi, 2009	274	358	0.6%	0.5%	76.54	[71.80; 80.83]
Momen-Heravi, 2012	142	300	0.5%	0.5%	47.33	[41.57; 53.15]
Mousavi, 2002	22	81	0.1%	0.5%	27.16	[17.87; 38.19]
Mousavi, 2014	2	47	0.1%	0.5%	4.26	[0.52; 14.54]
Mousavian, 2011	802	1095	2.0%	0.5%	73.24	[70.51; 75.84]
Najafi, 2001	8	49	0.1%	0.5%	16.33	[7.32; 29.66]
Najafi, 2001	10	51	0.1%	0.5%	19.61	[9.82; 33.12]
Nani, 2007	125	553	1.0%	0.5%	22.60	[19.18; 26.32]
Pourahmad, 2007	308	401	0.7%	0.5%	76.81	[72.36; 80.85]
Rahbar, 2004	60	101	0.2%	0.5%	59.41	[49.18; 69.07]
Rahimi-Movaghar, 2010	293	859	1.6%	0.5%	34.11	[30.94; 37.39]
Rahimi-Movaghar, 2010	16	36	0.1%	0.5%	44.44	[27.94; 61.90]
Ramezani, 2008	90	171	0.3%	0.5%	52.63	[44.87; 60.30]
Ramezani, 2009	63	92	0.2%	0.5%	68.48	[57.96; 77.77]
Ramzani, 2014	56	100	0.2%	0.5%	56.00	[45.72; 65.92]
Rostami, 2013	15	1704	3.1%	0.5%	0.88	[0.49; 1.45]
Rostami, 2013	38	2259	4.1%	0.5%	1.68	[1.19; 2.30]
Rostami-Jalilian, 2006	26	76	0.1%	0.5%	34.21	[23.71; 45.99]
Rostami-Jalilian, 2006	33	72	0.1%	0.5%	45.83	[34.02; 58.00]
Sabour, 2003	37	140	0.3%	0.5%	26.43	[19.34; 34.54]
Saleh, 2011	57	94	0.2%	0.5%	60.64	[50.02; 70.56]
Salehi, 2014	4	40	0.1%	0.5%	10.00	[2.79; 23.66]
Salehi, 2015	179	1327	2.4%	0.5%	13.49	[11.70; 15.45]
Sali, 2013	123	173	0.3%	0.5%	71.10	[63.73; 77.73]
Samak, 2012	19	142	0.3%	0.5%	13.38	[8.25; 20.10]
Samarbaf-Zadeh, 2015	39	430	0.8%	0.5%	9.07	[6.53; 12.19]
Samimi-Rad, 2007	2	50	0.1%	0.5%	4.00	[0.49; 13.71]
Samimi-Rad, 2007	3	48	0.1%	0.5%	6.25	[1.31; 17.20]
Samimi-Rad, 2007	33	76	0.1%	0.5%	43.42	[32.08; 55.29]
Samimi-Rad, 2008	1	101	0.2%	0.5%	0.99	[0.03; 5.39]
Samimi-Rad, 2008	10	103	0.2%	0.5%	9.71	[4.75; 17.13]
Sanei, 2004	49	364	0.7%	0.5%	13.46	[10.13; 17.40]
Sani, 2012	44	62	0.1%	0.5%	70.97	[58.05; 81.80]
Sarkari, 2012	3	49	0.1%	0.5%	6.12	[1.28; 16.87]
Sarkari, 2012	67	158	0.3%	0.5%	42.41	[34.59; 50.51]
SeyedAlinaghi, 2011	2	29	0.1%	0.5%	6.90	[0.85; 22.77]
SeyedAlinaghi, 2011	133	172	0.3%	0.5%	77.33	[70.33; 83.35]
Seyrafian, 2006	16	556	1.0%	0.5%	2.88	[1.65; 4.63]
Shahshahani, 2006	8	85	0.2%	0.5%	9.41	[4.15; 17.71]
Shahshahani, 2006	36	74	0.1%	0.5%	48.65	[36.85; 60.56]
Sharif, 2009	24	200	0.4%	0.5%	12.00	[7.84; 17.33]
Sharifi-Mood, 2006	7	31	0.1%	0.5%	22.58	[9.59; 41.10]
Sharifi-Mood, 2006	9	47	0.1%	0.5%	19.15	[9.15; 33.26]
Sharifi-Mood, 2006	23	74	0.1%	0.5%	31.08	[20.83; 42.90]
Sharifi-Mood, 2007	24	81	0.1%	0.5%	29.63	[19.99; 40.81]
Sheikholeslami, 09	38	43	0.1%	0.5%	88.37	[74.92; 96.11]
Siavash, 2008	15	381	0.7%	0.5%	3.94	[2.22; 6.41]
Siavash, 2008	20	506	0.9%	0.5%	3.95	[2.43; 6.04]
Sofian, 2012	91	153	0.3%	0.5%	59.48	[51.25; 67.33]
Somi, 2007	69	462	0.8%	0.5%	14.94	[11.81; 18.52]
Somi, 2014	37	455	0.8%	0.5%	8.13	[5.79; 11.03]
Soudbakhsh, 2008	23	26	0.0%	0.4%	88.46	[69.85; 97.55]
Taremi, 2005	66	324	0.6%	0.5%	20.37	[16.12; 25.17]
Tayeri, 2008	80	106	0.2%	0.5%	75.47	[66.16; 83.31]
Taziki, 2008	61	497	0.9%	0.5%	12.27	[9.52; 15.49]
Taziki, 2008	64	348	0.6%	0.5%	18.39	[14.46; 22.87]
Toosi, 2007	11	130	0.2%	0.5%	8.46	[4.30; 14.64]
Torabi, 2005	6	84	0.2%	0.5%	7.14	[2.67; 14.90]
Torabi, 2006	72	130	0.2%	0.5%	55.38	[46.42; 64.10]
Vahidi, 2000	24	107	0.2%	0.5%	22.43	[14.93; 31.51]
Valizadeh, 2013	3	50	0.1%	0.5%	6.00	[1.25; 16.55]
Valizadeh, 2015	0	32	0.1%	0.5%	0.00	[0.00; 10.89]
Yazdani, 2012	232	350	0.6%	0.5%	66.29	[61.07; 71.22]
Zadeh, 2007	25	70	0.1%	0.5%	35.71	[24.61; 48.07]
Zahedi, 2004	43	97	0.2%	0.5%	44.33	[34.24; 54.77]
Zahedi, 2012	7	228	0.4%	0.5%	3.07	[1.24; 6.22]
Zali, 2001	182	402	0.7%	0.5%	45.27	[40.33; 50.28]
Zamani, 2007	105	202	0.4%	0.5%	51.98	[44.86; 59.04]
Zamani, 2010	71	117	0.2%	0.5%	60.68	[51.23; 69.59]
Ziaee, 2005	44	80	0.1%	0.5%	55.00	[43.47; 66.15]
Ziaee, 2007	21	80	0.1%	0.5%	26.25	[17.04; 37.29]
Ziaee, 2013	0	41	0.1%	0.5%	0.00	[0.00; 8.60]
Ziaee, 2015	22	108	0.2%	0.5%	20.37	[13.23; 29.20]
**Fixed effect model**	**17534**	**55257**	**100%**	**--**	**28.41**	**[28.03; 28.79]**
**Random effects model**			**--**	**100%**	**32.10**	**[28.13; 36.21]**
**Prediction interval**						**[0.00; 88.50]**

Heterogeneity: I-squared=99%, tau-squared=0.3865, Q=21272.1, df=207, p<0.0001

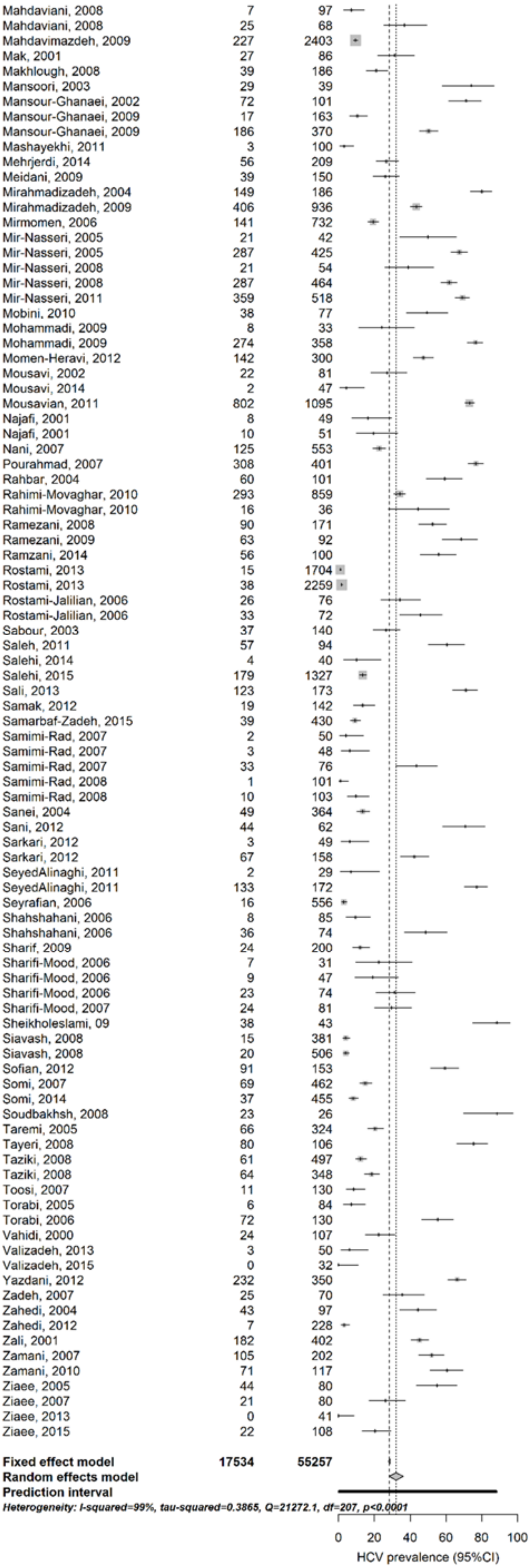

C)

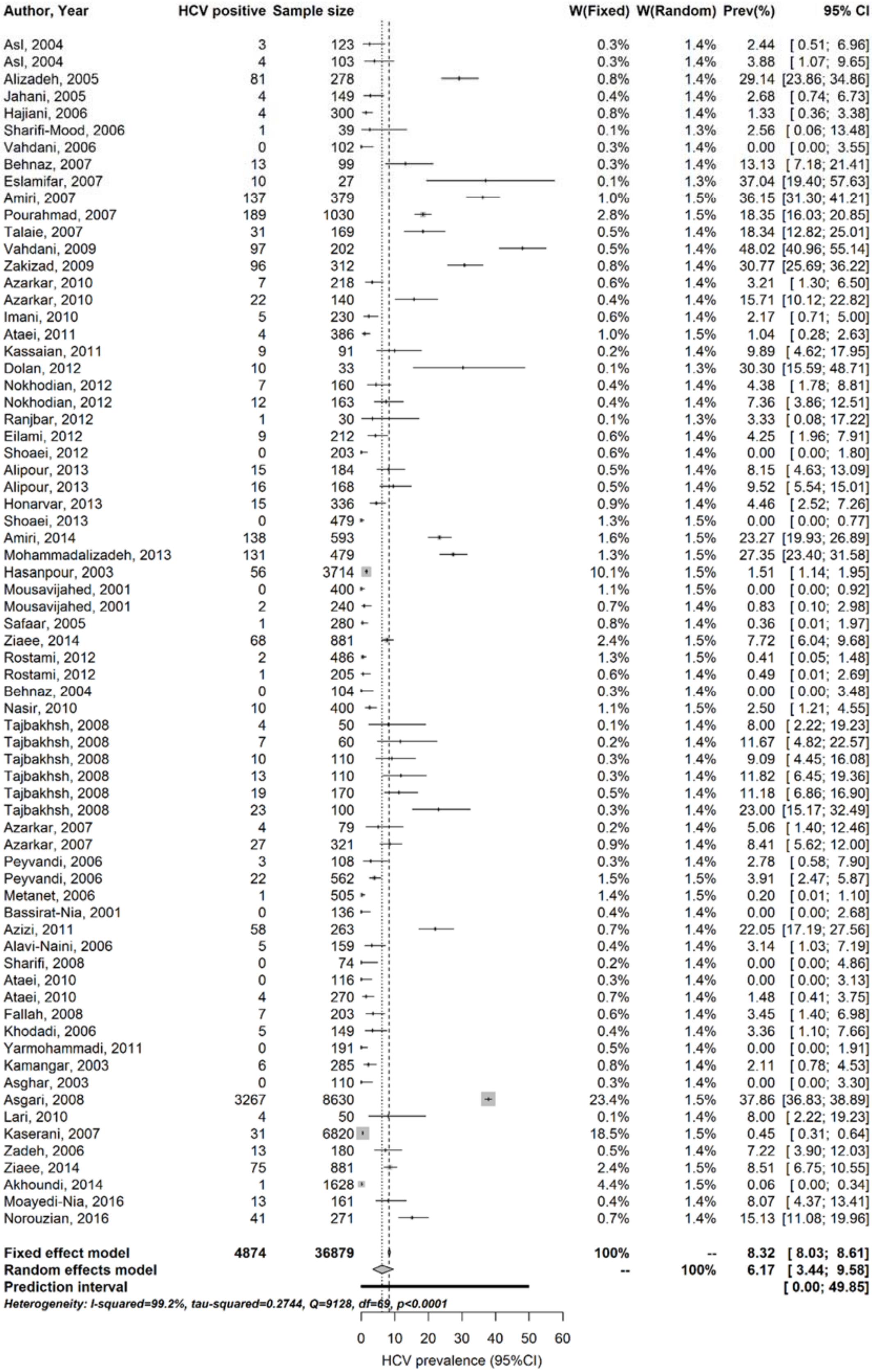
Author, Year HCV positive Sample size W(Fixed) W(Random) Prev(%) 95% CI
Asl, 2004 3 123 0.3% 1.4% 2.44 [0.51; 6.96]
Asl, 2004 4 103 0.3% 1.4% 3.88 [1.07; 9.65]
Alizadeh, 2005 81 278 0.8% 1.4% 29.14 [23.86; 34.86]
Jahani, 2005 4 149 0.4% 1.4% 2.68 [0.74; 6.73]
Hajiani, 2006 4 300 0.8% 1.4% 1.33 [0.36; 3.38]
Sharifi-Mood, 2006 1 39 0.1% 1.3% 2.56 [0.06; 13.48]
Vahdani, 2006 0 102 0.3% 1.4% 0.00 [0.00; 3.55]
Behnaz, 2007 13 99 0.3% 1.4% 13.13 [7.18; 21.41]
Eslamifar, 2007 10 27 0.1% 1.3% 37.04 [19.40; 57.63]
Amiri, 2007 137 379 1.0% 1.5% 36.15 [31.30; 41.21]
Pourahmad, 2007 189 1030 2.8% 1.5% 18.35 [16.03; 20.85]
Talaie, 2007 31 169 0.5% 1.4% 18.34 [12.82; 25.01]
Vahdani, 2009 97 202 0.5% 1.4% 48.02 [40.96; 55.14]
Zakizad, 2009 96 312 0.8% 1.4% 30.77 [25.69; 36.22]
Azarkar, 2010 7 218 0.6% 1.4% 3.21 [1.30; 6.50]
Azarkar, 2010 22 140 0.4% 1.4% 15.71 [10.12; 22.82]
Imani, 2010 5 230 0.6% 1.4% 2.17 [0.71; 5.00]
Ataei, 2011 4 386 1.0% 1.5% 1.04 [0.28; 2.63]
Kassaian, 2011 9 91 0.2% 1.4% 9.89 [4.62; 17.95]
Dolan, 2012 10 33 0.1% 1.3% 30.30 [15.59; 48.71]
Nokhodian, 2012 7 160 0.4% 1.4% 4.38 [1.78; 8.81]
Nokhodian, 2012 12 163 0.4% 1.4% 7.36 [3.86; 12.51]
Ranjbar, 2012 1 30 0.1% 1.3% 3.33 [0.08; 17.22]
Eilami, 2012 9 212 0.6% 1.4% 4.25 [1.96; 7.91]
Shoaei, 2012 0 203 0.6% 1.4% 0.00 [0.00; 1.80]
Alipour, 2013 15 184 0.5% 1.4% 8.15 [4.63; 13.09]
Alipour, 2013 16 168 0.5% 1.4% 9.52 [5.54; 15.01]
Honarvar, 2013 15 336 0.9% 1.4% 4.46 [2.52; 7.26]
Shoaei, 2013 0 479 1.3% 1.5% 0.00 [0.00; 0.77]
Amiri, 2014 138 593 1.6% 1.5% 23.27 [19.93; 26.89]
Mohammadalizadeh, 2013 131 479 1.3% 1.5% 27.35 [23.40; 31.58]
Hasanpour, 2003 56 3714 10.1% 1.5% 1.51 [1.14; 1.95]
Mousavijahed, 2001 0 400 1.1% 1.5% 0.00 [0.00; 0.92]
Mousavijahed, 2001 2 240 0.7% 1.4% 0.83 [0.10; 2.98]
Safaar, 2005 1 280 0.8% 1.4% 0.36 [0.01; 1.97]
Ziaee, 2014 68 881 2.4% 1.5% 7.72 [6.04; 9.68]
Rostami, 2012 2 486 1.3% 1.5% 0.41 [0.05; 1.48]
Rostami, 2012 1 205 0.6% 1.4% 0.49 [0.01; 2.69]
Behnaz, 2004 0 104 0.3% 1.4% 0.00 [0.00; 3.48]
Nasir, 2010 10 400 1.1% 1.5% 2.50 [1.21; 4.55]
Tajbakhsh, 2008 4 50 0.1% 1.4% 8.00 [2.22; 19.23]
Tajbakhsh, 2008 7 60 0.2% 1.4% 11.67 [4.82; 22.57]
Tajbakhsh, 2008 10 110 0.3% 1.4% 9.09 [4.45; 16.08]
Tajbakhsh, 2008 13 110 0.3% 1.4% 11.82 [6.45; 19.36]
Tajbakhsh, 2008 19 170 0.5% 1.4% 11.18 [6.86; 16.90]
Tajbakhsh, 2008 23 100 0.3% 1.4% 23.00 [15.17; 32.49]
Azarkar, 2007 4 79 0.2% 1.4% 5.06 [1.40; 12.46]
Azarkar, 2007 27 321 0.9% 1.4% 8.41 [5.62; 12.00]
Peyvandi, 2006 3 108 0.3% 1.4% 2.78 [0.58; 7.90]
Peyvandi, 2006 22 562 1.5% 1.5% 3.91 [2.47; 5.87]
Metanet, 2006 1 505 1.4% 1.5% 0.20 [0.01; 1.10]
Bassirat-Nia, 2001 0 136 0.4% 1.4% 0.00 [0.00; 2.68]
Azizi, 2011 58 263 0.7% 1.4% 22.05 [17.19; 27.56]
Alavi-Naini, 2006 5 159 0.4% 1.4% 3.14 [1.03; 7.19]
Sharifi, 2008 0 74 0.2% 1.4% 0.00 [0.00; 4.86]
Ataei, 2010 0 116 0.3% 1.4% 0.00 [0.00; 3.13]
Ataei, 2010 4 270 0.7% 1.4% 1.48 [0.41; 3.75]
Fallah, 2008 7 203 0.6% 1.4% 3.45 [1.40; 6.98]
Khodadi, 2006 5 149 0.4% 1.4% 3.36 [1.10; 7.66]
Yarmohammadi, 2011 0 191 0.5% 1.4% 0.00 [0.00; 1.91]
Kamangar, 2003 6 285 0.8% 1.4% 2.11 [0.78; 4.53]
Asghar, 2003 0 110 0.3% 1.4% 0.00 [0.00; 3.30]
Asgari, 2008 3267 8630 23.4% 1.5% 37.86 [36.83; 38.89]
Lari, 2010 4 50 0.1% 1.4% 8.00 [2.22; 19.23]
Kaserani, 2007 31 6820 18.5% 1.5% 0.45 [0.31; 0.64]
Zadeh, 2006 13 180 0.5% 1.4% 7.22 [3.90; 12.03]
Ziaee, 2014 75 881 2.4% 1.5% 8.51 [6.75; 10.55]
Akhoundi, 2014 1 1628 4.4% 1.5% 0.06 [0.00; 0.34]
Moayedi-Nia, 2016 13 161 0.4% 1.4% 8.07 [4.37; 13.41]
Norouzian, 2016 41 271 0.7% 1.4% 15.13 [11.08; 19.96]
Fixed effect model 4874 36879 100% -- 8.32 [8.03; 8.61]
Random effects model -- 100% 6.17 [3.44; 9.58]
Prediction interval [0.00; 49.85]
Heterogeneity: I-squared=99.2%, tau-squared=0.2744, Q=9128, df=69, p<0.0001
0 10 20 30 40 50 60
HCV prevalence (95%CI)

D)

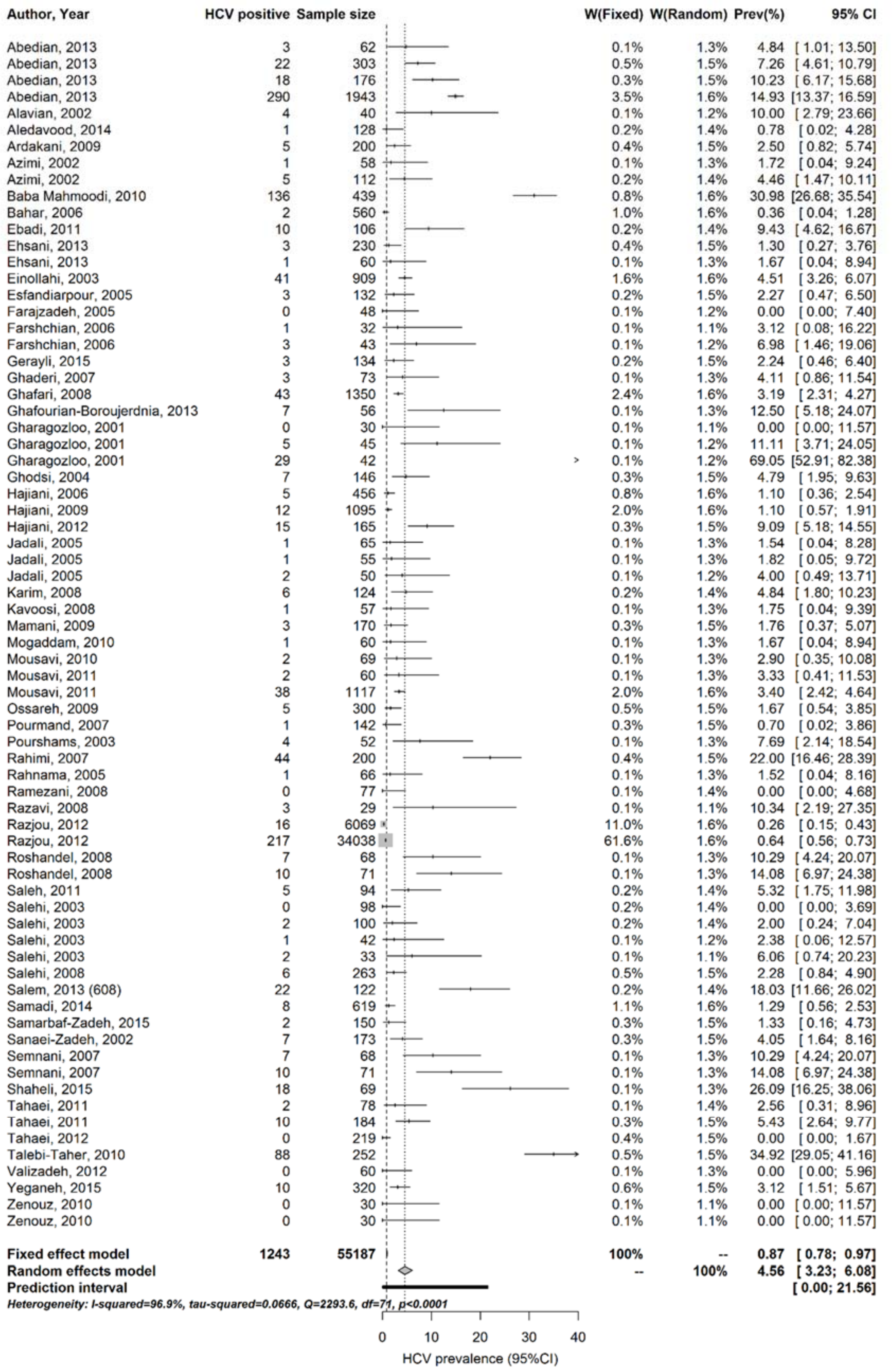

Author, Year HCV positive Sample size W(Fixed) W(Random) Prev(%) 95% CI
Abedian, 2013 3 62 0.1% 1.3% 4.84 [1.01; 13.50]
Abedian, 2013 22 303 0.5% 1.5% 7.26 [4.61; 10.79]
Abedian, 2013 18 176 0.3% 1.5% 10.23 [6.17; 15.68]
Abedian, 2013 290 1943 3.5% 1.6% 14.93 [13.37; 16.59]
Alavian, 2002 4 40 0.1% 1.2% 10.00 [2.79; 23.66]
Aledavood, 2014 1 128 0.2% 1.4% 0.78 [0.02; 4.28]
Ardakani, 2009 5 200 0.4% 1.5% 2.50 [0.82; 5.74]
Azimi, 2002 1 58 0.1% 1.3% 1.72 [0.04; 9.24]
Azimi, 2002 5 112 0.2% 1.4% 4.46 [1.47; 10.11]
Baba Mahmoodi, 2010 136 439 0.8% 1.6% 30.98 [26.68; 35.54]
Bahar, 2006 2 560 1.0% 1.6% 0.36 [0.04; 1.28]
Ebadi, 2011 10 106 0.2% 1.4% 9.43 [4.62; 16.67]
Ehsani, 2013 3 230 0.4% 1.5% 1.30 [0.27; 3.76]
Ehsani, 2013 1 60 0.1% 1.3% 1.67 [0.04; 8.94]
Einollahi, 2003 41 909 1.6% 1.6% 4.51 [3.26; 6.07]
Esfandiarpour, 2005 3 132 0.2% 1.5% 2.27 [0.47; 6.50]
Farajzadeh, 2005 0 48 0.1% 1.2% 0.00 [0.00; 7.40]
Farshchian, 2006 1 32 0.1% 1.1% 3.12 [0.08; 16.22]
Farshchian, 2006 3 43 0.1% 1.2% 6.98 [1.46; 19.06]
Gerayli, 2015 3 134 0.2% 1.5% 2.24 [0.46; 6.40]
Ghaderi, 2007 3 73 0.1% 1.3% 4.11 [0.86; 11.54]
Ghafari, 2008 43 1350 2.4% 1.6% 3.19 [2.31; 4.27]
Ghafourian-Boroujerdnia, 2013 7 56 0.1% 1.3% 12.50 [5.18; 24.07]
Gharagozloo, 2001 0 30 0.1% 1.1% 0.00 [0.00; 11.57]
Gharagozloo, 2001 5 45 0.1% 1.2% 11.11 [3.71; 24.05]
Gharagozloo, 2001 29 42 0.1% 1.2% 69.05 [52.91; 82.38]
Ghodsi, 2004 7 146 0.3% 1.5% 4.79 [1.95; 9.63]
Hajiani, 2006 5 456 0.8% 1.6% 1.10 [0.36; 2.54]
Hajiani, 2009 12 1095 2.0% 1.6% 1.10 [0.57; 1.91]
Hajiani, 2012 15 165 0.3% 1.5% 9.09 [5.18; 14.55]
Jadali, 2005 1 65 0.1% 1.3% 1.54 [0.04; 8.28]
Jadali, 2005 1 55 0.1% 1.3% 1.82 [0.05; 9.72]
Jadali, 2005 2 50 0.1% 1.2% 4.00 [0.49; 13.71]
Karim, 2008 6 124 0.2% 1.4% 4.84 [1.80; 10.23]
Kavoosi, 2008 1 57 0.1% 1.3% 1.75 [0.04; 9.39]
Mamani, 2009 3 170 0.3% 1.5% 1.76 [0.37; 5.07]
Mogaddam, 2010 1 60 0.1% 1.3% 1.67 [0.04; 8.94]
Mousavi, 2010 2 69 0.1% 1.3% 2.90 [0.35; 10.08]
Mousavi, 2011 2 60 0.1% 1.3% 3.33 [0.41; 11.53]
Mousavi, 2011 38 1117 2.0% 1.6% 3.40 [2.42; 4.64]
Ossareh, 2009 5 300 0.5% 1.5% 1.67 [0.54; 3.85]
Pourmand, 2007 1 142 0.3% 1.5% 0.70 [0.02; 3.86]
Pourshams, 2003 4 52 0.1% 1.3% 7.69 [2.14; 18.54]
Rahimi, 2007 44 200 0.4% 1.5% 22.00 [16.46; 28.39]
Rahnama, 2005 1 66 0.1% 1.3% 1.52 [0.04; 8.16]
Ramezani, 2008 0 77 0.1% 1.4% 0.00 [0.00; 4.68]
Razavi, 2008 3 29 0.1% 1.1% 10.34 [2.19; 27.35]
Razjou, 2012 16 6069 11.0% 1.6% 0.26 [0.15; 0.43]
Razjou, 2012 217 34038 61.6% 1.6% 0.64 [0.56; 0.73]
Roshandel, 2008 7 68 0.1% 1.3% 10.29 [4.24; 20.07]
Roshandel, 2008 10 71 0.1% 1.3% 14.08 [6.97; 24.38]
Saleh, 2011 5 94 0.2% 1.4% 5.32 [1.75; 11.98]
Salehi, 2003 0 98 0.2% 1.4% 0.00 [0.00; 3.69]
Salehi, 2003 2 100 0.2% 1.4% 2.00 [0.24; 7.04]
Salehi, 2003 1 42 0.1% 1.2% 2.38 [0.06; 12.57]
Salehi, 2003 2 33 0.1% 1.1% 6.06 [0.74; 20.23]
Salehi, 2008 6 263 0.5% 1.5% 2.28 [0.84; 4.90]
Salem, 2013 (608) 22 122 0.2% 1.4% 18.03 [11.66; 26.02]
Samadi, 2014 8 619 1.1% 1.6% 1.29 [0.56; 2.53]
Samarbaf-Zadeh, 2015 2 150 0.3% 1.5% 1.33 [0.16; 4.73]
Sanaei-Zadeh, 2002 7 173 0.3% 1.5% 4.05 [1.64; 8.16]
Semnani, 2007 7 68 0.1% 1.3% 10.29 [4.24; 20.07]
Semnani, 2007 10 71 0.1% 1.3% 14.08 [6.97; 24.38]
Shaheli, 2015 18 69 0.1% 1.3% 26.09 [16.25; 38.06]
Tahaei, 2011 2 78 0.1% 1.4% 2.56 [0.31; 8.96]
Tahaei, 2011 10 184 0.3% 1.5% 5.43 [2.64; 9.77]
Tahaei, 2012 0 219 0.4% 1.5% 0.00 [0.00; 1.67]
Talebi-Taher, 2010 88 252 0.5% 1.5% 34.92 [29.05; 41.16]
Valizadeh, 2012 0 60 0.1% 1.3% 0.00 [0.00; 5.96]
Yeganeh, 2015 10 320 0.6% 1.5% 3.12 [1.51; 5.67]
Zenouz, 2010 0 30 0.1% 1.1% 0.00 [0.00; 11.57]
Zenouz, 2010 0 30 0.1% 1.1% 0.00 [0.00; 11.57]
Fixed effect model 1243 55187 100% -- 0.87 [0.78; 0.97]
Random effects model -- 100% 4.56 [3.23; 6.08]
Prediction interval [0.00; 21.56]
Heterogeneity: I-squared=96.9%, tau-squared=0.0666, Q=2293.6, df=71, p<0.0001
0
10
20
30
40
HCV prevalence (95%CI)

Fig S4. Forest plots presenting results of the sub-population meta-analyses of hepatitis C virus (HCV) prevalence in Iran among: A) populations at high risk of healthcare-related exposures, B) people who inject drugs, C) populations with liver-related conditions, and D) other special clinical populations.

A)

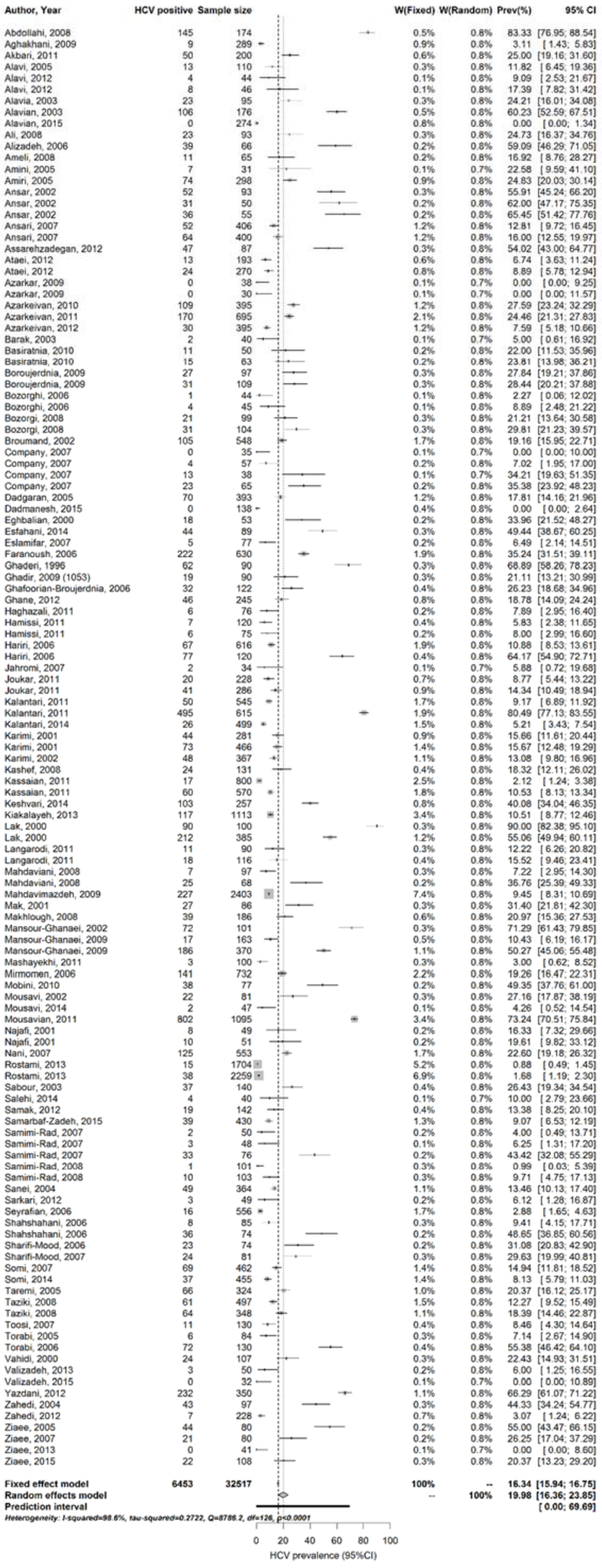

Author, Year HCV positive Sample size W(Fixed) W(Random) Prev(%) 95% CI
Abdollahi, 2008 145 174 0.5% 0.8% 83.33 [76.95; 88.54]
Aghakhani, 2009 9 289 0.9% 0.8% 3.11 [1.43; 5.83]
Akbari, 2011 50 200 0.6% 0.8% 25.00 [19.16; 31.60]
Alavi, 2005 13 110 0.3% 0.8% 11.82 [6.45; 19.36]
Alavi, 2012 4 44 0.1% 0.8% 9.09 [2.53; 21.67]
Alavi, 2012 8 46 0.1% 0.8% 17.39 [7.82; 31.42]
Alavia, 2003 23 95 0.3% 0.8% 24.21 [16.01; 34.08]
Alavian, 2003 106 176 0.5% 0.8% 60.23 [52.59; 67.51]
Alavian, 2015 0 274 0.8% 0.8% 0.00 [0.00; 1.34]
Ali, 2008 23 93 0.3% 0.8% 24.73 [16.37; 34.76]
Alizadeh, 2006 39 66 0.2% 0.8% 59.09 [46.29; 71.05]
Ameli, 2008 11 65 0.2% 0.8% 16.92 [8.76; 28.27]
Amini, 2005 7 31 0.1% 0.7% 22.58 [9.59; 41.10]
Amiri, 2005 74 298 0.9% 0.8% 24.83 [20.03; 30.14]
Ansar, 2002 52 93 0.3% 0.8% 55.91 [45.24; 66.20]
Ansar, 2002 31 50 0.2% 0.8% 62.00 [47.17; 75.35]
Ansar, 2002 36 55 0.2% 0.8% 65.45 [51.42; 77.76]
Ansari, 2007 52 406 1.2% 0.8% 12.81 [9.72; 16.45]
Ansari, 2007 64 400 1.2% 0.8% 16.00 [12.55; 19.97]
Assarehzadegan, 2012 47 87 0.3% 0.8% 54.02 [43.00; 64.77]
Ataei, 2012 13 193 0.6% 0.8% 6.74 [3.63; 11.24]
Ataei, 2012 24 270 0.8% 0.8% 8.89 [5.78; 12.94]
Azarkar, 2009 0 38 0.1% 0.7% 0.00 [0.00; 9.25]
Azarkar, 2009 0 30 0.1% 0.7% 0.00 [0.00; 11.57]
Azarkeivan, 2010 109 395 1.2% 0.8% 27.59 [23.24; 32.29]
Azarkeivan, 2011 170 695 2.1% 0.8% 24.46 [21.31; 27.83]
Azarkeivan, 2012 30 395 1.2% 0.8% 7.59 [5.18; 10.66]
Barak, 2003 2 40 0.1% 0.7% 5.00 [0.61; 16.92]
Basiratnia, 2010 11 50 0.2% 0.8% 22.00 [11.53; 35.96]
Basiratnia, 2010 15 63 0.2% 0.8% 23.81 [13.98; 36.21]
Boroujerdnia, 2009 27 97 0.3% 0.8% 27.84 [19.21; 37.86]
Boroujerdnia, 2009 31 109 0.3% 0.8% 28.44 [20.21; 37.88]
Bozorghi, 2006 1 44 0.1% 0.8% 2.27 [0.06; 12.02]
Bozorghi, 2006 4 45 0.1% 0.8% 8.89 [2.48; 21.22]
Bozorgi, 2008 21 99 0.3% 0.8% 21.21 [13.64; 30.58]
Bozorgi, 2008 31 104 0.3% 0.8% 29.81 [21.23; 39.57]
Broumand, 2002 105 548 1.7% 0.8% 19.16 [15.95; 22.71]
Company, 2007 0 35 0.1% 0.7% 0.00 [0.00; 10.00]
Company, 2007 4 57 0.2% 0.8% 7.02 [1.95; 17.00]
Company, 2007 13 38 0.1% 0.7% 34.21 [19.63; 51.35]
Company, 2007 23 65 0.2% 0.8% 35.38 [23.92; 48.23]
Dadgaran, 2005 70 393 1.2% 0.8% 17.81 [14.16; 21.96]
Dadmanesh, 2015 0 138 0.4% 0.8% 0.00 [0.00; 2.64]
Eghbalian, 2000 18 53 0.2% 0.8% 33.96 [21.52; 48.27]
Esfahani, 2014 44 89 0.3% 0.8% 49.44 [38.67; 60.25]
Eslamifar, 2007 5 77 0.2% 0.8% 6.49 [2.14; 14.51]
Faranoush, 2006 222 630 1.9% 0.8% 35.24 [31.51; 39.11]
Ghaderi, 1996 62 90 0.3% 0.8% 68.89 [58.26; 78.23]
Ghadir, 2009 (1053) 19 90 0.3% 0.8% 21.11 [13.21; 30.99]
Ghafoorian-Broujerdnia, 2006 32 122 0.4% 0.8% 26.23 [18.68; 34.96]
Ghane, 2012 46 245 0.8% 0.8% 18.78 [14.09; 24.24]
Haghazali, 2011 6 76 0.2% 0.8% 7.89 [2.95; 16.40]
Hamissi, 2011 7 120 0.4% 0.8% 5.83 [2.38; 11.65]
Hamissi, 2011 6 75 0.2% 0.8% 8.00 [2.99; 16.60]
Hariri, 2006 67 616 1.9% 0.8% 10.88 [8.53; 13.61]
Hariri, 2006 77 120 0.4% 0.8% 64.17 [54.90; 72.71]
Jahromi, 2007 2 34 0.1% 0.7% 5.88 [0.72; 19.68]
Joukar, 2011 20 228 0.7% 0.8% 8.77 [5.44; 13.22]
Joukar, 2011 41 286 0.9% 0.8% 14.34 [10.49; 18.94]
Kalantari, 2011 50 545 1.7% 0.8% 9.17 [6.89; 11.92]
Kalantari, 2011 495 615 1.9% 0.8% 80.49 [77.13; 83.55]
Kalantari, 2014 26 499 1.5% 0.8% 5.21 [3.43; 7.54]
Karimi, 2001 44 281 0.9% 0.8% 15.66 [11.61; 20.44]
Karimi, 2001 73 466 1.4% 0.8% 15.67 [12.48; 19.29]
Karimi, 2002 48 367 1.1% 0.8% 13.08 [9.80; 16.96]
Kashef, 2008 24 131 0.4% 0.8% 18.32 [12.11; 26.02]
Kassaian, 2011 17 800 2.5% 0.8% 2.12 [1.24; 3.38]
Kassaian, 2011 60 570 1.8% 0.8% 10.53 [8.13; 13.34]
Keshvari, 2014 103 257 0.8% 0.8% 40.08 [34.04; 46.35]
Kiakalayeh, 2013 117 1113 3.4% 0.8% 10.51 [8.77; 12.46]
Lak, 2000 90 100 0.3% 0.8% 90.00 [82.38; 95.10]
Lak, 2000 212 385 1.2% 0.8% 55.06 [49.94; 60.11]
Langarodi, 2011 11 90 0.3% 0.8% 12.22 [6.26; 20.82]
Langarodi, 2011 18 116 0.4% 0.8% 15.52 [9.46; 23.41]
Mahdaviani, 2008 7 97 0.3% 0.8% 7.22 [2.95; 14.30]
Mahdaviani, 2008 25 68 0.2% 0.8% 36.76 [25.39; 49.33]
Mahdavimazdeh, 2009 227 2403 7.4% 0.8% 9.45 [8.31; 10.69]
Mak, 2001 27 86 0.3% 0.8% 31.40 [21.81; 42.30]
Makhlough, 2008 39 186 0.6% 0.8% 20.97 [15.36; 27.53]
Mansour-Ghanaei, 2002 72 101 0.3% 0.8% 71.29 [61.43; 79.85]
Mansour-Ghanaei, 2009 17 163 0.5% 0.8% 10.43 [6.19; 16.17]
Mansour-Ghanaei, 2009 186 370 1.1% 0.8% 50.27 [45.06; 55.48]
Mashayekhi, 2011 3 100 0.3% 0.8% 3.00 [0.62; 8.52]
Mirmomen, 2006 141 732 2.2% 0.8% 19.26 [16.47; 22.31]
Mobini, 2010 38 77 0.2% 0.8% 49.35 [37.76; 61.00]
Mousavi, 2002 22 81 0.3% 0.8% 27.16 [17.87; 38.19]
Mousavi, 2014 2 47 0.1% 0.8% 4.26 [0.52; 14.54]
Mousavian, 2011 802 1095 3.4% 0.8% 73.24 [70.51; 75.84]
Najafi, 2001 8 49 0.2% 0.8% 16.33 [7.32; 29.66]
Najafi, 2001 10 51 0.2% 0.8% 19.61 [9.82; 33.12]
Nani, 2007 125 553 1.7% 0.8% 22.60 [19.18; 26.32]
Rostami, 2013 15 1704 5.2% 0.8% 0.88 [0.49; 1.45]
Rostami, 2013 38 2259 6.9% 0.8% 1.68 [1.19; 2.30]
Sabour, 2003 37 140 0.4% 0.8% 26.43 [19.34; 34.54]
Salehi, 2014 4 40 0.1% 0.7% 10.00 [2.79; 23.66]
Samak, 2012 19 142 0.4% 0.8% 13.38 [8.25; 20.10]
Samarbaf-Zadeh, 2015 39 430 1.3% 0.8% 9.07 [6.53; 12.19]
Samimi-Rad, 2007 2 50 0.2% 0.8% 4.00 [0.49; 13.71]
Samimi-Rad, 2007 3 48 0.1% 0.8% 6.25 [1.31; 17.20]
Samimi-Rad, 2007 33 76 0.2% 0.8% 43.42 [32.08; 55.29]
Samimi-Rad, 2008 1 101 0.3% 0.8% 0.99 [0.03; 5.39]
Samimi-Rad, 2008 10 103 0.3% 0.8% 9.71 [4.75; 17.13]
Sanei, 2004 49 364 1.1% 0.8% 13.46 [10.13; 17.40]
Sarkari, 2012 3 49 0.2% 0.8% 6.12 [1.28; 16.87]
Seyrafian, 2006 16 556 1.7% 0.8% 2.88 [1.65; 4.63]
Shahshahani, 2006 8 85 0.3% 0.8% 9.41 [4.15; 17.71]
Shahshahani, 2006 36 74 0.2% 0.8% 48.65 [36.85; 60.56]
Sharifi-Mood, 2006 23 74 0.2% 0.8% 31.08 [20.83; 42.90]
Sharifi-Mood, 2007 24 81 0.3% 0.8% 29.63 [19.99; 40.81]
Somi, 2007 69 462 1.4% 0.8% 14.94 [11.81; 18.52]
Somi, 2014 37 455 1.4% 0.8% 8.13 [5.79; 11.03]
Taremi, 2005 66 324 1.0% 0.8% 20.37 [16.12; 25.17]
Taziki, 2008 61 497 1.5% 0.8% 12.27 [9.52; 15.49]
Taziki, 2008 64 348 1.1% 0.8% 18.39 [14.46; 22.87]
Toosi, 2007 11 130 0.4% 0.8% 8.46 [4.30; 14.64]
Torabi, 2005 6 84 0.3% 0.8% 7.14 [2.67; 14.90]
Torabi, 2006 72 130 0.4% 0.8% 55.38 [46.42; 64.10]
Vahidi, 2000 24 107 0.3% 0.8% 22.43 [14.93; 31.51]
Valizadeh, 2013 3 50 0.2% 0.8% 6.00 [1.25; 16.55]
Valizadeh, 2015 0 32 0.1% 0.7% 0.00 [0.00; 10.89]
Yazdani, 2012 232 350 1.1% 0.8% 66.29 [61.07; 71.22]
Zahedi, 2004 43 97 0.3% 0.8% 44.33 [34.24; 54.77]
Zahedi, 2012 7 228 0.7% 0.8% 3.07 [1.24; 6.22]
Ziaee, 2005 44 80 0.2% 0.8% 55.00 [43.47; 66.15]
Ziaee, 2007 21 80 0.2% 0.8% 26.25 [17.04; 37.29]
Ziaee, 2013 0 41 0.1% 0.7% 0.00 [0.00; 8.60]
Ziaee, 2015 22 108 0.3% 0.8% 20.37 [13.23; 29.20]
Fixed effect model 6453 32517 100% -- 16.34 [15.94; 16.75]
Random effects model -- 100% 19.98 [16.36; 23.85]
Prediction interval [0.00; 69.69]
Heterogeneity: I-squared=98.6%, tau-squared=0.2722, Q=8786.2, df=126, p<0.0001
0 20 40 60 80 100
HCV prevalence (95%CI)

B)

Author, Year	HCV positive	Sample size	W(Fixed)	W(Random)	Prev(%)	95% CI
Alavi, 2007	77	104	0.6%	1.8%	74.04	[64.52; 82.14]
Alavi, 2009	74	142	0.8%	1.8%	52.11	[43.58; 60.56]
Alipour, 2013	15	42	0.2%	1.6%	35.71	[21.55; 51.97]
Alipour, 2013	86	226	1.3%	1.8%	38.05	[31.70; 44.73]
Alizadeh, 2005	47	149	0.8%	1.8%	31.54	[24.18; 39.65]
Amini, 2005	22	34	0.2%	1.6%	64.71	[46.49; 80.25]
Amiri, 2007	72	81	0.5%	1.7%	88.89	[79.95; 94.79]
Asl, 2013	104	150	0.8%	1.8%	69.33	[61.29; 76.59]
Ataei, 2010	1248	3284	18.2%	1.9%	38.00	[36.34; 39.69]
Ataei, 2011	644	1485	8.2%	1.9%	43.37	[40.83; 45.93]
Ataei, 2011	27	136	0.8%	1.8%	19.85	[13.51; 27.55]
Davoodian, 2009	163	249	1.4%	1.8%	65.46	[59.20; 71.35]
Eskandarieh, 2013	168	258	1.4%	1.8%	65.12	[58.96; 70.92]
Honarvar, 2013	94	233	1.3%	1.8%	40.34	[33.99; 46.95]
Hosseini, 2010	334	417	2.3%	1.9%	80.10	[75.94; 83.82]
Imani, 2008	15	133	0.7%	1.8%	11.28	[6.45; 17.92]
Ismail, 2005	11	65	0.4%	1.7%	16.92	[8.76; 28.27]
Kaffashian, 2011	398	951	5.3%	1.9%	41.85	[38.69; 45.06]
Kassaian, 2012	392	943	5.2%	1.9%	41.57	[38.40; 44.79]
Keramat, 2011	126	199	1.1%	1.8%	63.32	[56.21; 70.02]
Khani, 2003	176	346	1.9%	1.9%	50.87	[45.47; 56.25]
Kheirandish, 2009	41	61	0.3%	1.7%	67.21	[54.00; 78.69]
Kheirandish, 2009	185	229	1.3%	1.8%	80.79	[75.08; 85.68]
Kheirandish, 2009	85	103	0.6%	1.8%	82.52	[73.79; 89.30]
Kheirandish, 2009	43	49	0.3%	1.7%	87.76	[75.23; 95.37]
Khorvash, 2008	39	53	0.3%	1.7%	73.58	[59.67; 84.74]
Mehrjerdi, 2014	56	209	1.2%	1.8%	26.79	[20.92; 33.34]
Meidani, 2009	39	150	0.8%	1.8%	26.00	[19.19; 33.79]
Mirahmadizadeh, 2004	149	186	1.0%	1.8%	80.11	[73.64; 85.59]
Mirahmadizadeh, 2009	406	936	5.2%	1.9%	43.38	[40.17; 46.62]
Mir-Nasseri, 2005	21	42	0.2%	1.6%	50.00	[34.19; 65.81]
Mir-Nasseri, 2005	287	425	2.4%	1.9%	67.53	[62.85; 71.96]
Mir-Nasseri, 2008	21	54	0.3%	1.7%	38.89	[25.92; 53.12]
Mir-Nasseri, 2008	287	464	2.6%	1.9%	61.85	[57.26; 66.29]
Mir-Nasseri, 2011	359	518	2.9%	1.9%	69.31	[65.13; 73.25]
Momen-Heravi, 2012	142	300	1.7%	1.9%	47.33	[41.57; 53.15]
Pourahmad, 2007	308	401	2.2%	1.9%	76.81	[72.36; 80.85]
Rahbar, 2004	60	101	0.6%	1.8%	59.41	[49.18; 69.07]
Rahimi-Movaghar, 2010	293	859	4.8%	1.9%	34.11	[30.94; 37.39]
Rahimi-Movaghar, 2010	16	36	0.2%	1.6%	44.44	[27.94; 61.90]
Ramzani, 2014	56	100	0.6%	1.8%	56.00	[45.72; 65.92]
Rostami-Jalilian, 2006	26	76	0.4%	1.7%	34.21	[23.71; 45.99]
Rostami-Jalilian, 2006	33	72	0.4%	1.7%	45.83	[34.02; 58.00]
Saleh, 2011	57	94	0.5%	1.8%	60.64	[50.02; 70.56]
Salehi, 2015	179	1327	7.4%	1.9%	13.49	[11.70; 15.45]
Sani, 2012	44	62	0.3%	1.7%	70.97	[58.05; 81.80]
Sarkari, 2012	67	158	0.9%	1.8%	42.41	[34.59; 50.51]
Sharif, 2009	24	200	1.1%	1.8%	12.00	[7.84; 17.33]
Sharifi-Mood, 2006	7	31	0.2%	1.6%	22.58	[9.59; 41.10]
Sofian, 2012	91	153	0.9%	1.8%	59.48	[51.25; 67.33]
Soudbakhsh, 2008	23	26	0.1%	1.5%	88.46	[69.85; 97.55]
Tayeri, 2008	80	106	0.6%	1.8%	75.47	[66.16; 83.31]
Zadeh, 2007	25	70	0.4%	1.7%	35.71	[24.61; 48.07]
Zali, 2001	182	402	2.2%	1.9%	45.27	[40.33; 50.28]
Zamani, 2007	105	202	1.1%	1.8%	51.98	[44.86; 59.04]
Zamani, 2010	71	117	0.7%	1.8%	60.68	[51.23; 69.59]
**Fixed effect model**	**8200**	**17999**	**100%**	**--**	**45.26**	**[44.53; 46.00]**
**Random effects model**			**--**	**100%**	**52.20**	**[46.91; 57.47]**
**Prediction interval**						**[15.82; 87.29]**

Heterogeneity: I-squared=97.9%, tau-squared=0.1515, Q=2615, df=55, p<0.0001

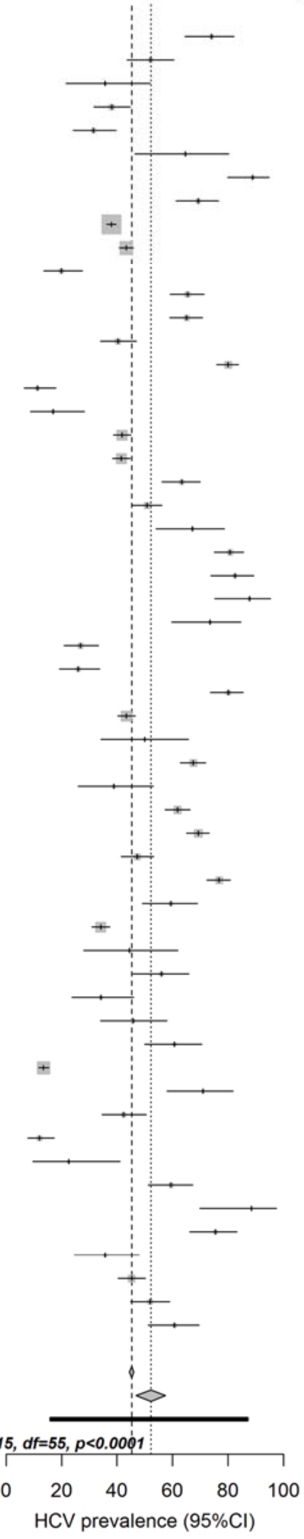

C)

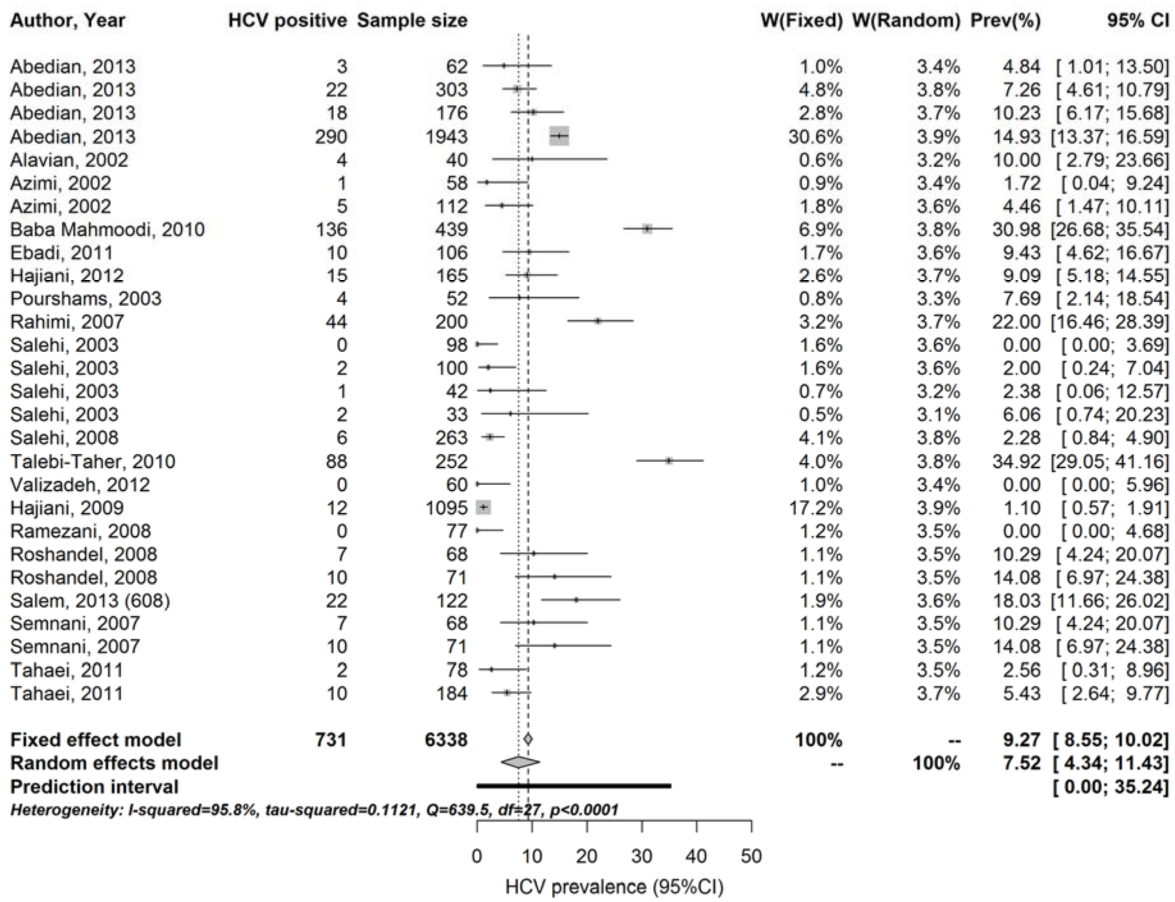
Author, Year HCV positive Sample size W(Fixed) W(Random) Prev(%) 95% CI
Abedian, 2013 3 62 1.0% 3.4% 4.84 [1.01; 13.50]
Abedian, 2013 22 303 4.8% 3.8% 7.26 [4.61; 10.79]
Abedian, 2013 18 176 2.8% 3.7% 10.23 [6.17; 15.68]
Abedian, 2013 290 1943 30.6% 3.9% 14.93 [13.37; 16.59]
Alavian, 2002 4 40 0.6% 3.2% 10.00 [2.79; 23.66]
Azimi, 2002 1 58 0.9% 3.4% 1.72 [0.04; 9.24]
Azimi, 2002 5 112 1.8% 3.6% 4.46 [1.47; 10.11]
Baba Mahmoodi, 2010 136 439 6.9% 3.8% 30.98 [26.68; 35.54]
Ebadi, 2011 10 106 1.7% 3.6% 9.43 [4.62; 16.67]
Hajiani, 2012 15 165 2.6% 3.7% 9.09 [5.18; 14.55]
Pourshams, 2003 4 52 0.8% 3.3% 7.69 [2.14; 18.54]
Rahimi, 2007 44 200 3.2% 3.7% 22.00 [16.46; 28.39]
Salehi, 2003 0 98 1.6% 3.6% 0.00 [0.00; 3.69]
Salehi, 2003 2 100 1.6% 3.6% 2.00 [0.24; 7.04]
Salehi, 2003 1 42 0.7% 3.2% 2.38 [0.06; 12.57]
Salehi, 2003 2 33 0.5% 3.1% 6.06 [0.74; 20.23]
Salehi, 2008 6 263 4.1% 3.8% 2.28 [0.84; 4.90]
Talebi-Taher, 2010 88 252 4.0% 3.8% 34.92 [29.05; 41.16]
Valizadeh, 2012 0 60 1.0% 3.4% 0.00 [0.00; 5.96]
Hajiani, 2009 12 1095 17.2% 3.9% 1.10 [0.57; 1.91]
Ramezani, 2008 0 77 1.2% 3.5% 0.00 [0.00; 4.68]
Roshandel, 2008 7 68 1.1% 3.5% 10.29 [4.24; 20.07]
Roshandel, 2008 10 71 1.1% 3.5% 14.08 [6.97; 24.38]
Salem, 2013 (608) 22 122 1.9% 3.6% 18.03 [11.66; 26.02]
Semnani, 2007 7 68 1.1% 3.5% 10.29 [4.24; 20.07]
Semnani, 2007 10 71 1.1% 3.5% 14.08 [6.97; 24.38]
Tahaei, 2011 2 78 1.2% 3.5% 2.56 [0.31; 8.96]
Tahaei, 2011 10 184 2.9% 3.7% 5.43 [2.64; 9.77]
Fixed effect model 731 6338 100% -- 9.27 [8.55; 10.02]
Random effects model -- 100% 7.52 [4.34; 11.43]
Prediction interval [0.00; 35.24]
Heterogeneity: I-squared=95.8%, tau-squared=0.1121, Q=639.5, df=27, p<0.0001
0 10 20 30 40 50
HCV prevalence (95%CI)

D)

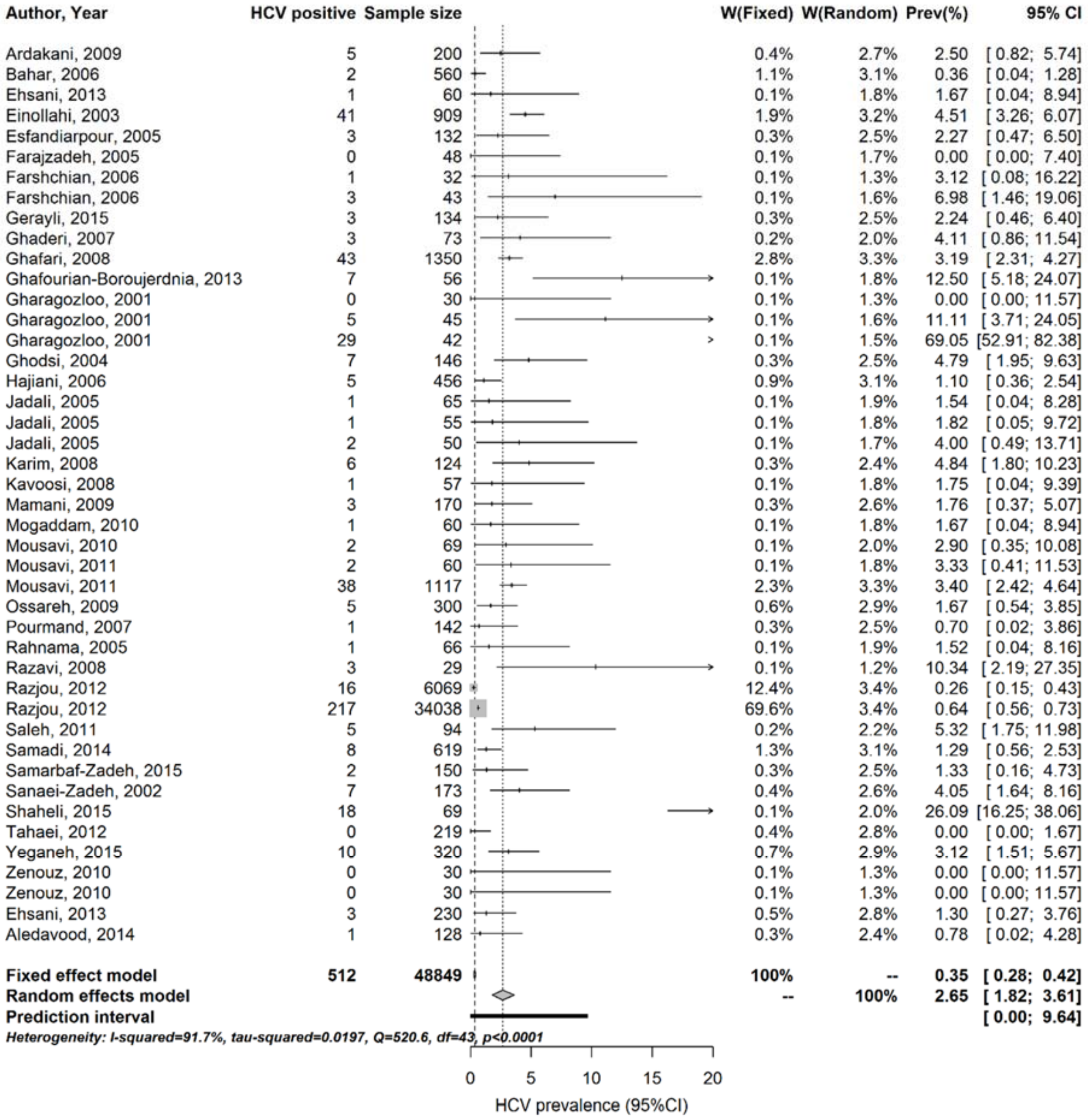
Author, Year HCV positive Sample size W(Fixed) W(Random) Prev(%) 95% CI
Ardakani, 2009 5 200 0.4% 2.7% 2.50 [0.82; 5.74]
Bahar, 2006 2 560 1.1% 3.1% 0.36 [0.04; 1.28]
Ehsani, 2013 1 60 0.1% 1.8% 1.67 [0.04; 8.94]
Einollahi, 2003 41 909 1.9% 3.2% 4.51 [3.26; 6.07]
Esfandiarpour, 2005 3 132 0.3% 2.5% 2.27 [0.47; 6.50]
Farajzadeh, 2005 0 48 0.1% 1.7% 0.00 [0.00; 7.40]
Farshchian, 2006 1 32 0.1% 1.3% 3.12 [0.08; 16.22]
Farshchian, 2006 3 43 0.1% 1.6% 6.98 [1.46; 19.06]
Gerayli, 2015 3 134 0.3% 2.5% 2.24 [0.46; 6.40]
Ghaderi, 2007 3 73 0.2% 2.0% 4.11 [0.86; 11.54]
Ghafari, 2008 43 1350 2.8% 3.3% 3.19 [2.31; 4.27]
Ghafourian-Boroujerdnia, 2013 7 56 0.1% 1.8% 12.50 [5.18; 24.07]
Gharagozloo, 2001 0 30 0.1% 1.3% 0.00 [0.00; 11.57]
Gharagozloo, 2001 5 45 0.1% 1.6% 11.11 [3.71; 24.05]
Gharagozloo, 2001 29 42 0.1% 1.5% 69.05 [52.91; 82.38]
Ghodsi, 2004 7 146 0.3% 2.5% 4.79 [1.95; 9.63]
Hajiani, 2006 5 456 0.9% 3.1% 1.10 [0.36; 2.54]
Jadali, 2005 1 65 0.1% 1.9% 1.54 [0.04; 8.28]
Jadali, 2005 1 55 0.1% 1.8% 1.82 [0.05; 9.72]
Jadali, 2005 2 50 0.1% 1.7% 4.00 [0.49; 13.71]
Karim, 2008 6 124 0.3% 2.4% 4.84 [1.80; 10.23]
Kavoosi, 2008 1 57 0.1% 1.8% 1.75 [0.04; 9.39]
Mamani, 2009 3 170 0.3% 2.6% 1.76 [0.37; 5.07]
Mogaddam, 2010 1 60 0.1% 1.8% 1.67 [0.04; 8.94]
Mousavi, 2010 2 69 0.1% 2.0% 2.90 [0.35; 10.08]
Mousavi, 2011 2 60 0.1% 1.8% 3.33 [0.41; 11.53]
Mousavi, 2011 38 1117 2.3% 3.3% 3.40 [2.42; 4.64]
Ossareh, 2009 5 300 0.6% 2.9% 1.67 [0.54; 3.85]
Pourmand, 2007 1 142 0.3% 2.5% 0.70 [0.02; 3.86]
Rahnama, 2005 1 66 0.1% 1.9% 1.52 [0.04; 8.16]
Razavi, 2008 3 29 0.1% 1.2% 10.34 [2.19; 27.35]
Razjou, 2012 16 6069 12.4% 3.4% 0.26 [0.15; 0.43]
Razjou, 2012 217 34038 69.6% 3.4% 0.64 [0.56; 0.73]
Saleh, 2011 5 94 0.2% 2.2% 5.32 [1.75; 11.98]
Samadi, 2014 8 619 1.3% 3.1% 1.29 [0.56; 2.53]
Samarbaf-Zadeh, 2015 2 150 0.3% 2.5% 1.33 [0.16; 4.73]
Sanaei-Zadeh, 2002 7 173 0.4% 2.6% 4.05 [1.64; 8.16]
Shaheli, 2015 18 69 0.1% 2.0% 26.09 [16.25; 38.06]
Tahaei, 2012 0 219 0.4% 2.8% 0.00 [0.00; 1.67]
Yeganeh, 2015 10 320 0.7% 2.9% 3.12 [1.51; 5.67]
Zenouz, 2010 0 30 0.1% 1.3% 0.00 [0.00; 11.57]
Zenouz, 2010 0 30 0.1% 1.3% 0.00 [0.00; 11.57]
Ehsani, 2013 3 230 0.5% 2.8% 1.30 [0.27; 3.76]
Aledavood, 2014 1 128 0.3% 2.4% 0.78 [0.02; 4.28]
Fixed effect model 512 48849 100% -- 0.35 [0.28; 0.42]
Random effects model -- 100% 2.65 [1.82; 3.61]
Prediction interval [0.00; 9.64]
Heterogeneity: I-squared=91.7%, tau-squared=0.0197, Q=520.6, df=43, p<0.0001
0 5 10 15 20
HCV prevalence (95%CI)

Fig S5. Forest plot presenting results of the sensitivity analysis of excluding blood donor data in the general population meta-analysis.

Author, Year	HCV positive	Sample size	W(Fixed)	W(Random)	Prev(%)	95% CI
Alavi, 2012	0	90	0.1%	0.7%	0.00	[0.00; 4.02]
Alavian, 2002	3	307	0.3%	1.6%	0.98	[0.20; 2.83]
Alavian, 2015	0	275	0.3%	1.5%	0.00	[0.00; 1.33]
Ansari-Moghaddam, 2012	5	1380	1.4%	3.1%	0.36	[0.12; 0.84]
Ansari-Moghaddam, 2012	8	1207	1.2%	3.0%	0.66	[0.29; 1.30]
Ardebili, 2012	15	6095	6.0%	3.9%	0.25	[0.14; 0.41]
Babak, 2008	15	1721	1.7%	3.3%	0.87	[0.49; 1.43]
Chamani, 2007	0	716	0.7%	2.5%	0.00	[0.00; 0.51]
Chamani, 2007	2	533	0.5%	2.2%	0.38	[0.05; 1.35]
Esfandiarpour, 2005	2	149	0.1%	1.0%	1.34	[0.16; 4.76]
Esmaeili, 2004	0	100	0.1%	0.7%	0.00	[0.00; 3.62]
Esmaeili, 2004	2	100	0.1%	0.7%	2.00	[0.24; 7.04]
Gerayli, 2015	0	134	0.1%	0.9%	0.00	[0.00; 2.72]
Ghadir, 2006	4	736	0.7%	2.5%	0.54	[0.15; 1.39]
Ghadir, 2006	18	1387	1.4%	3.1%	1.30	[0.77; 2.04]
Ghezeldasht, 2015	7	1227	1.2%	3.0%	0.57	[0.23; 1.17]
Hajiani, 2006	6	500	0.5%	2.1%	1.20	[0.44; 2.59]
Heydarabad, 2012	2	420	0.4%	1.9%	0.48	[0.06; 1.71]
Jadali, 2005	0	50	0.0%	0.4%	0.00	[0.00; 7.11]
Jadali, 2005	0	44	0.0%	0.4%	0.00	[0.00; 8.04]
Jadali, 2005	0	50	0.0%	0.4%	0.00	[0.00; 7.11]
Jamali, 2008	12	1351	1.3%	3.1%	0.89	[0.46; 1.55]
Jamali, 2008	8	698	0.7%	2.5%	1.15	[0.50; 2.25]
Kavoosi, 2008	1	57	0.1%	0.4%	1.75	[0.04; 9.39]
Kordi, 2011	0	410	0.4%	1.9%	0.00	[0.00; 0.90]
Kordi, 2011	2	420	0.4%	1.9%	0.48	[0.06; 1.71]
Merat, 2010	50	5684	5.6%	3.9%	0.88	[0.65; 1.16]
Moezzi, 2015	40	3000	3.0%	3.6%	1.33	[0.95; 1.81]
Mohebbi, 2011	0	174	0.2%	1.1%	0.00	[0.00; 2.10]
Mohebbi, 2011	1	526	0.5%	2.2%	0.19	[0.00; 1.05]
Mohebbi, 2011	1	127	0.1%	0.9%	0.79	[0.02; 4.31]
Monsour-Ghanaei, 2007	9	383	0.4%	1.9%	2.35	[1.08; 4.41]
Moradi, 2007	5	356	0.4%	1.8%	1.40	[0.46; 3.25]
Motlagh, 2001	0	80	0.1%	0.6%	0.00	[0.00; 4.51]
Mouasvi, 2011	0	52	0.1%	0.4%	0.00	[0.00; 6.85]
Mouasvi, 2013	0	30	0.0%	0.2%	0.00	[0.00; 11.57]
Mousavi, 2010	0	79	0.1%	0.6%	0.00	[0.00; 4.56]
Poustchi, 2011	247	49338	48.5%	4.1%	0.50	[0.44; 0.57]
Roshan, 2012	3	712	0.7%	2.5%	0.42	[0.09; 1.23]
Roshan, 2012	6	712	0.7%	2.5%	0.84	[0.31; 1.83]
Shaheli, 2015	0	100	0.1%	0.7%	0.00	[0.00; 3.62]
Shakeri, 2013	5	3870	3.8%	3.7%	0.13	[0.04; 0.30]
Shamsdin, 2012	15	2080	2.0%	3.4%	0.72	[0.40; 1.19]
Sohrabpour, 2010	49	5589	5.5%	3.8%	0.88	[0.65; 1.16]
Tanomand, 2007	5	346	0.3%	1.7%	1.45	[0.47; 3.34]
Vahidi, 2000	0	107	0.1%	0.8%	0.00	[0.00; 3.39]
Yazdani, 2006	12	2000	2.0%	3.4%	0.60	[0.31; 1.05]
Zamani, 2013	0	2662	2.6%	3.5%	0.00	[0.00; 0.14]
Zamani, 2013	5	3483	3.4%	3.7%	0.14	[0.05; 0.33]
Zenouz, 2010	0	30	0.0%	0.2%	0.00	[0.00; 11.57]
**Fixed effect model**	**565**	**101677**	**100%**	**--**	**0.26**	**[0.22; 0.30]**
**Random effects model**			**--**	**100%**	**0.33**	**[0.21; 0.47]**
**Prediction interval**						**[0.00; 1.25]**

Heterogeneity: I-squared=76.3%, tau-squared=0.0021, Q=206.9, df=49, p<0.0001

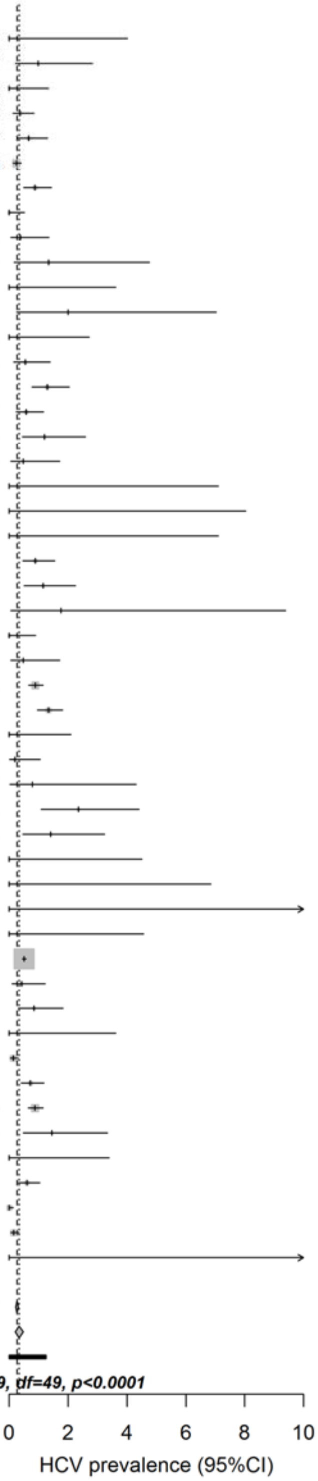

References

1 Moher, D., Liberati, A., Tetzlaff, J. & Altman, D. G. Preferred reporting items for systematic reviews and meta-analyses: the PRISMA statement. *Annals of internal medicine* **151**, 264-269 (2009).

2 Ahmad Akhoundi, M. S. *et al.* Prevalence of blood-borne viruses among Iranian dentists: Results of a national survey. *Int J Occup Med Environ Health* **28**, 593-602 (2015).

3 Alavi-Naini, R. *et al.* Risk factors of hepatitis C infection among household contacts in Zahedan. *MilMed Journal* **7**, 343-348 (2006).

4 Alipour, A., Haghdoost, A. A., Sajadi, L. & Zolala, F. HIV prevalence and related risk behaviours among female partners of male injecting drugs users in Iran: Results of a bio-behavioural survey, 2010. *Sexually Transmitted Infections* **89**, iii41-iii44 (2013).

5 Alipour, A., Rezaianzadeh, A., Hasanzadeh, J., Rajaeefard, A. & Davarpanah, M. A. Sexual transmission of hepatitis C virus between HIV infected subjects and their main heterosexual partners. *Hepatitis Monthly* **13** (2013).

6 Alizadeh, A. H. M., Alavian, S. M., Jafari, K. & Yazdi, N. Prevalence of hepatitis C virus infection and its related risk factors in drug abuser prisoners in Hamedan - Iran. *World Journal of Gastroenterology* **11**, 4085-4089 (2005).

7 Mohtasham Amiri, Z., Rezvani, M., Jafari Shakib, R. & Jafari Shakib, A. Prevalence of hepatitis C virus infection and risk factors of drug using prisoners in Guilan province. *Eastern Mediterranean Health Journal* **13**, 250-256 (2007).

8 Amiri, F. B. *et al.* Vulnerability of homeless people in Tehran, Iran, to HIV, tuberculosis and viral hepatitis. *PLoS ONE* **9** (2014).

9 Asgari, F., Gouya, M., Mohammad, K., Fotouhi, A. & Yousefi, A. Hepatitis C virus infection among Iranian prisoners and its relation with addiction, 2001-2005. *Mirror of Heritage* **11**, 1-8 (2008).

10 Saleh, A., Sharifi, M., Nooruznejad, S. & Vezirian, S. HIV/AIDS, hepatitis B and C infection status in the staff of laboratories of Kermanshah medical centers (2002) [Persian]. *Journal of Kermanshah University of Medical Sciences* **7**, 49-54 (2003).

11 Hosseini Asl, S. K., Avijgan, M. & Mohamadnejad, M. High prevalence of HBV, HCV, and HIV infections in gypsy population residing Shahr-e-Kord. *Archives of Iranian Medicine* **7**, 20-22 (2004).

12 Ataei, B. *et al.* The prevalence of hepatitis C and HIV infection in street children and adolescent in Isfahan. *Tehran University Medical Journal* **67**, 811-816 (1999).

13 Ataei, B. *et al.* Seroprevalence of hepatitis C (HCV) infection among street children in Isfahan, Iran. *Hepatology International* **5 (1)**, 227 (2011).

14 Z. Azarkar, Gh. Sharifzadeh & MA. Miraki. HBV, HCV and HIV prevalence among - south Khorasan prisoners. *Journal of Birjand University of Medical Sciences* **14**, 9-15 (2007).

15 Azarkar, Z. & Sharifzadeh, G. Evaluation of the prevalence of Hepatitis B, Hepatitis C, and HIV in inmates with drug-related convictions in Birjand, Iran in 2008. *Hepatitis Monthly* **10**, 26-30 (2010).

16 Azizi, A., Amirian, F. & Amirian, M. Prevalence and Associated Factors of Hepatitis C in Self-introduced Substance Abusers. *Hayat* **17**, 55-61 (2011).

17 Bassirat-nia, M. Hepatitis C among families of thalassemic patients suffering from hepatitis C in Shahrekord, 1999. *Journal of Shahrekord University of Medical Sciences* **3**, 33-36 (2001).

18 Behnaz, M., Behnaz, F. & Mohammadzadeh, M. The prevalence of hepatitis C infection among the dentists of Yazd and assessment of their knowledge about hepatitis. *Journal of Dental Medicine* **17**, 55-59 (2004).
19 Behnaz, K., Abdollah, A., Fateme, F. & Mohammadreza, R. Prevalence and risk factors of HIV, hepatitis B virus and hepatitis C virus infections in drug addicts among Gorgan prisoners. *Journal of Medical Sciences* **7**, 252-254 (2007).
20 Dolan, K. *et al.* Six-month follow-up of Iranian women in methadone treatment: drug use, social functioning, crime, and HIV and HCV seroincidence. *Subst Abuse Rehabil* **3**, 37-43 (2012).
21 Sarkari, B. *et al.* High prevalence of hepatitis C infection among high risk groups in Kohgiloyeh and Boyerahmad Province, Southwest Iran. *Archives of Iranian Medicine* **15**, 271-274 (2012).
22 Fallah F *et al.* The assessment of hepatitis B and C prevalence in street children of Tehran from Farvardin to Shahrivar 1386. *Pejouhesh* **32**, 147-151 (2008).
23 Hajiani, E., Masjedizadeh, R., Hashemi, J., Azmi, M. & Rajabi, T. Hepatis C virus transmission and its risk factors within families of patients infected with hepatitis C virus in southern Iran: Khuzestan. *World J Gastroenterol* **12**, 7025-7028 (2006).
24 E., H., Arasteh, E., Ghorbani, S. & Mahdvi, S. Prevalence of hepatitis B, hepatitis C and HIV infection in 15 year and older patients admitted in hand surgery emergency *Scientific Journal of Kurdistan University of Medical Sciences* **8**, 25-32 (2003).
25 Honarvar, B. *et al.* Blood-borne hepatitis in opiate users in Iran: A poor outlook and urgent need to change nationwide screening policy. *PLoS ONE* **8** (2013).
26 Imani, R. *et al.* Seroprevalence of hepatitis C virus in the families of the patients with hepatitis C infection in Shahre-Kord, Iran. *Iranian Red Crescent Medical Journal* **12**, 472-475 (2010).
27 Jahani, M. R., Alavian, S. M., Shirzad, H., Kabir, A. & Hajarizadeh, B. Distribution and risk factors of hepatitis B, hepatitis C, and HIV infection in a female population with "illegal social behaviour". *Sex Transm Infect* **81**, 185 (2005).
28 Kamangar, E., Atapour, M., Sanei-Moghadam, E., Zohour, A. & Nayeb-Aghaie, S. M. Prevalence of serologic markers of Hepatitis B and C and risk factors among dentists and physicians in Kerman, Iran. *Journal of Kerman University of Medical Sciences* **10**, 240-245 (2003).
29 Kazerani, H. Epidemiologic survey of positive HIV, HCV, HBV tests among patients admitted for cardiac surgery and invasive procedures, In Emam Ali Hospital, in Kermanshah. *Scientific Journal of Kurdistan University of Medical Sciences* **11**, 42-47 (2007).
30 Kassaian, N., Ataei, B., Yaran, M., Babak, A. & Shoaei, P. Hepatitis B and C among women with illegal social behavior in Isfahan, Iran: Seroprevalence and associated factors. *Hepatitis Monthly* **11**, 368-371 (2011).
31 Khodadizadeh, A., Esmaeili Nadimi, A., Hosseini, S. & Shabani, S. Surveying the prevalence of human immunodeficiency virus, hepatitis C and hepatitis B in drug addcits referred to self-referral clinic of Rafsanjan University of Medical Sciences in 2003. *Journal of Rafsanjan University of Medical Sciences* **5**, 23-30 (2006).
32 Amini Lari, M., Ameli, F., Afsar Kazerooni, P. & Joulaei, H. in *International Congress on HIV/AIDS Women and Children* (Shiraz, Iran 2010).

33 Metanat, M. *et al.* Prevalence of hepatitis C among diabetes mellitus patients in Zahedan. *Zahedan Journal of Research in Medical Sciences* **8**, 179-186 (2006).

34 Moayedi-Nia, S. *et al.* HIV, HCV, HBV, HSV, and syphilis prevalence among female sex workers in Tehran, Iran, by using respondent-driven sampling. *AIDS Care - Psychological and Socio-Medical Aspects of AIDS/HIV* **28**, 487-490 (2016).

35 Mohammadalizadeh, A., Alavian, S., Jafari, K. & Yazdi, N. Prevalence of HBs Ag, HCV Ab & HIV Ab In the addict prisoners of Hamadan prison (Iran, 1998). *J Res Med Sci* **7**, 311-313 (2003).

36 Mousavijahed, Z. & Rezvan, H. Surveying the relationship between occupational exposure and hepatitis C infection. *Pejouhandeh* **6**, 85-88 (2001).

37 Aghamohammadzadeh, N., Ghostleslu, R., Javadi, M., Farzad, N. & Niafar, M. Prevalence of hepatitis C in type II diabetic patients [Persian]. *Medical Journal of Tabriz University of Medical Sciences* **32**, 7-11 (2010).

38 Nokhodian, Z. *et al.* Seroprevalence and risk factors of hepatitis C virus among juveniles in correctional center in Isfahan, Iran. *International Journal of Preventive Medicine* **3** (2012).

39 Nokhodian, Z. *et al.* Prevalence and risk factors of HIV, syphilis, hepatitis B and C among female prisoners in Isfahan, Iran. *Hepatitis Monthly* **12**, 92-97 (2012).

40 Norouzian, H. *et al.* Prevalence of HCV Infections and Co-Infection With HBV and HIV and Associated Risk Factors Among Addicts in Drug Treatment Centers, Lorestan Province, Iran. *Int J High Risk Behav Addict* **5**, e25028 (2016).

41 Peyvandi, M., Shobiri, H. & Farzad, S. Prevalence of Positive Serology for Hepatitis C in an Orthopaedic Trauma Unit. *Iranian Journal of Orthpaedic Surgery* **5**, 7-10 (2006).

42 Pourahmad, M., Javady, A., Karimi, I., Ataei, B. & Kassaeian, N. Seroprevalence of and risk factors associated with hepatitis B, hepatitis C, and human immunodeficiency virus among prisoners in Iran. *Infectious Diseases in Clinical Practice* **15**, 368-372 (2007).

43 Rostami, M., Jorfi, M. & Alimohammadi, M. Prevalence of HBS, HCV and HIV in individual referred to laboratory of Imam Ali hospital of Andimeshk city in 2009. *Jentashapir Journal of Health Research* **3**, 297-302 (2011).

44 Saffar, M., Jooyan, A., Mahdavi, M. & Khalilian, A. Seroprevalence of hepatitis A, B, and C and hepatitis B vaccination status among health care workers in Sari-Iran, 2003. *J Mazand Univ Med Sci* **15**, 67-77 (2005).

45 Sharifi, M. *et al.* Prevalence of antibodies to hepatitis and AIDS viuses among dentists in Qazvin. *Iranian Journal of Medical Microbiology* **2**, 55-61 (2008).

46 Shoaei, P. *et al.* Seroprevalence of hepatitis C infection among laboratory health care workers in Isfahan, Iran. *International Journal of Preventive Medicine* **3** (2012).

47 Shoaei, P. *et al.* Seroprevalence of hepatitis B and C virus infection among barbers in Isfahan, Iran. *Hepatology International* **7**, S266-S267 (2013).

48 Tajbakhsh, E. & Paydar, F. Seroepidemiological study of hepatitis C virus in prisons in Shahrekord prison [Persian]. *World of Microbes* **1**, 23-27 (2008).

49 Talaie, H. *et al.* The prevalence of hepatitis B, hepatitis C and HIV infections in non-IV drug opioid poisoned patients in Tehran-Iran. *Pakistan Journal of Biological Sciences* **10**, 220-224 (2007).

50 Vahdani, P., Hosseini-Moghaddam, S. M., Gachkar, L. & Sharafi, K. Prevalence of hepatitis B, hepatitis C, human immunodeficiency virus, and syphilis among street

children residing in southern Tehran, Iran. *Archives of Iranian Medicine* **9**, 153-155 (2006).

51 Vahdani, P., Hosseini-Moghaddam, S. M., Family, A. & Moheb-Dezfouli, R. Prevalence of HBV, HCV, HIV, and syphilis among homeless subjects older than fifteen years in Tehran. *Archives of Iranian Medicine* **12**, 483-487 (2009).

52 Yarmohammadi, M. Investigating the serological status and epidemiological aspects of health care workers' exposure to HBV and HCV viruses. *Knowledge & Health* **5**, 37-42 (2011).

53 Esmaeili Nadimi, A. & Hosseini, S. The prevalence of HIV, HBV and HCV in narcotic addicted persons referred to the out patient clinic of rafsanjan university of medical sciences in 2003. *Journal of Rafsanjan University of Medical Sciences* **5**, 23-30 (2006).

54 Zakizad, M. *et al.* Seroprevalence of hepatitis C infection and associated risk factors among addicted prisoners in Sari-Iran. *Pakistan journal of biological sciences: PJBS* **12**, 1012-1018 (2009).

55 Ziaee, M., Sharifzadeh, G., Namaee, M. H. & Fereidouni, M. Prevalence of HIV and Hepatitis B, C, D Infections and Their Associated Risk Factors among Prisoners in Southern Khorasan Province, Iran. *Iranian Journal of Public Health* **43**, 229-234 (2014).

56 Abedian, S., Firoozi, M. & Malekzadeh, R. Etiology of liver cirrhosis in Iran: Single center experience in a large referral center, 2000-2011. *Journal of Gastroenterology and Hepatology* **28**, 615 (2013).

57 Abedian, S., Firoozi, M. & Malekzadeh, R. Etiology of hepatocellular carcinoma in IRAN: Single center experience in a large referral center, 2000-2011. *Journal of Gastroenterology and Hepatology* **28**, 607 (2013).

58 Aledavood, S. A. *et al.* Hepatitis-C infection incidence among the non-Hodgkin's B-cell lymphoma patients in the Northeast of Iran. *Iranian Journal of Cancer Prevention* **7**, 147-151 (2014).

59 Ardakani, M. J. E. *et al.* Frequency of hepatitis C in patients with rheumatoid arthritis. *Iranian Journal of Clinical Infectious Diseases* **4**, 39-43 (2009).

60 Azimi, K. *et al.* Causes of cirrhosis in a series of patients at a university hospital in Tehran. *Govaresh Journal* **7**, 19-26 (2002).

61 Baba Mahmoodi, F., Baba Mahmoodi, A., Valipour, R. & Delavarian, L. Prevalence of viral hepatitis and clinical epidemiology and prognosis of hepatitis A in adult patients admitted to Razi University Hospital Iran 2003-2008. *J Mazandaran Univ Med Sci* **20**, 2-9 (2010).

62 Bahar, A. & Azizi, F. Insulin Resistance and β Cell Function in Patients with Chronic Hepatitis and Impaired Glucose Tolerance. *Int J Endocrinol Metab* **4**, 125-133 (2007).

63 Ebadi, M. *et al.* Prevalence of HCV and HGV infections in iranian liver transplant recipients. *Transplantation Proceedings* **43**, 618-620 (2011).

64 Ehsani, A. H., Ghodsi, S. Z., Nourmohammad-Pour, P., Aghazadeh, N. & Damavandi, M. R. Pigmented purpura dermatosis and viral hepatitis: a case-control study. *Australas J Dermatol* **54**, 225-227 (2013).

65 Einollahi, B. *et al.* Pretransplant hepatitis C virus infection and its effect on the post-transplant course of living renal allograft recipients. *Journal of Gastroenterology and Hepatology* **18**, 836-840 (2003).

66 Esfandiarpour, I., Zandi, S., Rahnama, Z. & Dervish, D. Prevalence of anti-HCV-Ab (C) antibacterial antibody in psoriasis patients in Kerman [Persian]. *Scientific Journal of Hamadan University of Medical Sciences & Health services* **12**, 5-9 (2005).
67 Gerayli, S. *et al.* The association between oral lichen planus and hepatitis C virus infection; a report from northeast of Iran. *Jundishapur Journal of Microbiology* **8**, e16741 (2015).
68 Ghaderi , R. & Makhmalbaf , Z. The Relationship between Lichen Planus and Hepatitis C in Birjand, Iran. *Shiraz E-Med J* **8**, 72-79 (2007).
69 Ghafari, A. & Sanadgol, H. Impact of Hepatitis B and Hepatitis C Virus Infections on Patients and Allograft Outcomes in Renal Transplant Recipients: A Single Center Study. *Transplantation Proceedings* **40**, 196-198 (2008).
70 Ghafourian-Boroujerdnia, M., Assarehzadegan, M. A. & Zandian, K. Hepatitis B and C infections and different genotypes of HCV among sickle cell anemia patients in Ahvaz, South-Western Iran. *Jundishapur Journal of Microbiology* **6** (2013).
71 Gharagozloo, S., Khoshnoodi, J. & Shokri, F. Hepatitis C virus infection in patients with essential mixed cryoglobulinemia, multiple myeloma and chronic lymphocytic leukemia. *Pathol Oncol Res* **7**, 135-139 (2001).
72 Ghodsi, S. Z., Daneshpazhooh, M., Shahi, M. & Nikfarjam, A. Lichen planus and hepatitis C: A case-control study. *BMC Dermatology* **4** (2004).
73 Hajiani, e., Hashemi, s. j., Masjedi-zade, a. & Cheraghi, m. Risk of Hepatitis C Virus transmission Following Upper Gastrointestinal Endoscopy. *Yafteh* **8**, 47-54 (2006).
74 Hajiani, E., Hashemi, S. J. & Masjedizadeh, A. R. Seroepidemiology of hepatitis B virus infection in Khuzestan Province, Southwest of Iran. *Hepatitis Monthly* **9**, 34-38 (2009).
75 Hajiani, E., Hashemi, S., Masjedizadeh, R. & Ahmadzadeh, S. Liver Cirrhosis seen in GI Clinics of Ahvaz, Iran. *Govaresh* **17**, 178-182 (2012).
76 Jadali, Z. *et al.* Hepatitis C virus antibodies and vitiligo disease. *Iranian Journal of Public Health* **34**, 23-26 (2005).
77 Jadali, Z., Esfahanian, F., Farhoud, D., Alavian, S. & Soltan Dallal, M. Hashimoto's Thyroiditis and Its Association with Hepatitis C Virus Infection. *Int J Endocrinol Metab* **3**, 116-120 (2005).
78 Mowla, K. & Hajiani, E. Prevalence of Hepatitis C Virus Infection in Patients with Systemic Lupus Erythematosus: A Case-Control Study. *Hepat Mon* **8**, 41-44 (2008).
79 Kavoosi, H., Ebrahimi, A., Rezaei, M. & Jahani, M. Association of Lichen Planus with Hepatitis B and C. *Journal of Kermanshah University of Medical Sciences* **11** (2008).
80 Mamani, M., Hashemi, S. H., Niayesh, A., Ghaleiha, A. & Hajilooei, M. Study on the frequency of hepatitis B and C infection in chronic psychiatric patients in Hamedan in 2006-2007. *J Pak Med Assoc* **59**, 505-507 (2009).
81 Mogaddam, M. R. & Anamzade, F. Survey of relationship between hepatitis C and lichen planus among dermatology outpatients of Imam Hospital of Ardabil city. *Journal of Pakistan Association of Dermatologists* **20**, 19-22 (2010).
82 Beladi Mousavi, S. S., Hayati, F. & Ghorbani, A. Seroprevalence of cytomegalovirus antibody in renal transplant recipients and donors in Khuzestan Province, Iran. *Shiraz E Medical Journal* **11**, 203-208 (2010).
83 Beladi Mousavi, S. S. *et al.* Epidemiology of hepatitis C virus infection in ESRD patients in khuzestan province, Iran. *Iranian Journal of Kidney Diseases* **5**, 44 (2011).

84 Ossareh, S., Naseem, S., Faraji, M. A., Bahrami Asl, M. & Yousefnejad, A. Frequency and Risk Factors for Posttransplant Diabetes Mellitus in Iranian Renal Transplant Patients. *Transplantation Proceedings* **41**, 2814-2816 (2009).
85 Pourmand, G. *et al.* Infectious complications after kidney transplantation: A single-center experience. *Transplant Infectious Disease* **9**, 302-309 (2007).
86 Pourshams, A. Etiology of persistently elevated aminotransferases level in blood donors from Tehran center of blood transfusion. *Govaresh Journal* **8**, 90-94 (2003).
87 Jadali, Z., Esfahanian, F., Eslami, M. B. & Sanati, M. H. Serum Antibodies against Hepatitis C Virus in Iranian Patients with Graves' Disease. *Iranian journal of allergy, asthma, and immunology* **4**, 91-94 (2005).
88 Rahimi, M. *et al.* Liver enzymes in 200 patients with acute viral hepatitis A, B, C. *Quarterly Iranian Journal Infection Diseases and Tropical Medicine* **12**, 77-81 (2007).
89 Rahnama, Z., Esfandiarpour, I. & Farajzadeh, S. The relationship between lichen planus and hepatitis C in dermatology outpatients in Kerman, Iran. *International Journal of Dermatology* **44**, 746-748 (2005).
90 Ramezani, A. *et al.* Rate of YMDD motif mutants in lamivudine-untreated Iranian patients with chronic hepatitis B virus infection. *International Journal of Infectious Diseases* **12**, 252-255 (2008).
91 Razjou, F., Maghsudlu, M., Nasizadeh, S. & Zadsar, M. The impact of donor selection on blood safety in Iran. *Transfusion and Apheresis Science* **47**, 13-16 (2012).
92 Roshan, R. *et al.* Prevalence of hepatitis B infection with hepatitis C and hepatitis D in Golestan province [Persian]. *Journal of Medical Sciences* **9**, 61-65 (2007).
93 Saleh, M., Mohammad, K., Saleh, A., Asghar, H. & Rasool, S. Prevalence of HIV, hepatitis B and C seropositivity in expired IV drug abusers in Hamedan. *Prevalence of HIV, hepatitis B and C seropositivity in expired IV drug abusers in Hamedan,* **16**, 253-257 (2011).
94 Salehi, M., Sanei, M. & Khosravi, S. Etiology of acute viral Hepatitis in Zahedan. *Pajouhesh Dar Pezeshki* **26**, 245-248 (2003).
95 Salehi, M. *et al.* Acute viral hepatitis in south-eastern of Iran: A serological analysis of 263 cases. *Acta Medica Iranica* **46**, 417-422 (2008).
96 Salem, F. *et al.* Prevalence and risk factors of hepatitis B virus genotype d amongst inmates in alborz province, Iran: A cross-sectional survey. *Jundishapur Journal of Microbiology* **6** (2013).
97 Samarbaf-Zadeh, A. R. *et al.* Prevalence of hepatitis G virus among hemodialysis and kidney transplant patients in Khuzestan Province, Iran. *Jundishapur Journal of Microbiology* **8**, 1-5 (2015).
98 Samadi, M. *et al.* The comparison of the prevalence rates of HBV, HCV, and HIV in blood donors having deferred for high risk behaviors. *Scientific Journal of Iranian Blood Transfusion Organization* **10**, 347-352 (2014).
99 Sanaei-Zadeh, H., Amoei, M. & Taghaddosinejad, F. Seroprevalence of HIV, HBV and HCV in forensic autopsies, of presumed low risk, in Tehran, the capital of Iran. *Journal of clinical forensic medicine* **9**, 179-181 (2002).
100 Semnani, S. *et al.* Hepatitis B/C virus co-infection in Iran: A seroepidemiological study. *Turkish Journal of Gastroenterology* **18**, 20-21 (2007).

101 Shaheli, M., Yaghobi, R., Rezaeian, A., Saadi, M. I. & Ramzi, M. Study of the associations between TT Virus single and mixed infections with leukemia. *Jundishapur Journal of Microbiology* **8**, e18212 (2015).
102 Tahaei, S. M. E. *et al.* Frequency of HIV and HCV co-infections in chronic HBV patients referred to Taleghani Hospital, Tehran, Iran from 2006 to 2010. *Hepatitis Monthly* **11**, 993-996 (2011).
103 Tahaei, S. M. *et al.* Evaluation of antibody frequency against HBV, HCV and HTLV-1. *Gastroenterology and hepatology from bed to bench* **5**, 161-165 (2012).
104 Talebi-Taher, M., Mohit, M. & Avanessians, T. B. Viral hepatitis in patients hospitalized in two teaching hospitals, Tehran, Iran. *Iranian Journal of Clinical Infectious Diseases* **5**, 25-29 (2010).
105 Valizadeh, N., Mehdioghli, R. & Behroozian, R. Etiologic and epidemiologic study of hepatocellular carcinoma in West Azarbaijan of Iran (2006-2011). *Indian Journal of Medical and Paediatric Oncology* **33**, 221-223 (2012).
106 Yeganeh, A. *et al.* Prevalence of Hepatitis B virus, Hepatitis C virus and human immunodeficiency virus infections among patients candidate for orthopedic trauma surgeries. *Med J Islam Repub Iran* **29**, 274 (2015).
107 Abdollahi, A. *et al.* Seroprevalence of Human Immunodificiency Virus (HIV) and Hepatitis C Infection in Hemophilic Patients in Iran. *Iranian Journal of Pathology* **3**, 119-124 (2008).
108 Alvai, S., Arzanian, M., Hatami, K. & Shirani, A. Frequency of hepatitis C in thalassemic patients and its association with liver enzyme, MOfid Hospital, Iran, 2002. *Pejouhesh* **29**, 213-217 (2005).
109 Alavi, S. M. & Behdad, F. Seroprevalence study of hepatitis C and hepatitis B virus among hospitalized intravenous drug users in Ahvaz, Iran (2002-2006). *Hepatitis Monthly* **10**, 101-104 (2010).
110 Amini, S., Mahmoodabadi, S. A., Lamian, S., Joulaie, M. & Farahani, M. M. Prevalence of hepatitis G virus (HGV) in high-risk groups and blood donors in Tehran, Iran. *Iranian Journal of Public Health* **34**, 41-46 (2005).
111 Ansari, S., Vossogh, P. & Bateni, F. Prevalence of chronic hepatitis in children with leukemia, 1996-2001: A single center experience. *Iranian Red Crescent Medical Journal* **10**, 34-35 (2008).
112 Kazemi Arababadi, M., Hassanshahi, G., Rezaeian, M., Rezazadeh Zarandi, E. & Vazirinejad, R. Evaluation of Occult Hepatits B Virus Infection in Thalassemic Patients Infected by HCV in Kerman Province of Iran. *Journal of Rafsanjan University of Medical Sciences* **8**, 295-302 (2009).
113 Assarehzadegan, M. A., Boroujerdnia, M. G. & Zandian, K. Prevalence of hepatitis B and C infections and HCV genotypes among haemophilia patients in Ahvaz, Southwest Iran. *Iranian Red Crescent Medical Journal* **14**, 3 (2012).
114 Azarbahra, M., Tajbakhsh, E. & Momtaz, H. Phylogenetic analysis of hepatitis delta virus isolated from HBsAg positive patients in Shahrekord, Iran. *Asian Pacific Journal of Tropical Disease* **4**, 391-397 (2014).
115 Azarkeivan, A. *et al.* Evaluation of clinical conditions of thalassemic patients having referred to Adult Thalassemia Center, Tehran. *Sci J Iran Blood Transfus Organ* **8**, 32-41 (2011).

116 Boroujerdnia, M. G., Zadegan, M. A. A., Zandian, K. M. & Rodan, M. H. Prevalence of hepatitis-C virus (HCV) among thalassemia patients in Khuzestan province, southwest Iran. *Pakistan Journal of Medical Sciences* **25**, 113-117 (2009).
117 Broumand, B. *et al.* Prevalence of hepatitis C infection and its risk factors in hemodialysis patients in tehran: preliminary report from "the effect of dialysis unit isolation on the incidence of hepatitis C in dialysis patients" project. *Saudi J Kidney Dis Transpl* **13**, 467-472 (2002).
118 Davarpanah, M. A. *et al.* Hepatitis C Virus Infection in HIV Positive Attendees of Shiraz Behavioral Diseases Consultation Center in Southern Iran. *Indian J Community Med* **38**, 86-91 (2013).
119 Doosti, A., Arnini-Bavil-Olyaee, S., Tajbakhsh, E., Adeli, A. & Mahboudi, F. Prevalence of viral hepatitis and molecular analysis of HBV among voluntary blood donors in west Iran. *New Microbiologica* **32**, 193-198 (2009).
120 Esmaeili, M., Mostafazadeh, A., Sharbatdarn, M., Hajiahmadi, M. & Alijanpoor, M. Hepatitis C in blood products receivers. *Iranian Journal of Pediatrics* **14**, 15 (2004).
121 Faranoush, M. *et al.* Prevalence of hepatitis C resulted from blood transfusion in major thalassemia patients in Semnan, Damghan and Garmsar (2002). *Medical Journal of Hormozgan University* **10**, 77-82.
122 Farshadpour, F., Makvandi, M., Samarbafzadeh, A. R. & Jalalifar, M. A. Determination of hepatitis C virus genotypes among blood donors in Ahvaz, Iran. *Indian Journal of Medical Microbiology* **28**, 54-56 (2010).
123 Ghafourian Boroujerdnia, M., Assarehzadegan, M., Haghirizadeh Rodany, M., Zandian, K. & Noroozkohnejad, R. Detection of molecular markers of hepatitis B, hepatitis C and human immunodeficiency virus (HIV) in thalassemic patients referring to Shafa hospital. *Scientific Medical Journal (AJUMS)* **7**, Pe454-Pe462, En551 (2009).
124 Ghane, M., Eghbali, M., Nejad, H. R., Saeb, K. & Farahani, M. Distribution of hepatitis C virus genotypes amongst the beta-thalassemia patients in north of Iran. *Pakistan Journal of Biological Sciences* **15**, 748-753 (2012).
125 Hassanshahi, G. *et al.* Post-transfusion-transmitted hepatitis C virus infection: A study on thalassemia and hemodialysis patients in southeastern Iran. *Archives of Virology* **156**, 1111-1115 (2011).
126 Joukar, F., Besharati, S., Mirpour, H. & Mansour-Ghanaei, F. Hepatitis C and hepatitis B seroprevalence and associated risk factors in hemodialysis patients in Guilan province, north of Iran: HCV and HBV seroprevalence in hemodialysis patients. *Hepat Mon* **11**, 178-181 (2011).
127 Kalantari, H., Mirzabaghi, A., Akbari, M. & Shahshahan, Z. Prevalence of hepatitis C virus, hepatitis B virus, human immunodeficiency virus and related risk factors among hemophilia and thalassemia patients in Iran. *Iranian Journal of Clinical Infectious Diseases* **6**, 82-84 (2011).
128 Masood, Z., Roghiye, A. & Hasan, N. M. Prevalence of HCV infection in hemodialysis patients of South Khorasan in comparison With HBV, HDV, HTLV I/II, And HIV infection. *Bangladesh Journal of Medical Science* **13**, 36-39 (2014).
129 Kashef, S. *et al.* Antiphospholipid antibodies and hepatitis C virus infection in Iranian thalassemia major patients. *International Journal of Laboratory Hematology* **30**, 11-16 (2008).

130 Khedmat, H. *et al.* Seroepidemiologic study of hepatitis B virus, hepatitis C virus, human immunodeficiency virus and syphilis infections in Iranian blood donors. *Pakistan Journal of Biological Sciences* **10**, 4461-4466 (2007).
131 Makhlough, A., Jamshidi, M. & Mahdavi, M. R. Hepatitis C prevalence studied by polymerase chain reaction and serological methods in haemodialysis patients in Mazandaran, Iran. *Singapore Medical Journal* **49**, 921-923 (2008).
132 Mansoori, S. D. *et al.* Immunological and clinical features of HIV in a group of hospitalized Iranian patients. *Archives of Iranian Medicine* **6**, 5-8 (2003).
133 Mohammad-Alizadeh, A. H., Ghobakhlou, M., Shalmani, H. M. & Zali, M. R. Cholangiocarcinoma: An-eight-year experience in a tertiary-center in Iran. *Asian Pacific Journal of Cancer Prevention* **13**, 5381-5384 (2012).
134 Mansour-Ghanaei, F. *et al.* Seroprevalence of hepatitis B and C among residents of Guilan Nursing Home. *Hepat Mon* **7**, 139-1141 (2007).
135 Motamed, F. *et al.* Evaluation of liver diseases in Iranian patients with primary antibody deficiencies. *Annals of Hepatology* **8**, 196-202 (2009).
136 Mousavi F., A. B. M. A-IFN treatment of hepatitis C in thalassemic patients *Pejouhandeh* **7**, 21-24 (2002).
137 Samimi-Rad, K. *et al.* Prevalence of hepatitis C by RT-PCR in patients with thalassemia and hemophilia in Isfahan province 2005 [Persian]. *Infectious and Tropical Diseases of Iran* **11**, 33-39 (2006).
138 Samimi-Rad, K. & Shahbaz, B. Hepatitis C virus genotypes among patients with thalassemia and inherited bleeding disorders in Markazi province, Iran. *Haemophilia* **13**, 156-163 (2007).
139 Samimi-Rad, K. & Hosseini, M. Hepatitis C virus infection and hcv genotypes of hemodialysis patients. *Iranian Journal of Public Health* **37**, 146-152 (2008).
140 Shamshirsaz, A. A. *et al.* The role of hemodialysis machines dedication in reducing Hepatitis C transmission in the dialysis setting in Iran: A multicenter prospective interventional study. *BMC Nephrology* **5**, 1-5 (2004).
141 Somi, M. H., Ardalan, M. R., Sokhanvar, H., Farhang, S. & Pouri, A. Hepatitis C virus infection in dialysis centers of Tabriz, Iran: a multicenter study. *Archives of Clinical Infectious Diseases* **2** (2007).
142 Zamani, F. *et al.* Prevalence and risk factors of hepatitis C virus infection in Amol city, north of Iran: A population-Based study (2008-2011). *Hepatitis Monthly* **13** (2013).
143 Ziaee, M., Zarban, A., Malekinejad, P. & Akhbary, H. Evaluation of HGV viremia prevalence and its co-infection with HBV, HCV, HIV and HTLV-1 in hemophilic patients of Southern Khorassan, Iran. *Hepat Mon* **7**, 11-14 (2007).
144 Ziaee, M., Power, M., Hosseini, S. & Azarkar, G. Evaluation of hepatitis C infection and its prevalence in hemophilic patients in Khorasan [Persian]. *Horizon of Knowledge* **11**, 54-60 (2005).
145 Ziyaeyan, M., Jamalidoust, M. & Moeini, M. Evaluation of Hepatitis C Virus Infection in Antibody Positive Orphan Newborns. *Jundishapur Journal of Microbiology* **6**, 72-75 (2012).
